# Peptide and Protein Cyclization by a Promiscuous Graspetide Synthetase

**DOI:** 10.1021/acscentsci.5c00408

**Published:** 2025-06-09

**Authors:** Brian Choi, Toby G. Johnson, Arthur Acuña, Hader E. Elashal, A. James Link

**Affiliations:** † Department of Chemical and Biological Engineering, 6740 Princeton University, Princeton, New Jersey 08544, United States; ‡ Department of Chemistry, 6740 Princeton University, Princeton, New Jersey 08544, United States; § Department of Molecular Biology, 6740 Princeton University, Princeton, New Jersey 08544, United States

## Abstract

Macrocyclic peptides have drawn considerable interest as modalities for drug discovery. Graspetides are a class of ribosomally synthesized and post-translationally modified peptides (RiPPs) that harbor one or more macrocycles. These macrocycles are built via side chain-side chain linkages that are installed by ATP-grasp enzymes, giving the peptide family their name. We recently reported on the discovery, structure, and biosynthesis of the graspetide pre-fuscimiditide which is comprised of a stem covalently linked by two ester moieties and a 10 aa loop. Here we probe the substrate tolerance of the fuscimiditide ATP-grasp enzyme, ThfB, and show that it is highly promiscuous. ThfB can cyclize substrates with substitutions to or extensions of the stem region as well as generate multivalent cyclic structures. ThfB also shows remarkable tolerance to substitutions in the loop of pre-fuscimiditide. Loops comprised of flexible glycine-serine sequences ranging from 4 aa to 72 aa were efficiently cyclized by ThfB. Even substrates in which full-length proteins were swapped for the 10 aa loop of pre-fuscimiditide could be cyclized by ThfB. We also show that ThfB can covalently cross-link supramolecularly assembled protein chains. These data show that ThfB is a highly generalizable biocatalyst for both peptide and protein macrocyclization as well for intermolecular protein cross-linking.

## Graphical abstract

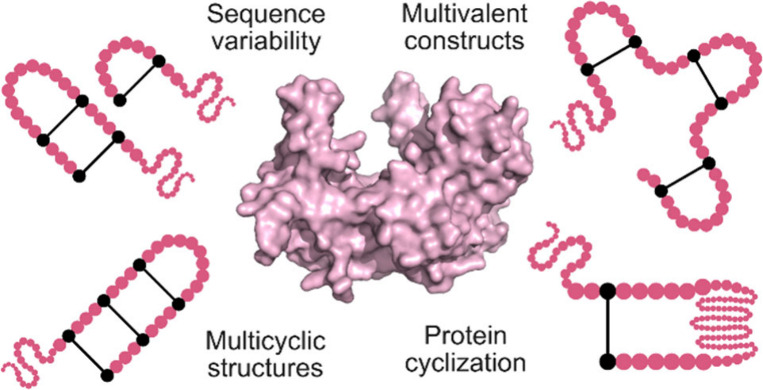

## Introduction

Cyclic peptides are the focus of intense current research, based on their ability to target protein–protein interactions with high specificity, while maintaining desirable pharmacokinetic properties. Lying between the small-molecule and antibody size regimes, cyclic peptides are gaining traction as a new drug modality.^1^ Ribosomally synthesized and post-translationally modified peptides (RiPPs) are a rapidly growing class of natural products, which contain a vast array of cyclic peptides.^2,3^ The graspetides are one such family of RiPPs, which have a macrocyclic structure generated by ester or amide cross-links between pairs of amino acid side chains.^4,5^ The graspetide name comes from the ATP-grasp ligases that catalyze the formation of these cross-links. Like all RiPPs, the precursors to graspetides are gene-encoded and synthesized at the ribosome, allowing for the generation of variants and libraries by protein engineering techniques.^6^ One reason for studying graspetides and their biosynthesis is the idea that their ATP-grasp enzymes can be repurposed as biocatalysts to generate libraries of cyclic peptides.^7–9^

Several different graspetides with varying connectivity patterns have been experimentally characterized (Figure 1).^5^ The most well-studied graspetides are the microviridins, which have two esters and one amide linkage within the 14 amino acid (aa) core peptide.^10^ These three cross-links are staggered so that the peptide is constrained into a compact globular structure. Cross-links may, however, be nested as is the case for OEP6-1,^11^ giving a hairpin stem-loop structure (Figure 1A). Multivalent graspetides are also known, where cyclized sections of the core peptide occur in tandem repeats, such as in plesiocin^12^ and thuringinin^13^ (Figure 1B, 1C). The diverse array of multicyclic peptide structures shown within the graspetide family inspired us to investigate whether a single highly promiscuous ATP-grasp enzyme may be able to tolerate a broad array of precursors to generate this same structural diversity. If a single enzyme were to be found that showed this broad substrate tolerance, one can envision repurposing the graspetide biosynthetic pathway to generate cyclic peptides of a variety of different sequences and sizes in a highly engineerable, recombinant fashion.

**Figure 1:**
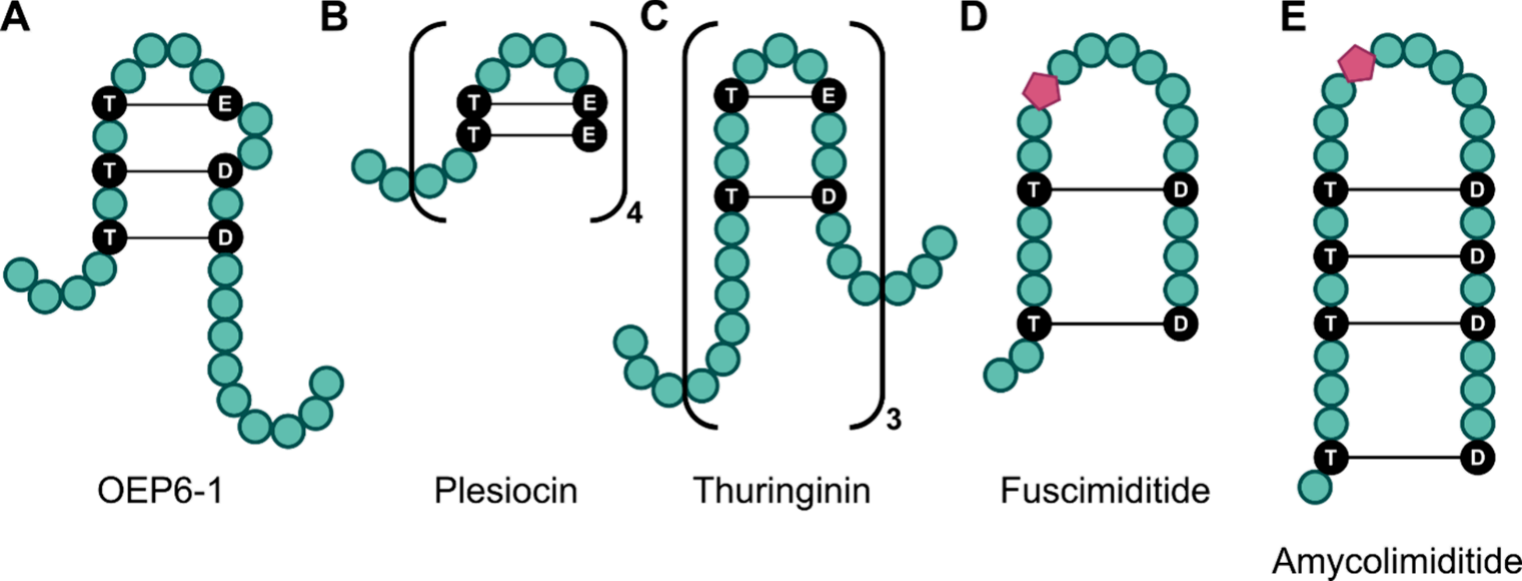
Schematic representation of example graspetides. The core region of each peptide is shown, with amino acids depicted as green circles, ω-ester cross-linked amino acids colored black and aspartimide moieties represented by a pink pentagon. A: OEP6-1 ^11^ has a stem-loop structure with three nested cross-links. B: Plesiocin^12^ is a tetravalent graspetide with two ester cross-links per repeat unit. C: Thuringinin^13^ is a trivalent graspetide with two ester cross-links per repeat unit. D: Fuscimiditide^14^ has a stem-loop structure with two nested cross-links and an aspartimide in the loop. E: Amycolimiditide^15^ has a stem-loop structure with four nested cross-links and an aspartimide in the loop.

Herein, we focus on the engineering of fuscimiditide, a stem-loop structure graspetide characterized by our group,^14^ as a testbed for this idea. The fuscimiditide ATP-grasp enzyme (ThfB) was found to have excellent substrate tolerance to substitutions within the precursor peptide (ThfA). A range of sequence lengths, from 0 – 72 aa, replacing the native 10 aa loop of ThfA, were found to be modified by ThfB into a cyclic structure. Substitutions within the stem region of ThfA were also tolerated, enabling control over the position and number of ester cross-links in the fuscimiditide scaffold. ThfB was able to generate multivalent repeating structures, reminiscent of the other structural classes of graspetides. The broad tolerance of ThfB to loop substitutions even enabled entire proteins to be cyclized when inserted into the loop region of ThfA, revealing potentially no upper limit to the size of macrocycle engineering. This work demonstrates that the fuscimiditide biosynthetic machinery can be repurposed to provide a platform approach to engineering a wide array of cyclic peptides and proteins.

## Results and Discussion

### Structure of Fuscimiditide and Engineering Opportunities

Our group recently published a bioinformatic analysis on a subset of ∼1000 graspetides called graspimiditides,^16^ also known as group 13 graspetides.^17^ Compared to other experimentally characterized graspetides (Figure 1A-C), the graspimiditides (Figure 1D-E) have two distinct differences. First, these peptides harbor an aspartimide moiety introduced via a methyltransferase, leading us to name this subfamily of graspetides the graspimiditides.^16^ Second, graspimiditides contain a larger loop macrocycle, at 10–12 aa, than other examples, which are typically 3 or 4 aa (Figure 1), opening the possibility for significant engineering of these loops. Graspimiditide biosynthesis follows the general logic of RiPP biosynthesis. A ribosomally synthesized linear precursor protein (A) is modified by gene-encoded tailoring enzymes that are found within the RiPP biosynthetic gene cluster (BGC). In the case of fuscimiditide, the founding member of the graspimiditides, the precursor protein (ThfA) is modified by a single ATP-grasp enzyme (ThfB) which introduces two ester cross-links to furnish a species referred to as mThfA^B^ for ThfA modified by ThfB (Figure 2). This cyclized product can then be acted on by a methyltransferase (ThfM) which installs the aspartimide. Like other RiPP precursors, graspimiditide precursors carry an N-terminal leader peptide that must be removed to generate the mature RiPP. The native protease enzymes have not yet been determined for any graspimiditides, but trypsin has been utilized to remove leader peptides.^14,18^ Trypsin can also cleave mThfA^B^ to furnish the graspetide pre-fuscimiditide which is identical to fuscimiditide except that it lacks the aspartimide (Figure 2). Here we focus our efforts on engineering the fuscimiditide core peptide sequence, comprised of a stem and a loop. The stem region of fuscimiditide is defined as the region between the inner (Thr-7 to Asp-18) and outer (Thr-3 to Asp-22) ester cross-links, while the loop comprises residues Tyr-8 to Ser-17 bound by the inner cross-link (Figure 2). Some engineering of other graspetide loop regions has been carried out involving single or double amino acid substitutions.^19,20^ However, more ambitious engineering attempts, such as changing loop sizes, have so far been unsuccessful in other graspetides.^5^

**Figure 2:**
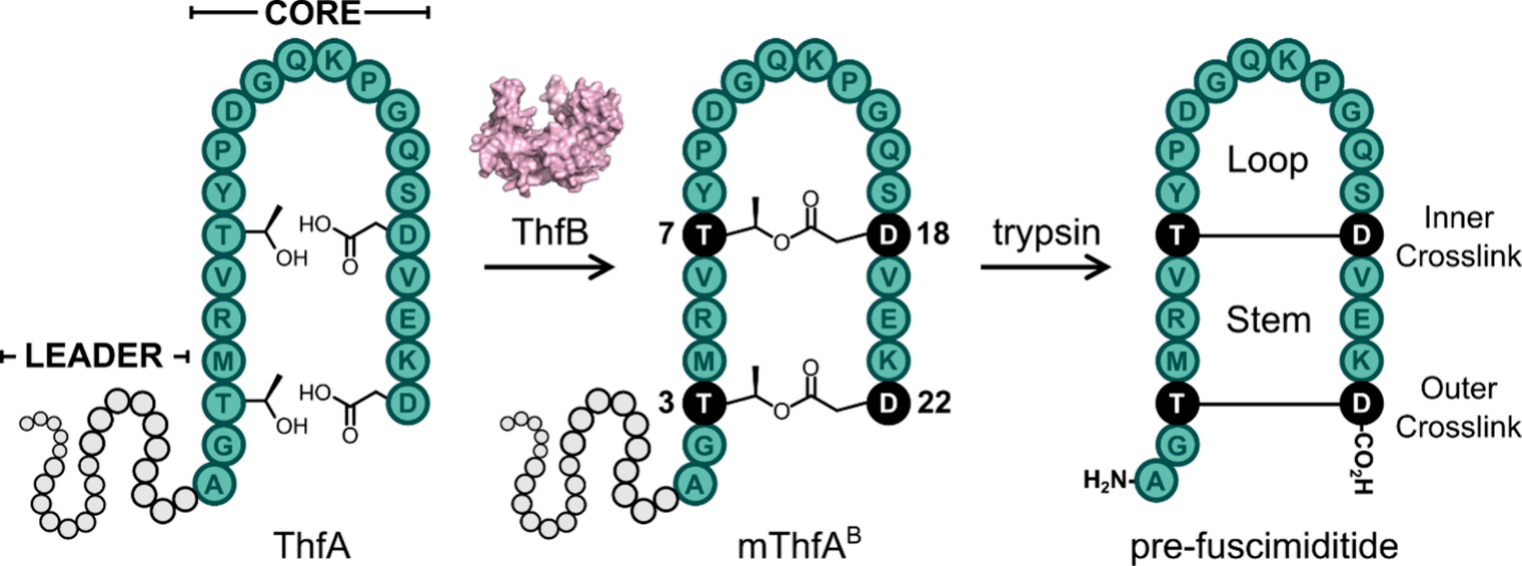
Schematic of pre-fuscimiditide biosynthesis. The ribosomally synthesized linear precursor protein (ThfA) is comprised of an N-terminal leader sequence (gray) and a C-terminal core region (green). ThfA is phosphorylated by the ATP-grasp enzyme (ThfB), which installs two ester cross-links, defined as the inner (Thr-7 to Asp-18) and outer (Thr-3 to Asp-22) cross-links, giving mThfA^B^. Trypsin cleaves the leader sequence to give the 22 aa bicyclic peptide pre-fuscimiditide.

### Engineering the Loop Region of Pre-fuscimiditide

We first sought to investigate the loop sequence tolerance of pre-fuscimiditide. Variants of ThfA were generated by substituting the interstitial residues of the loop region with varying lengths of flexible Gly-Ser repeats, ranging from 0 (corresponding to loop deletion) to 72 aa residues (Figure 3A). Constructs ThfA1-10 were each coexpressed with ThfB in *E. coli* and analyzed by LC-MS. Dehydration of ThfA1-10 corresponded to ester formation by ThfB. The net loss of water results from the enzymatic phosphorylation of an acidic residue forming a mixed anhydride, which upon intramolecular nucleophilic attack by a side chain alcohol generates an ω-ester. Astonishingly, the ThfA1-10 precursors were all found to be at least singly dehydrated (Figure 3B), implying that all variants were post-translationally modified by ThfB into a macrocyclic structure. Variants ThfA4-8 with loop lengths of 4 to 12 aa (compared to 10 aa for native ThfA) were fully modified with two dehydrations observed (Figure 3B). Notably, cyclization was observed with an odd number of residues (ThfA7, 9 aa loop) and not just even-numbered sequences, as in native fuscimiditide. Complete modification of these engineered constructs demonstrates the unprecedented promiscuity of ThfB in accommodating changes to the loop size of the precursor peptide. The use of the flexible GS-repeat sequence also emphasizes the broad engineerability of the loop region, which may have the potential to harbor almost any sequence.

**Figure 3:**
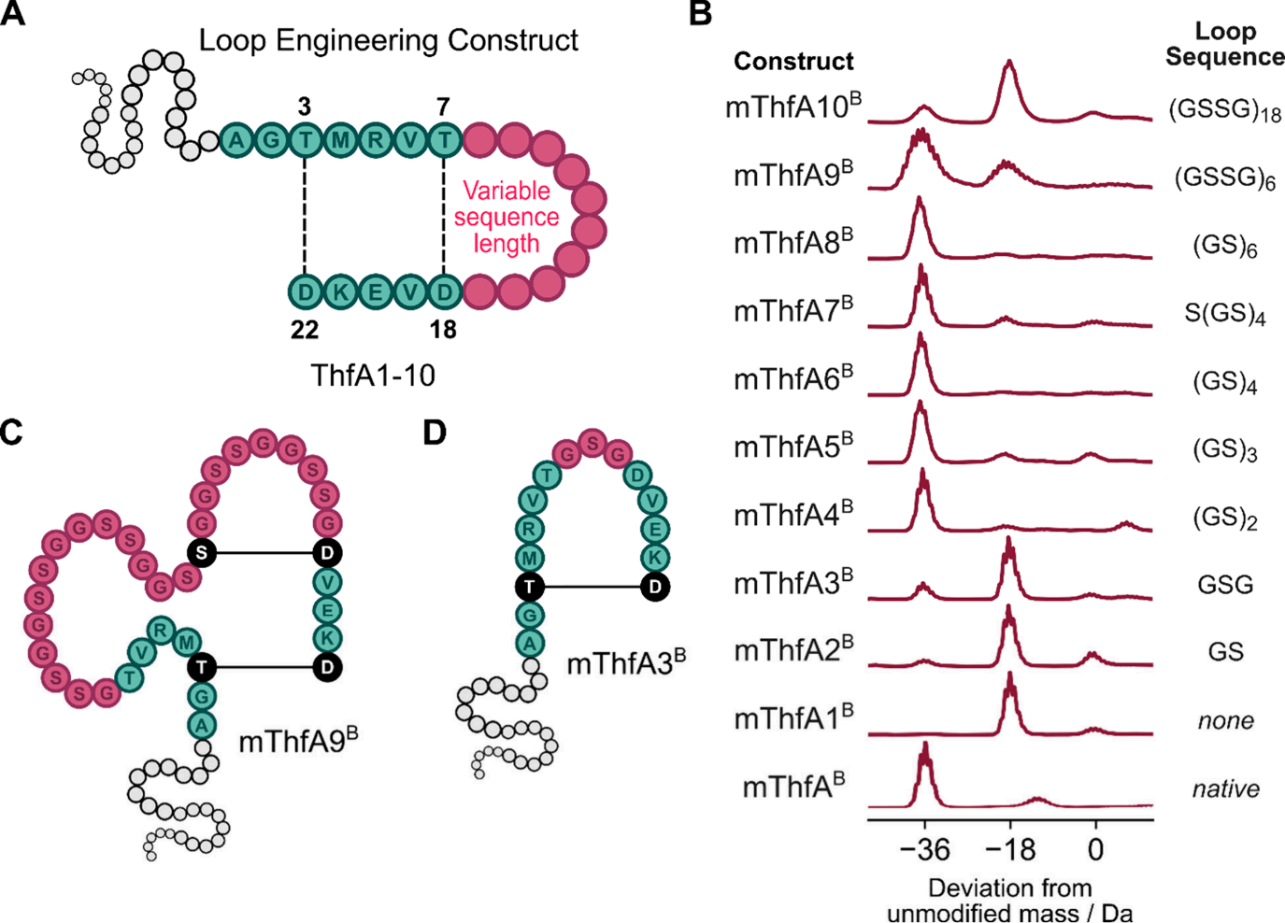
Engineering the loop of pre-fuscimiditide to access cyclic peptides. A: General structure of the loop-substituted pre-fuscimiditide precursor where the pink unlabeled amino acids are replaced with various loop sequences. B: Deconvoluted mass spectra of ThfB-modified constructs ThfA1-10 with varying lengths of GS-repeats in place of the loop macrocycle compared to mThfA^B^. C: Structure of doubly ester cross-linked mThfA9^B^, harboring a non-native Ser-Asp cross-link, as demonstrated by MS/MS analysis, see Figure S1. D: Structure of singly cross-linked mThfA3^B^, containing only the native outer cross-link, as demonstrated by MS/MS analysis (Figure S2).

ThfA9, with a 24 aa loop, was mainly 2-fold dehydrated by ThfB, but some singly dehydrated product was observed. LC-MS analysis of the trypsin digestion products of mThfA9^B^ revealed that modification had occurred within the core peptide. A small amount of the desired product was observed, but the major doubly dehydrated cleavage product revealed a specific non-native Ser-Asp ω-ester linkage (Figure 3C, Figure S1). Despite forming the native outer cross-link correctly, an ω-ester linkage between the native Asp18 (numbered according to native fuscimiditide) and a serine in the flexible GSSG-repeat linker was observed to be the major species of the doubly modified construct. The linker presumably accommodated a conformation which promoted reaction of the non-native Ser, rather than the native Thr7 with Asp18. Although the GS-repeat sequence was selected for the inherent flexibility and low propensity to form secondary structures, the presence of nucleophilic Ser residues enabled non-native cross-links to form. With success in cyclizing a 24 aa loop, more than twice the size of the native 10 aa loop, we also attempted to cyclize a 72 aa (GSSG)_18_ loop in the construct ThfA10. The major product of ThfB-modified ThfA10 (72 aa loop) was a singly dehydrated product showing that ThfB can accommodate loops much larger than the native loop size of 10 aa. Despite the increased number of nucleophilic Ser residues in the larger engineered loop variants, ThfA9 and ThfA10, modification efficiency decreased suggesting that the increased entropic penalty to cyclization of larger ring sizes may also contribute to the lower level of esterification in these variants. We considered the possibility that the long-loop constructs ThfA9 and ThfA10 might react intermolecularly to form dimers or higher order oligomers, but no such species were observed by mass spectrometry.

Reducing the size of the loop macrocycle to 3 aa or fewer reduced the ability of ThfB to modify constructs beyond a single dehydration (Figure 3B). Variants ThfA1-3 were found to be mainly singly dehydrated when coexpressed with ThfB. Trypsin digestion of mThfA3^B^ yielded the singly dehydrated core peptide fragment, which, following MS/MS analysis, confirmed formation of the outer cross-link (Figure 3D, Figure S2). Notably, this cyclized core peptide fragment is resistant to trypsin digestion despite harboring one Lys and one Arg residue, showing the stabilizing effect of cyclization. The ability of ThfB to form the outer ester cross-link without forming the inner cross-link is unique among graspetides. In general, cross-linking proceeds N-to-C-terminally following the electrophilic carboxyl residues,^11,15,16,20^ and mutational studies have previously found that substitutions at the inner ω-ester linkage of other graspetides abolished all subsequent ester formation.^15,20^ Since macrocyclization of the short loop variants ThfA1-3 occurred solely at the outer cross-link, we envisioned that the inner cross-link did not form due to steric constraints. Macrocycles with fewer than 4 interstitial residues were not observed, perhaps establishing a minimum loop size for pre-fuscimiditide variants.

The remarkable substrate tolerance of ThfB to enzymatically form macrocyclic peptides with a broad range of loop sequence lengths exemplifies the promiscuous nature of this ligase enzyme. Previous studies involving point mutations in microviridins, which conserved the native tricyclic structure were found to modulate protease inhibition activity.^19^ However, attempts to change the ring size by even a single amino acid in microviridin K, resulted in no cyclic product being formed.^20^ Interestingly, cyclization of amycolimiditide, a different “long-loop” graspetide (Figure 1E), was shown to be possible with loop sizes of 10 (native size), 11, or 12 aa following the insertion of glycine residues.^15^ The promiscuous ATP-grasp enzyme ThfB provides a platform approach to cyclizing a remarkable range of different peptide macrocycle sizes.

### Engineering the Stem Region of Pre-fuscimiditide

We next targeted substitutions within the stem region of ThfA (Figure 4A), to evaluate how tolerant this region was to engineering. Variants ThfA11 and ThfA12 replaced each cross-linking pair with unreactive residues to investigate whether cross-links could form independently of one another. As we demonstrated above, the outer cross-link in mThfA3^B^ was installed in the absence of the inner linkage (Figure 3B). Interestingly, both ThfA11 and ThfA12 yielded completely singly dehydrated products when coexpressed with ThfB (Figure 4B). Hence, ThfB can form either cross-link in ThfA without the need for the other, which to our knowledge is the first graspetide synthetase to display this versatility to install cross-links in any order. Variant ThfA13, with the N-terminal sequence (TMRVT) swapped with the C-terminal sequence (DVEKD) in the stem macrocycle, yielded no dehydrated products when coexpressed with ThfB (Figure S3). Graspetides typically have N-terminal nucleophilic residues (Thr/Ser/Lys) that cross-link with C-terminal electrophilic residues (Asp/Glu),^4^ so it was not surprising that variant ThfA13 with cross-linking residues in reverse order was shown to not be enzymatically modified.

**Figure 4:**
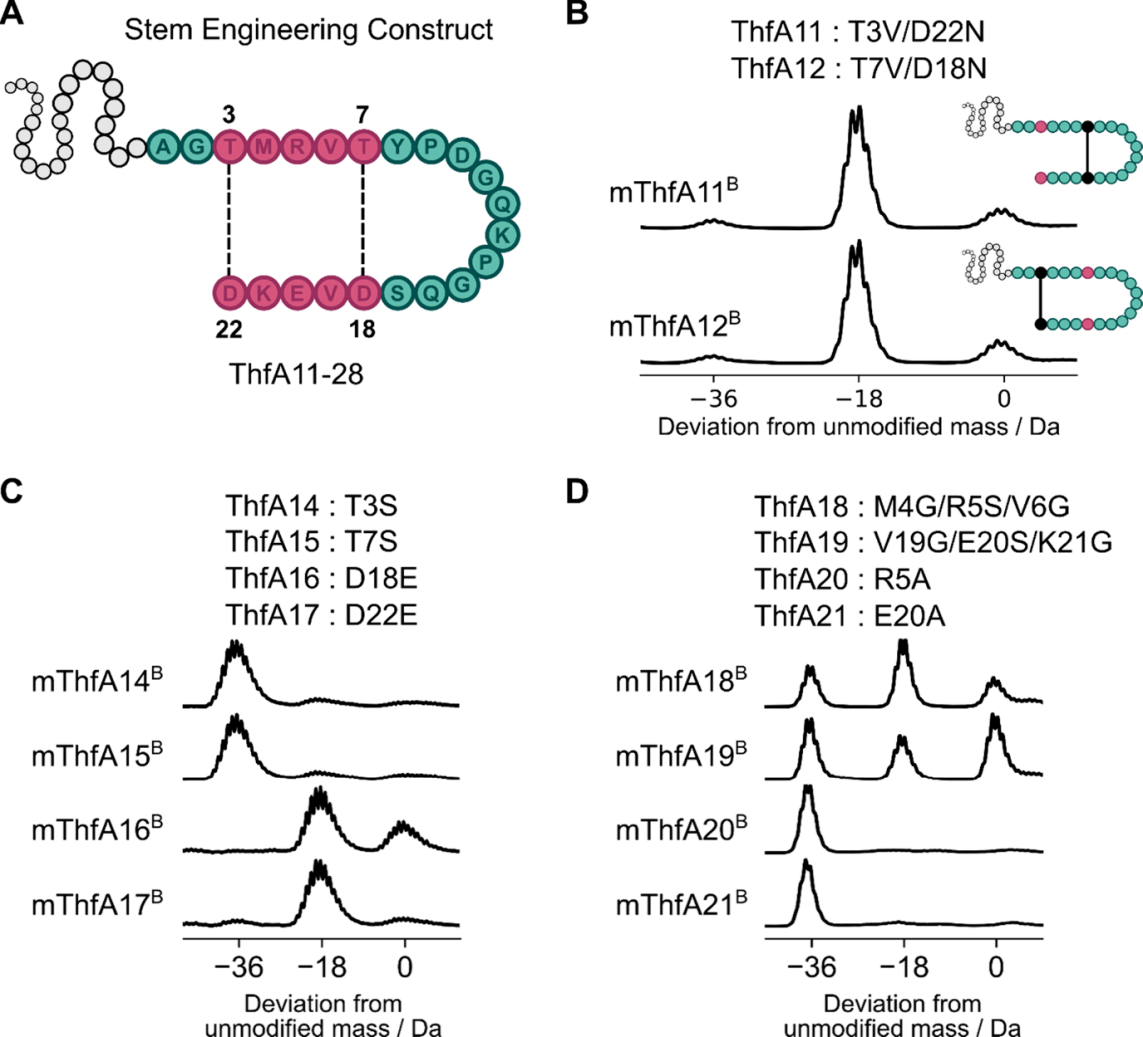
Assessing the tolerance of pre-fuscimiditide macrocyclization to amino acid substitutions in the stem. A: General structure of the stem substituted pre-fuscimiditide precursor. Pink amino acids comprise the stem and are varied in this set of constructs. B: Mass spectra of ThfA variants with amino acid substitutions targeting each cross-link pair, showing that the inner and outer cross-links can form independently. C: Mass spectra of variants with substitutions in individual residues involved in cross-links. While Thr to Ser substitutions are tolerated, Asp to Glu substitutions are not. D: Mass spectra of ThfA variants with substitutions to the interstitial amino acids of the stem macrocycle.

Point substitutions (T3S, T7S, D18E, and D22E) in variants ThfA14-17 were designed to evaluate if non-native residues could form cross-links. Natively ThfB catalyzes the esterification of threonine with aspartic acid. Variants ThfA14-15 exhibited complete 2-fold dehydration, demonstrating that serine-aspartate esters were formed as efficiently as the native threonine-aspartate esters *in vivo* (Figure 4C), in keeping with the observation of this non-native cross-link in the loop-extended variant mThfA9^B^. In contrast, ThfA16-17 displayed only up to one-fold dehydration (Figure 4C). Incubation of purified samples of mThfA16^B^ and mThfA17^B^ with hydrazine, which has been shown to react with esters to give hydrazide-tagged acidic residues,^15^ revealed only the native Asp residues and not the substituted glutamic acids were able to form ester cross-links based on MS/MS analysis (Figure S4, S5). ThfB was tolerant to the substitution of nucleophilic residues in the precursor peptide, but not the electrophilic residues. The chemoselective promiscuity of ATP-grasp enzymes that natively install esters to substitutions of the nucleophilic residues has previously been shown to generate amides and thioesters efficiently.^18,21^ The inability to vary the electrophilic residue may be the result of the ATP-grasp enzyme not phosphorylating the substituted residue or the electrophilic residue not reacting due to steric hindrance.

The ability to substitute the interstitial amino acids between cross-links in the stem was investigated by replacing the three amino acids in each section independently with flexible GSG sequences. Upon coexpression *in vivo* with ThfB, variants ThfA18 and ThfA19 exhibited 2-fold dehydration, albeit with appreciable amounts of singly dehydrated and unmodified species present (Figure 4D). Notably, the proportion of fully modified product for stem variants ThfA18 and Thfa19 was reduced compared to the loop variants ThfA1-10. We considered that the reduced cross-linking efficiency may result from the disruption of a potential salt bridge between Arg5 and Glu20 within the stem macrocycle. However, when R5A or E20A point substitutions in variants ThfA20 and ThfA21, respectively, were generated and coexpressed with ThfB, complete 2-fold dehydration was observed (Figure 4D). This suggests that a potential salt bridge within the stem macrocycle does not significantly influence the activity of ThfB. Instead, the improved modification efficiency of the more conservative mutations suggests that recognition of the entire stem region of the precursor by ThfB may influence enzyme activity. Indeed, variants ThfA22-24 with the loop and Thr7 and/or Asp18 deleted, were found to be unmodified when coexpressed with ThfB. (Figure S6). Further exemplifying that loss or too significant a change to the stem region appears to be deleterious to the ability of ThfB to modify the construct. Variants ThfA25-28, which altered the size of the stem macrocycle by one or two residues were mostly poorly tolerated by ThfB (Figure S7), equivalent to what was previously attempted, and unsuccessful, for microviridins.^20^ An exception to this trend was variant ThfA26 which was well-modified by ThfB into a graspetide with an asymmetric stem (Figure S7). In general, while the stem of ThfA can tolerate small changes in its sequence, it is less tolerant to substitution than the loop sequence.

### Extension of the Multicyclic Stem Region

We were interested in the question of whether the stem of pre-fuscimiditide could be extended with the addition of a third ester cross-link. Inspired by a putative graspetide precursor from *Thermobifida alba,* which we predicted may form three ester cross-links (Figure S8), ThfA29 was designed. With two additional C-terminal residues, S23 and D24, and threonine in place of Ala1, we envisioned that ThfA29 may be modified by ThfB to form a tricyclic structure. Coexpression of ThfA29 with ThfB yielded a major product with three dehydrations consistent with a tricyclic structure (Figure 5A), though some doubly dehydrated product was still observed. Constructs ThfA30 and ThfA31 probed the tolerance of installing the third ω-ester linkage in different positions of ThfA. When coexpressed with ThfB, ThfA30 and ThfA31 were only dehydrated twice (Figure 5A), revealing the inability to form a third macrocycle when cross-links were immediately adjacent to one another (as in plesiocin) or within the existing stem region. ThfA32 was designed based on replicating the sequence of the stem macrocycle. The four preceding amino acids to T3 were substituted with the N-terminal sequence of the loop macrocycle (TMRV) and a repeat of the C-terminal sequence (VEKD) was added after D22. Gratifyingly, ThfB predominantly modified ThfA32 3-fold, consistent with three ω-ester cross-links. Further constructs based on the structure of ThfA32, which substituted the native interstitial stem residues within the two stem macrocycles of the tricyclic framework were found not to be fully modified by ThfB (constructs ThfA33-37 in Figure S9). This demonstrated that while ThfB could install more than the native two ester cross-links in engineered ThfA constructs, specific sequence recognition of the interstitial amino acids in the stem region allowed only conservative substitutions to be tolerated.

**Figure 5:**
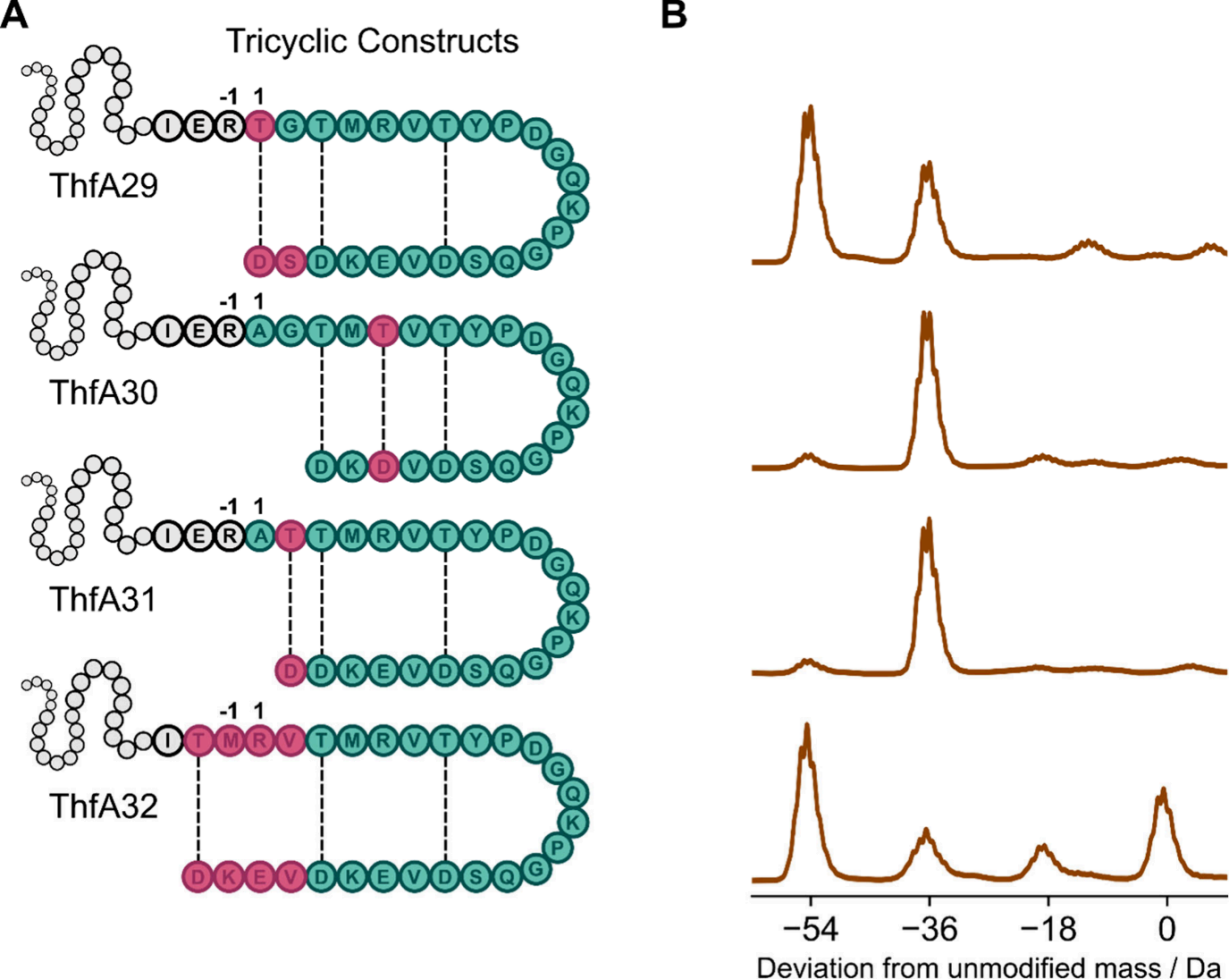
Engineering an additional ω-ester cross-link into pre-fuscimiditide. A: Structures of the tricyclic constructs ThfA29-32 with the native N-terminal leader (gray) and core peptide (green) where amino acids altered from the native sequence are highlighted in pink, with expected cross-links depicted as dashed lines. B: Deconvoluted mass spectra of ThfB modified ThfA29-32 (top to bottom), where a −54 Da peak shift from the unmodified mass indicates formation of the desired three ester cross-links.

### Generating Multivalent Tandem Repeat Cyclic Peptides

Fuscimiditide, like the other graspimiditides, contains nested macrocycles forming a stem-loop structure.^16^ However, other graspetides such as plesiocin,^12^ thuringinin,^13^ chryseoviridin,^22^ and AMdnA^23^ have repeating macrocyclic units (see Figure 1). This prompted us to investigate whether the fuscimiditide ATP-grasp enzyme could construct multivalent core peptides in tandem. Divalent constructs ThfA38-44 were designed, with the leader and core peptide, followed by a linker sequence and a second core peptide unit (Figure 6A). A GDPSAG linker sequence was utilized in construct ThfA38, replicating the linker sequence in plesiocin.^12^ ThfB esterification of ThfA38 yielded 3-fold dehydration as the major product, with 4-fold dehydration also observed. Suspecting that flexibility and length of the linker sequence would significantly affect esterification efficiency, a panel of Ser-Gly-repeating linkers from 0 to 10 residues were tested (ThfA39-44). Of all the linker lengths tested for the divalent constructs, the highest ratio of 4-fold to 3-fold dehydration was observed in ThfA40, with the 6 amino acid (SG)_3_ linker. The increased proportion of 4-fold esterified product observed for construct ThfA40 compared to ThfA38, suggests that the increased flexibility of the (SG)_3_ linker compared to the GDPSAG linker assists ThfB in modifying multivalent core peptides.

**Figure 6:**
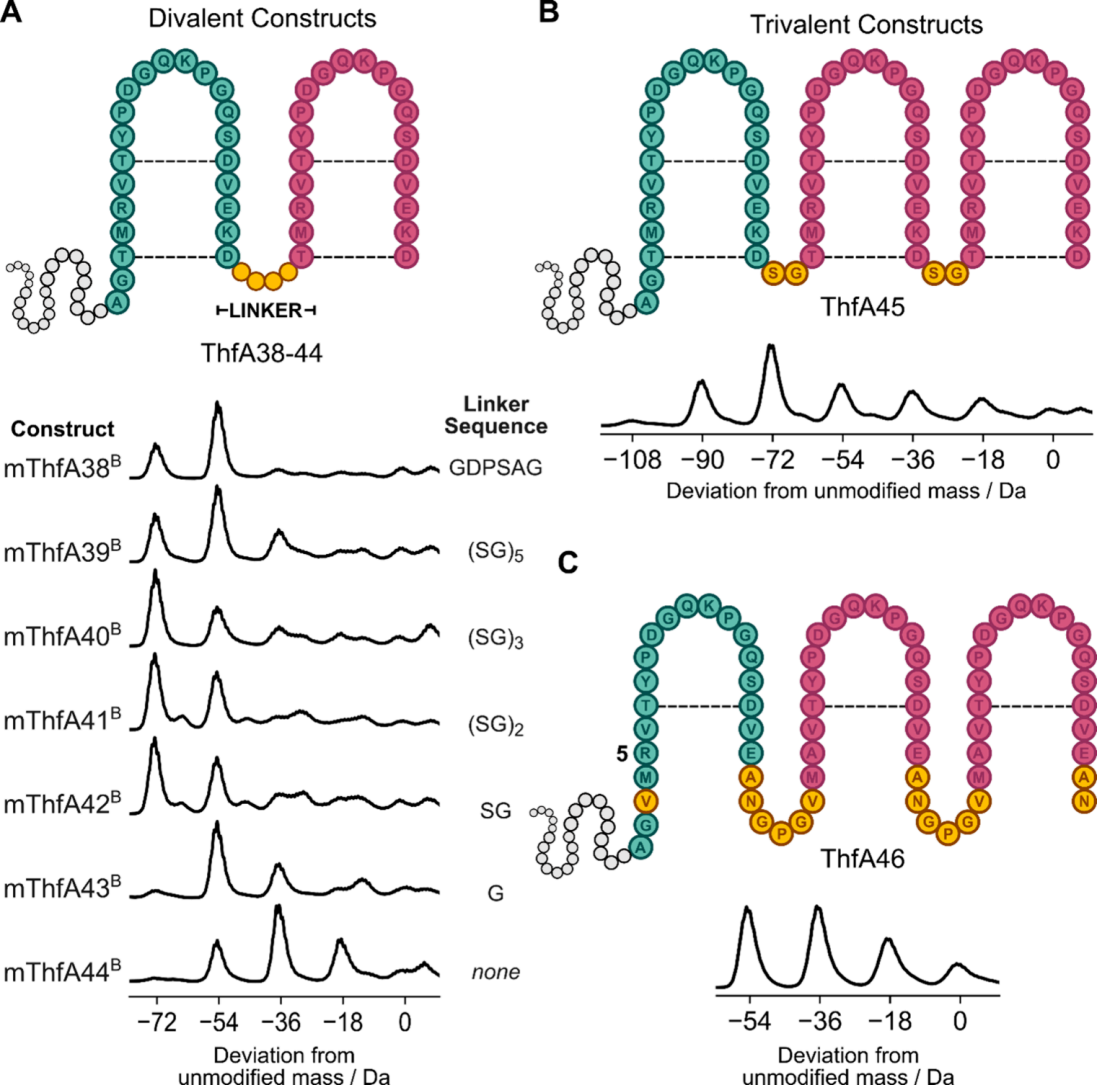
Generating multivalent peptides from pre-fuscimiditide. A: General structure of the divalent construct with the native N-terminal leader (gray) and core peptide (green) attached via different linkers (yellow) to the second core peptide (pink), with expected cross-links depicted as dashed lines. Deconvoluted mass spectra of ThfB modified ThfA38-44, where a −72 Da peak shift from the unmodified mass indicates formation of the desired four ester cross-links. B: Structure of trivalent construct ThfA45 with the native N-terminal leader (gray) and core peptide (green) attached to two repeats of a SG-linker (yellow) core peptide (pink) sequence, with expected cross-links depicted as dashed lines. Deconvoluted mass spectra of mThfA45^B^, where a −108 Da peak shift from the unmodified mass indicates a 6-fold ester cross-linked product. C: Structure of trivalent construct ThfA46 with the native N-terminal leader (gray) and core peptide (green) attached to two repeats of a GPG-linker (yellow) core peptide (pink) sequence, with expected cross-links depicted as dashed lines. Amino acids which were mutated from the native sequence were also highlighted in yellow. Deconvoluted mass spectra of mThfA46^B^, where a −54 Da peak shift from the unmodified mass indicates a 3-fold ester cross-linked construct.

Following our success in generating divalent peptides with ThfB, we sought to construct trivalent structures. In variant ThfA45, the ThfA sequence was appended with two additional fuscimiditide core peptide units connected via Ser-Gly linkers (Figure 6B). After coexpression with ThfB, up to 5-fold dehydration was observed (Figure 6B), one dehydration fewer than expected for the fully dehydrated peptide. To reduce the number of potential cross-links in the trivalent peptide, construct ThfA46 was designed by substituting the outer cross-link Thr and Asp residues to Val and Asn, respectively, as in construct ThfA11. The internal trypsin cut sites were also substituted with Ala residues, and a Gly-Pro-Gly linker was utilized in between the core repeats. These substitutions were introduced to aid the structural characterization of ThfA46, expecting the proline effect^24^ to assist with mapping the ester linkages by tandem mass spectrometry. ThfB modification of ThfA46 resulted in a significant proportion of completely (3-fold) dehydrated product (Figure 6C). Trypsin digestion of mThfA46^B^ yielded the Arg5 cleavage product with 3-fold dehydration and the expected trivalent tandem repeat structure as confirmed by mass spectrometry (Figure S10, S11). Hence, a simplified design with fewer possibilities of unintended linkages enabled multivalent peptides to be synthesized with ThfB more efficiently. Collectively this data shows that ThfB’s promiscuity extends to multivalent peptides as well, even highly engineered variants such as ThfA46. However, conversion of the trivalent constructs into their fully esterified forms is lower than that of the divalent constructs.

### Enzymatic Cyclization of Proteins

The ability of ThfB to esterify a core peptide with a highly expanded loop (ThfA10, 72 aa) suggested that the enzyme might be able to modify a core peptide with even longer sequences inserted into the loop, such as a whole protein. As a proof-of-concept, three fluorescent proteins (abbreviated FP; mRuby2,^25^ 235 aa; superfolder green fluorescent protein (sfGFP),^26^ 236 aa; and mTurquoise2, 237 aa)^27^ were each inserted into the loop of ThfA, to investigate whether ThfB could enzymatically cyclize entire proteins. N- and C-terminal (GS)_3_ linkers were added to flank the FP sequence to elongate the protein termini for cyclization (Figure 7A). The FP constructs we used all had a Lys residue at their C-termini, providing a trypsin digestion site for structural analysis of the cross-linked products. The FP-inserted ThfA constructs ThfA47-49 were first expressed in the absence of ThfB and the products analyzed by mass spectrometry. A −22 Da shift was observed for ThfA47 (Figure 7B), consistent with the spontaneous formation of the mRuby2 chromophore via dehydrative cyclization and two oxidations.^28^ Formation of the chromophores in sfGFP and mTurquoise2 occur via spontaneous dehydrative cyclization and a single oxidation step, resulting in a net −20 Da shift.^29,30^ For constructs ThfA48 and ThfA49 a −22 Da shift was observed (Figure 7B), consistent with the formation of the chromophore and an internal disulfide bond (Figure S12).

**Figure 7:**
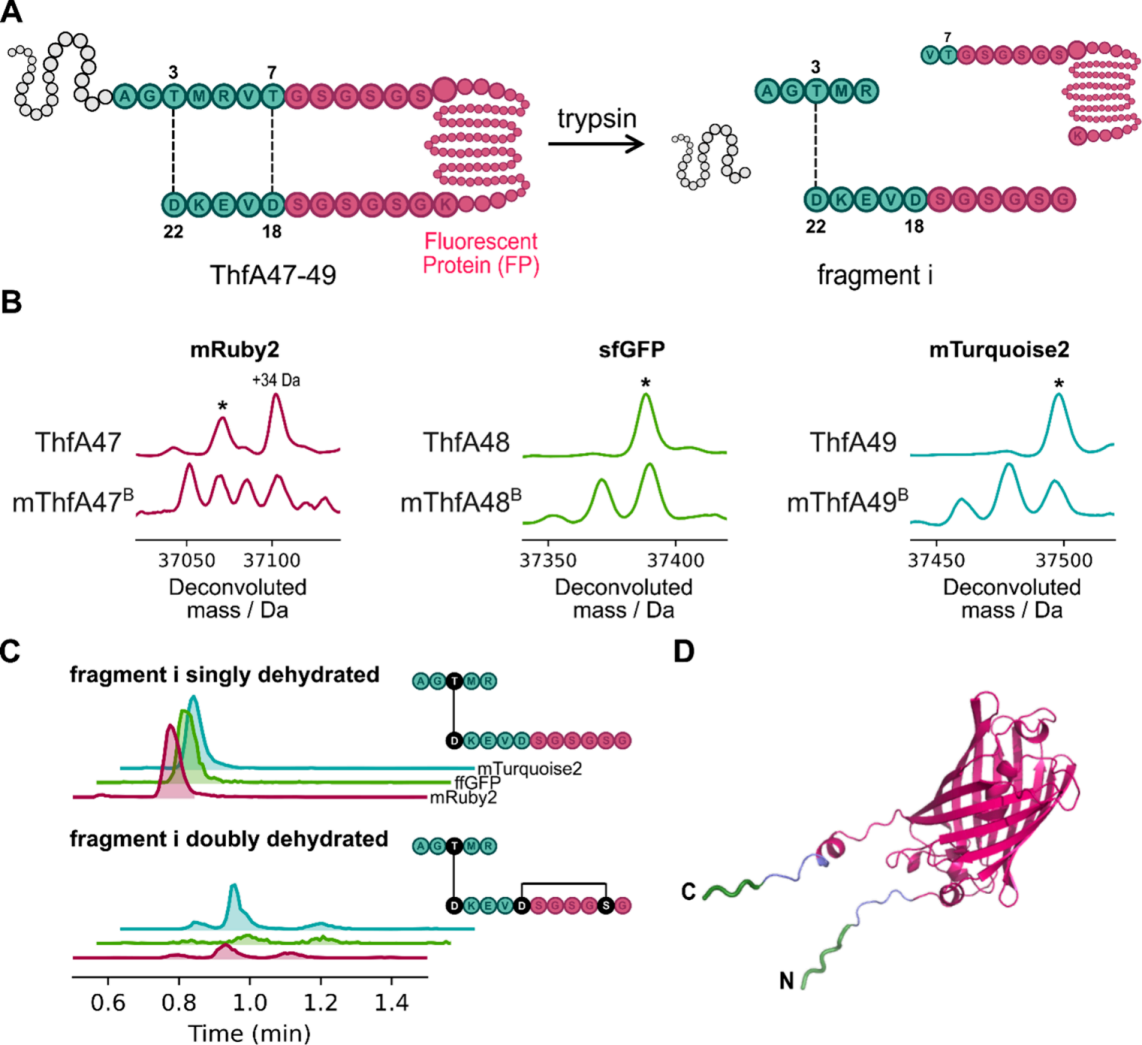
ThfB can cyclize entire proteins. A: General structure of the cyclic protein constructs with the native N-terminal leader (gray) and core peptide (green) with engineered loop (pink) containing linker sequences and the fluorescent protein, with expected cross-links depicted as dashed lines. The expected major fragments from trypsin digestion are shown, where fragment i is only possible if the outer cross-link has formed correctly. B: Mass spectra of FP constructs ThfA47-49 expressed with and without ThfB. The peak marked by an asterisk symbol (*) represents the species with a mature chromophore and no ester cross-links. The +34 Da peak in mRuby2 corresponds to an intermediate in chromophore maturation. C: Extracted ion chromatograms of tryptic fragment i with one (1552.68 Da) and two (1534.67 Da) dehydrations. Structures of tryptic fragment i with one and two dehydrations are shown with cross-links as determined by MS/MS analysis (Figure S16, S17). D: AlphaFold 3 model of ThfA49 without the leader peptide. The core peptide sequence is colored green, linkers are blue and mTurquoise2 is cyan. Similar models for the other two fluorescent protein constructs are in Figure S13.

ThfB was coexpressed with each of the FP constructs ThfA47-49, to evaluate whether entire proteins could be cyclized. Products with mass peaks corresponding to one-fold dehydration relative to the mature chromophores were observed for all three FPs, suggesting that all FP constructs were modified by ThfB (Figure 7B). While some unmodified protein was observed for all 3 FPs, the mTurquoise construct ThfA49 could be doubly dehydrated (Figure 7B). The structures of ThfA47-49 with and without the leader peptide were modeled using AlphaFold 3 ^31^ to visualize the position of the N- and C-termini of the FPs and the alignment of the fuscimiditide stem residues (Figure S13). The AlphaFold 3 model of ThfA49 without the leader peptide highlights the proximity of the fuscimiditide stem residues for effective cross-linking, shown in green in Figure 7D. Given that the linker sequences were not optimized and length and steric considerations in whole protein constructs will likely be more important, compared to the smaller peptide variants ThfA1-10, it is remarkable that modified proteins were observed at all. Measuring the fluorescence emission of mRuby2, ThfA47 and mThfA47^B^ in a Tris-buffered solution revealed no difference in intensity between constructs, suggesting that modification did not influence the chromophore of the FP (Figure S14). Monitoring the change in fluorescence emission of the linear protein ThfA47 and cyclized mThfA47^B^ after heating (0–5 min, 90 °C) revealed no detectable change in thermal stability (Figure S15).

We next turned to mass spectrometry to confirm that the singly dehydrated FPs were truly cyclized; we wanted to ensure that undesired ester formation between the C-terminal stem and the Gly-Ser linker was not occurring as we saw for mThfA9^B^ (Figure 3). To map the ester linkages in ThfB modified ThfA47-49, we analyzed their tryptic fragments by mass spectrometry, which were expected to contain the characteristic fragment i if the outer cross-link had formed (Figure 7A). Ions corresponding to the singly dehydrated fragment i were observed in trypsin digested samples of ThfB-modified ThfA47-49 (Figure 7C), confirming that all FP-constructs were effectively cyclized between their N- and C-termini as designed. MS/MS analysis of the singly dehydrated fragment i from trypsin digested mThfA47^B^ was utilized to confirm the position of cross-linking (Figure S16). Since Thr3 was the only nucleophilic residue in the N-terminal section (AG**T**MR) of fragment i, this residue must have formed a cross-link with an electrophilic amino acid in the C-terminal section (GSGSGSDVEKD). MS/MS analysis of the singly dehydrated fragment i yielded a critical *y*_10_ fragment connected to AGTMR with a −18 Da shift. This ion, detected in the MS/MS spectra revealed that the AGTMR section was cross-linked with Asp22, confirming the presence of the native outer cross-link. CID analysis of the doubly dehydrated fragment i from trypsin digested mThfA49^B^ also yielded the same *y*_10_ fragment revealing the same outer cross-link (Figure S17). 2-Fold dehydration of fragment i suggested that Asp18 was also cross-linked, likely forming a 6 aa macrocycle with a Ser in the C-terminal section as suggested by CID analysis (Figures 7C, S16, S17). Although this cross-link demonstrates that protein constructs can be doubly esterified by ThfB, this non-native cross-link does not contribute to the cyclization of the protein.

Having demonstrated that a range of polypeptide sequences could be intramolecularly cyclized by ThfB, we were interested in whether intermolecular cross-linking could be enzymatically catalyzed. Natively ThfB installs two intramolecular ester cross-links in ThfA and in all previous constructs (ThfA1-49) no oligomeric products were observed. In order to preorganize the amino acids which natively form cross-links (Thr-7 to Asp-18 and Thr-3 to Asp-22) the antiparallel coiled-coil homodimer of Beclin1 ^32,33^ was utilized in construct ThfA50. This construct, comprising two separate polypeptide chains, was designed to incorporate a coil from the Beclin1 coiled-coil domain into each fragment. Self-assembly within the cell may then facilitate the preorganization of the two proteins into an orientation such that ThfB would intermolecularly cross-link the two proteins (Figure 8A). Remarkably, the in-cell supramolecular assembly of the two protein fragments facilitates the intermolecular cross-linking of ThfA50-coil 1 with ThfA50-coil 2 to yield the covalently cross-linked protein dimer (mThfA50^B^).^34^ Native purification from the cell lysate using Ni-affinity chromatography yielded two major peaks in the total ion chromatogram (Figure 8B). The first peak included ThfA50-coil 1 and mThfA50^B^ in a ratio of ∼1:1, as well as ThfA50-coil 2 (Figure 8C). Native purification enabled the retention of ThfA50-coil 2 during Ni-affinity chromatography, despite not having a His-tag, presumably through binding to the Beclin1 domain in ThfA50-coil 1. The second peak in the total ion chromatogram was ThfB (Figure 8B, Figure S18), which was likely retained as a result of binding to the leader sequence of ThfA50-coil 1, as previously observed.^14^ Interestingly, 2-fold dehydration was the major product for mThfA50^B^ (Figure 8C) demonstrating that ThfB was able to effectively mediate intermolecular cross-linking of two proteins through supramolecular self-assembly of a coiled-coil dimer within the cell.

**Figure 8:**
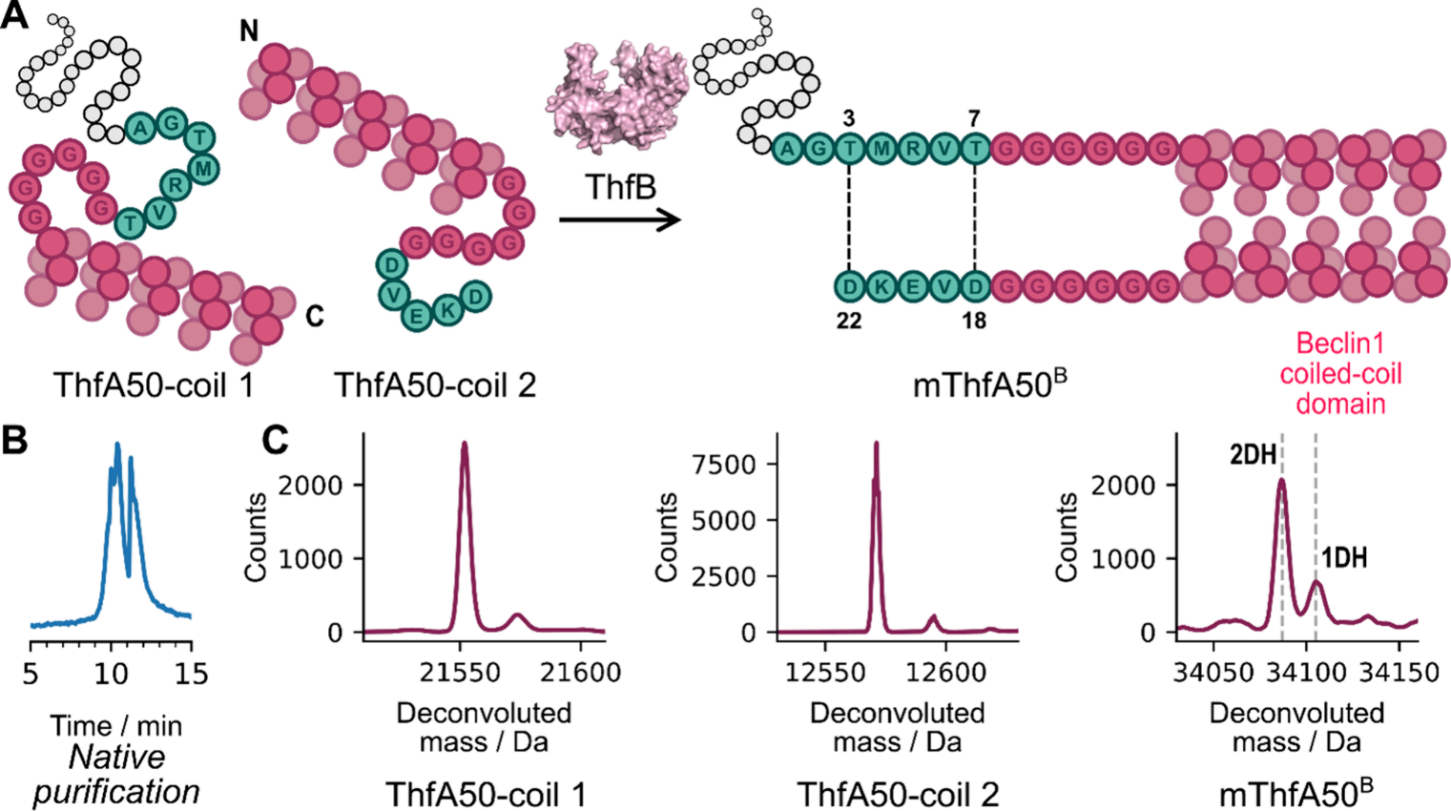
ThfB can cross-link two proteins. A: Schematic of ThfB mediated protein–protein coupling, with the native N-terminal leader (gray), core peptide (green) and the linker sequence and Beclin1 coil domain (pink). B: Total ion chromatogram from the native purification of mThfA50^B^ using Ni-affinity chromatography. The first peak includes ThfA50-coil 1, ThfA50-coil 2 and mThfA50^B^ while the second peak is ThfB (Figure S18). C: Deconvoluted mass spectra of ThfA50-coil 1, ThfA50-coil 2 and mThfA50^B^ (from left to right). 1DH and 2DH mean 1 and 2 dehydrations, respectively with each dehydration corresponding to an ester cross-link.

## Conclusion

Here we have shown that the ATP-grasp enzyme ThfB utilized in the biosynthesis of the natural product fuscimiditide has exceptional substrate tolerance allowing it to be used as a multipurpose biocatalyst for peptide and protein cyclization. Polypeptide sequences as short as 4 aa (construct ThfA4, Figure 3) and as long as 258 aa (construct ThfA49, Figure 7) can be effectively cyclized via the addition of short N- and C-terminal tags to the sequence of interest as well as a leader sequence for substrate recognition. While a wealth of synthetic strategies for cyclic peptides have been developed,^35,36^ our approach is carried out recombinantly, with only a protease cleavage step carried out *in vitro* to remove the leader peptide and furnish the final cyclic peptide. Several other methods exist for the cyclization of recombinant peptides or proteins such as the use of split inteins,^37^ the SpyTag-SpyCatcher system,^38,39^ sortase,^40^ and asparagine endopeptidases (AEPs) from plants such as butelase 1 ^41,42^ and OaAEP1.^43^ Of these methods, our approach is perhaps most similar to cyclization by butelase and other AEPs in that only a few additional amino acids are inserted into the protein of interest to allow for cyclization. A potential drawback of AEPs is that the cyclization reaction is usually carried out *in vitro* with a purified protein of interest and enzyme, though a recent report has shown that a variant of OaAEP1 can be expressed recombinantly in *E. coli* along with the protein of interest to afford cyclized protein of interest.^44^

The promiscuity of ThfB in accepting a wide variety of loop sizes also suggests future applications in epitope grafting into the loop of ThfA, similar to studies carried out on other RiPPs such as lasso peptides.^45^ The broad substrate tolerance of ThfB also allows for the construction of large, randomized libraries of cyclic peptides based on the ThfA scaffold. Screens on these libraries could be carried out in *E. coli* to identify inhibitors of protein–protein interactions or other undruggable targets.^46–48^ Alternatively, since ThfB also functions robustly *in vitro*,^14^ it could be used to cyclize libraries of peptides generated by mRNA display^49^ for downstream high-throughput screening applications. ThfB natively installs ester linkages on ThfA, which may be considered a liability in some applications due to the relative lability of esters. However, we have previously shown that a thioester-linked ThfA can be converted via an S–N acyl shift into a peptide with a near-native head-to-tail amide linkage.^18^

While the focus of this work has been on engineering applications of the ThfA/ThfB system, our results here also teach us something about the mechanism of ThfB. An AlphaFold 3 (AF3) model of the ThfA/ThfB complex does not accurately reflect the true interaction between these proteins since neither of the Asp residues that are phosphorylated by the ATP-grasp enzyme are near the active site (Figure S19). The pLDDT of the top AF3 model (Figure S20) shows that AF3 has low confidence in the prediction of the ThfA substrate. In the absence of insights from an AF3 model, we can infer from our experiments that the active site of ThfB does not include a well-formed pocket in which the ThfA loop docks. Instead, since ThfA can tolerate vastly different loop sizes and sequences, this part of the ThfA substrate is almost certainly solvent-exposed when bound to ThfB. Moreover, the fact that we observe some non-native ester linkages formed in constructs such as ThfA9 (Figure 3) suggests that the active site of ThfB does not precisely bind the N- and C-terminal portions of the stem of ThfA. Instead, ThfB generates acyl phosphates on Asp side chains that then must “find” their nucleophilic reaction partners. Overall, this work demonstrates how curiosity-driven natural products research can lead to discoveries and new tools in peripheral fields such as protein engineering.

## Supplementary Material





## Notes

Detailed materials and methods, additional mass spectrometry data, AlphaFold 3 models (PDF)
Transparent Peer Review report available (PDF)
