# Supplementary material for: Peptide and Protein Cyclization by a Promiscuous Graspetide Synthetase
Source: ACS Cent Sci. 2025 Jun 9;11(7):1111–21. doi: 10.1021/acscentsci.5c00408 (PMC12291112; doi:10.1021/acscentsci.5c00408)
Supplement: Supplementary file 1 [file oc5c00408_si_001.pdf]

Supporting Information

Peptide and Protein Cyclization by a Promiscuous Graspetide Synthetase

Brian Choi[†], Toby G. Johnson[†], Arthur Acuña[†], Hader E. Elashal[†], and A. James Link[†,‡,§,*]

[†]Department of Chemical and Biological Engineering, Princeton University, Princeton, NJ 08544, United States
[‡]Department of Chemistry, Princeton University, Princeton, NJ 08544, United States
[§]Department of Molecular Biology, Princeton University, Princeton, NJ 08544, United States
*Corresponding Author: ajlink@princeton.edu

Table of Contents

Methods...S2
Cloning...S3
Protein Expression and Purification...S4
Liquid Chromatography–Mass Spectrometry (LC-MS)...S4
Trypsin Digestion...S5
High-Pressure Liquid Chromatography (HPLC)...S5
Fluorescence Measurements...S5
Ester-Selective Hydrazinolysis...S6
Supplementary Figures...S7
Supplementary Tables...S27
References for Supplemental Information...S45

Methods

Accession numbers and sequences of proteins

ThfA (WP_011292231.1)
MSTAVTDAFPLGRDENRNDQVTEWRPFGMRYGVQPTPIPVPLSDTKYDPDQQVLVVA
DGQPCAKIERAGTMRVTYPDGQKPGQSDVEKD

ThfB (WP_193587235.1)

MTVLILTNPFDITADDVILRLTEHGVPVVRLDPADFPQQVVLHSEIGGNGWTGTLTTPH
RILDLSTVTGIWYRRPRKFRLPAQMSQAEYEFAATEARRGFGGIINSLTGWINHPSAIG
RAEYKPYQLHHAVQAGLNVPRTLITNDPKQAKGWCARVGDVVYKPLSAPSWLENGDT
YVVFTTPITPDQWGDPAIGRTAHMFQQRLDKEFEVRLTMVDGKAFPAAIHAHSDAARI
DWRSDYDALTYSIPTVPQRVLTGARDLLRRLHLRYAALDFIVSPDGRWHFLEVNPNGQ
YGWIEEHTGQPISDAIADALTRKEN

mRuby2

MVSKGEELIKENMRMKVVMEGSVNGHQFKCTGEGEGNPYMGTQTMRIKVIEGGPLPF
AFDILATSFMYGSRTFIKYPKGIPDFFKQSFPEGFTWERVTRYEDGGVVTVMQDTSLE
DGCLVYHVQVRGVNFPSNGPVMQKKTKGWEPNTEMMYPADGGLRGYTHMALKVDG
GGHLSCSFVTTYRSKKTVGNIKMPGIHAVDHRLERLEESDNEMFVVQREHAVAKFAGL
GGGMDELYK

sfGFP (avGFP S2R, S30R, Y39N, F64L, S65T, S72A, F99S, N105T, Y145F, M153T, V163A, I171V, A206V)
MRKGEELFTGVVPILVELDGDVNGHKFSVRGEGEGDATNGKLTLKFICTTGKLPVPWP
TLVTTLTYGVQCFARYPDHMKQHDFFKSAMPEGYVQERTISFKDDGTYKTRAEVKFE
GDTLVNRIELKGIDFKEDGNILGHKLEYNFNSHNVYITADKQKNGIKANFKIRHNVEDGS
VQLADHYQQNTPIGDGPVLLPDNHYLSTQSVLSKDPNEKRDHMVLLEFVTAAGITHGM
DELYK

mTurquoise2

MVSKGEELFTGVVPILVELDGDVNGHKFSVSGEGEGDATYGKLTLKFICTTGKLPVPW
PTLVTTLSWGVQCFARYPDHMKQHDFFKSAMPEGYVQERTIFFKDDGNYKTRAEVKF
EGDTLVNRIELKGIDFKEDGNILGHKLEYNYFSDNVYITADKQKNGIKANFKIRHNIEDG
GVQLADHYQQNTPIGDGPVLLPDNHYLSTQSKLSKDPNEKRDHMVLLEFVTAAGITLG
MDELYK

Beclin-1 from *Rattus norvegicus* amino acids 174-264 (UniProt: Q91XJ1)

DSEQLQRELKELALEEERLIQELEDVEKNRKVVAENLEKVQAEAERLDQEEAQYQREY
SEFKRQQLELDDELKSVENQMRYAQMQLDKLKK

Cloning

For molecular cloning, Golden Gate assembly was used as the primary method to construct ThfA variant plasmids, using a Golden Gate assembly-enabled pQE-80-based vector (pBC108) we previously reported.[1] This vector was generated from pQE-80L, in which the *Bsa*I recognition site in AmpR was ablated with a silence mutation (G239G, GGG → GGT) and the multicloning site (MCS) of pQE-80L was replaced with the GFP constitutive expression cassette flanked by *Bsa*I recognition sites that generate *Bam*HI and *Hind*III sticky ends upon digestion. The GFP constitutive expression cassette contains the coding sequence of sfGFP (avGFP S2R S30R Y39N F64L S65T S72A F99S N105T Y145F M153T V163A I171V A206V) under the control of PglpT promoter (Part: jtk2821; BBa_J72163 from iGEM Registry of Standard Biological Parts). The arginine residue of the His_6-tag was also mutated to serine to suppress the background methylation of the N-terminus in *E. coli.*

Inserts were prepared by PCR amplification using the reagents (dNTP, Q5 High Fidelity DNA polymerase) purchased from New England Biolabs (NEB). As a template for PCR amplification, the plasmid encoding the *thfA* gene in multicloning site 1 (MCS1) of the pRSF-duet vector was used. Oligonucleotide primers for PCR amplification were designed with overhangs of the primers encoding the designed amino acid substitutions and purchased from Integrated DNA Technologies (IDT). Amplified inserts were purified using Zymoclean™ Gel DNA Recovery Kit (Zymo Research) after gel electrophoresis. Short inserts (<61 bp) were prepared by oligonucleotide annealing using T4 polynucleotide kinase (NEB). Golden Gate assembly was carried out with *Bsa*I-HFv2 and T4 DNA ligase (NEB). To propagate the assembled plasmids, *E. coli* XL1-Blue (Stratagene) cells were transformed using Mix & Go! Transformation Kit (Zymo Research) and grown in lysogeny broth (LB) medium (5 g L^{-1} yeast extract (IBI Scientific), 10 g L^{-1} tryptone (IBI Scientific), 10 g L^{-1} NaCl, supplemented with 50 µg mL^{-1} kanamycin or 100 µg mL^{-1} ampicillin, as needed for selection). Plasmids were recovered and purified using QIAprep Spin Miniprep Kit (QIAGEN). The DNA sequences of the inserts of all constructed plasmids were confirmed by Sanger Sequencing (GENEWIZ/Azenta Life Sciences). The sequences of all oligonucleotides used in this study are in Table S10 and all plasmid constructs are in Table S11.

Cloning of pBC401 expressing ThfA50-coil 1 and ThfA50-coil 2

The coiled-coil homodimer of the protein Beclin was used as a starting point for a construct to test whether ThfB could crosslink a protein complex. A DNA fragment encoding a 6 Gly linker, followed by the amino acids GPGS, amino acids 174-264 of *Rattus norvegicus* Beclin, and finally KDVEKD was ordered from Twist Biosciences. This region of Beclin assembles into a homodimeric antiparallel coiled-coil. To generate the His_6-ThfA leader-core(1-7)-Gly_6-Beclin construct (referred to as ThfA50-coil 1 in the main text), the ThfA leader-core(1-7) fragment was amplified from a pRSF-Duet vector harboring His_6-ThfA with primers oBC576 and oBC577 (Table S10). The Gly_6-Beclin fragment was amplified using oBC578 and oBC579 from the linear Beclin DNA fragment

template. The full gene was assembled by overlap PCR of these two fragments. This gene was cloned into MCS1 of pRSF-Duet by digestion with *Bam*HI and *Hin*dIII to give plasmid pBC379. To generate the Beclin-KDEVKD construct (referred to as ThfA50-coil 2 in the main text), primers oBC618 and oBC619 were used with pBC379 as a template to generate the desired PCR product. The resulting product was digested with *Nde*I and *Xho*I and cloned into MCS2 of pBC379 to furnish pBC401. This plasmid is capable of producing the two proteins ThfA50-coil 1 and ThfA50-coil 2 upon IPTG induction.

Protein Expression and Purification

A ThfA variant plasmid and a plasmid encoding ThfB were transformed into electrocompetent *E. coli* BL21 (DE3) Δ*slyD* cells and plated onto lysogeny broth (LB) agar plates containing 100 mg L^{-1} ampicillin and 50 mg L^{-1} kanamycin. Starter cultures were created by picking colonies into LB (5 mL, 100 mg L^{-1} ampicillin, 50 mg L^{-1} kanamycin) and grown overnight at 37 °C. These cells were subcultured into LB media (500 mL, 100 mg L^{-1} ampicillin, 50 mg L^{-1} kanamycin) at a starting optical density at 600 nm (OD_{600}) of 0.02 and grown at 37 °C. Upon reaching an OD_{600} of 0.5, cells were induced with 1 M IPTG (500 μL, final concentration 1 mM), and then left to shake at room temperature overnight (16-18 h). Cells were then centrifuged at 4000 *g* for 15 min at 4 °C before proceeding to denaturing purification.

For denaturing purification of ThfB-modified ThfA variants, the cell pellet (per 500 mL of culture) was resuspended in urea buffer (10 mL, 100 mM NaH_2PO_4, 10 mM Tris-base, 8 M urea, pH 8.0) and then frozen (-80 °C) and thawed in a water bath (room temperature). The cell lysate was centrifuged (at least 16,000 *g*) until clarified (usually 15 min). The clarified lysate was incubated with 1 mL Ni-NTA resin (Qiagen) at 4 °C for 1 h. This mixture was then passed through a gravity column, washed with urea buffer (10 mL, pH 6.4), then washed with urea buffer (10 mL, pH 5.9). Protein was then eluted with urea buffer (pH 4.5), collecting 8×1 mL fractions.

Liquid Chromatography–Mass Spectrometry (LC-MS)

LC-MS analysis was performed using an Agilent 6530 QTOF connected to an Agilent 1260 Infinity II LC system. Mass spectra were acquired using electrospray ionization (ESI) with the instrument in positive ion mode. The mobile phase A was water with 0.1% formic acid, and mobile phase B was acetonitrile with 0.1% formic acid. Intact proteins were run on a Xbridge Protein BEH C4 column (2.1 mm × 50 mm, 3.5 μM particle size, Waters) with the following gradient used: 10% Solvent B, 0-2 min; 10-50% B, 1-15 min; 50-90% B, 15-20 min; 90% B, 20-30 min, flowing at 0.5 mL min^{-1}. Deconvoluted mass spectra of the analyte (ThfA variant) were acquired using the Agilent MassHunter Bioconfirm software.

Either a crude trypsin digestion mixture or an HPLC-purified core peptide (see below) was run on a Zorbax 300SB-C18 column (2.1 mm x 50 mm, 3.5 µm particle size) with the following gradient used: 5% B from 0-1 min; 5-45% B from 1-20 min; 45-90% B from 20-25 min; 90% B from 25-30 min, flowing at 0.5 mL min^{-1}. The resulting LC traces and mass spectra were extracted and analyzed using the Agilent MassHunter Qualitative Analysis software for manual inspection. The Agilent MassHunter Bioconfirm software was used to automate the identification of the tryptic fragments.

For LC-MS/MS analysis, collision-induced dissociation (CID) was performed with 1.3 m/z isolation width and a defined collision energy (based on the formula, V = 0.036 × (m/z) – 4.8, unless specified otherwise). MS/MS spectra were extracted from the Agilent MassHunter Qualitative Analysis software and analyzed manually using mMass.[4] Fragment ions were identified and assigned based on the m/z error of the monoisotopic peak, ion intensity, and the isotopic distribution.

Trypsin Digestion

For variants to be digested, all elution fractions were combined, concentrated and buffer-exchanged into phosphate buffer (100 mM NaH_2PO_4, pH 7.0) using an Amicon Ultra-4 10 kDa cut-off centrifugal filter (Millipore). The resulting protein sample was digested with Sequencing grade Modified Trypsin (Promega) in ammonium bicarbonate (50 mM, pH~8 after preparation) at 37 °C for 15-30 min. A 1:200 (w/w) trypsin:protein ratio was used. Addition of formic acid (final concentration 1% v/v) quenched the digestion for LC-MS analysis.

High-Pressure Liquid Chromatography (HPLC)

The core peptide of a ThfB-modified ThfA variant was obtained by trypsin digestion (using 1:200 mass ratio of trypsin to substrate) and purified by semi-preparative reverse-phase HPLC using the Agilent 1200 series instrument, equipped with the Zorbax 300SB-C18 (9.4 mm x 250 mm, 5 µm) column and UV detector wavelength set at 215 nm. The following gradients using a binary mixture of solvents (Solvent A: ultrapure water with 0.1% trifluoroacetic acid (TFA); Solvent B: acetonitrile with 0.1% TFA) were used for chromatography: 10% B from 0-1 min; 10-50% B from 1-20 min; 50-90% B from 20-25 min; 90% B from 25-30 min; 90-10% B from 30-32 min, flowing at 4.0 mL min^{-1}. The desired peak was collected, and analyzed by LC-MS. The purified peptide was frozen (-80 °C) and lyophilized (Labconco FreeZone Freeze Dry System) to give a white solid which was dissolved in deionized water.

Fluorescence Measurements

A protein construct containing mRuby2 was concentrated and buffer-exchanged into a Tris buffered solution (50 mM Tris-HCl, 200 mM NaCl, pH 8.0) using an Amicon Ultra-4 10 kDa (or 30 kDa, as appropriate) cut-off centrifugal filter. The protein was further purified by size exclusion chromatography using an ÄKTApurifier chromatography system (GE), fitted with Superdex 200 Increase 10/300 GL column and eluted with the Tris buffered solution (50 mM Tris-HCl, 200 mM NaCl, pH 8.0) at 1 mL min^{-1} flow rate. Fluorescence was measured in BioTek Synergy 4 plate reader with excitation wavelength at 559 nm and emission wavelength at 600 nm.

Ester-Selective Hydrazinolysis

26 μL of either an HPLC-purified peptide (at variable concentration, 0.1-3.0 mg mL^{-1}) or crude product from tryptic digestions was mixed with the hydrazinolysis solution (10 μL of 35 wt% hydrazine solution in water (Sigma-Aldrich), 15 μL of 1X PBS solution (pH 5.0, Cold Spring Harbor),[5] 1.0 μL 6 M HCl). The mixture was heated at 55 °C for 45 min and then subjected to LC-MS(/MS) analysis.

Supplementary Figures

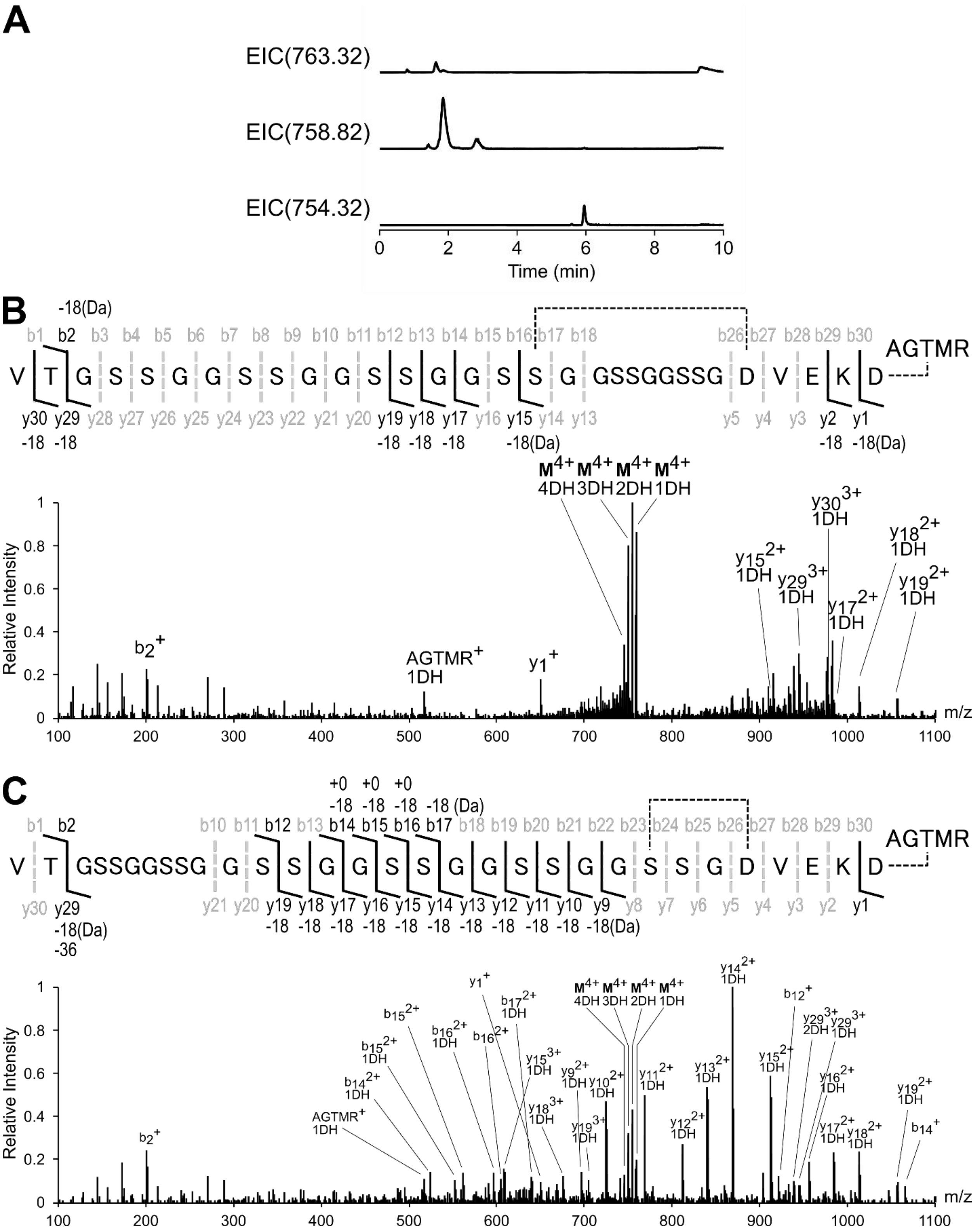

Figure S1. (Continued next page)

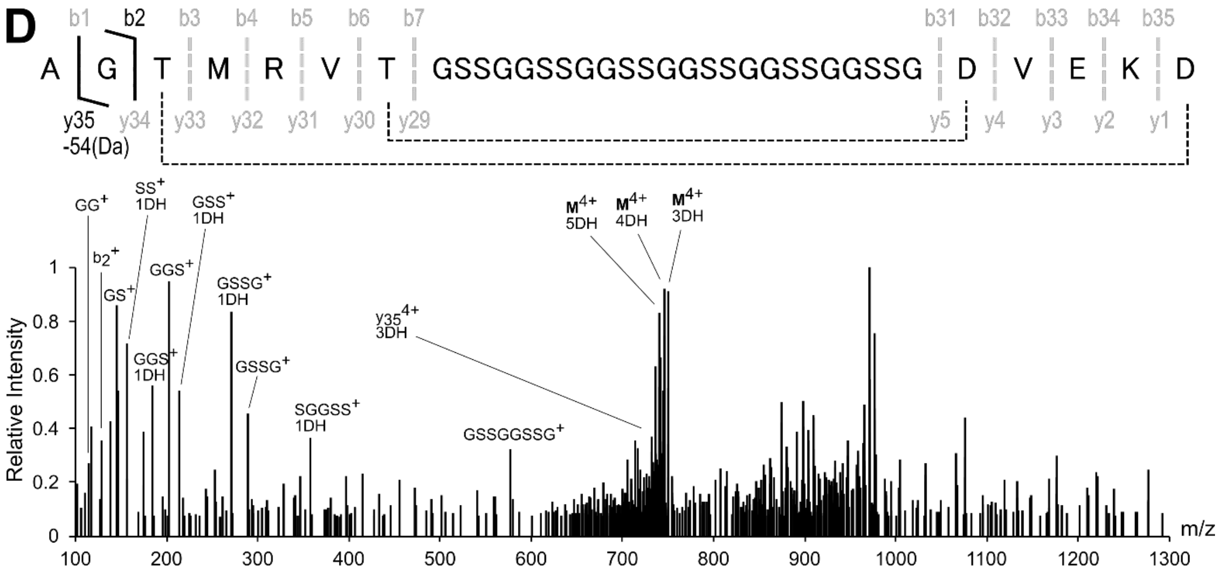

Figure S1. Mass spectrometry analysis of trypsin digested mThfA9^{B}. (A) Extracted ion chromatograms of unmodified, net singly dehydrated, and net doubly dehydrated core peptide fragments (top to bottom). Masses in parentheses correspond to quadruply-charged ions. (B) MS/MS analysis of the net singly dehydrated core peptide fragment major peak (retention time 2 min). (C) MS/MS analysis of the singly modified core peptide fragment minor peak (retention time 3 min). (D) MS/MS analysis of the net doubly dehydrated core peptide fragment. All identified ions from the MS/MS spectra in panels B, C, and D are listed in Table S1, S2, and S3, respectively.

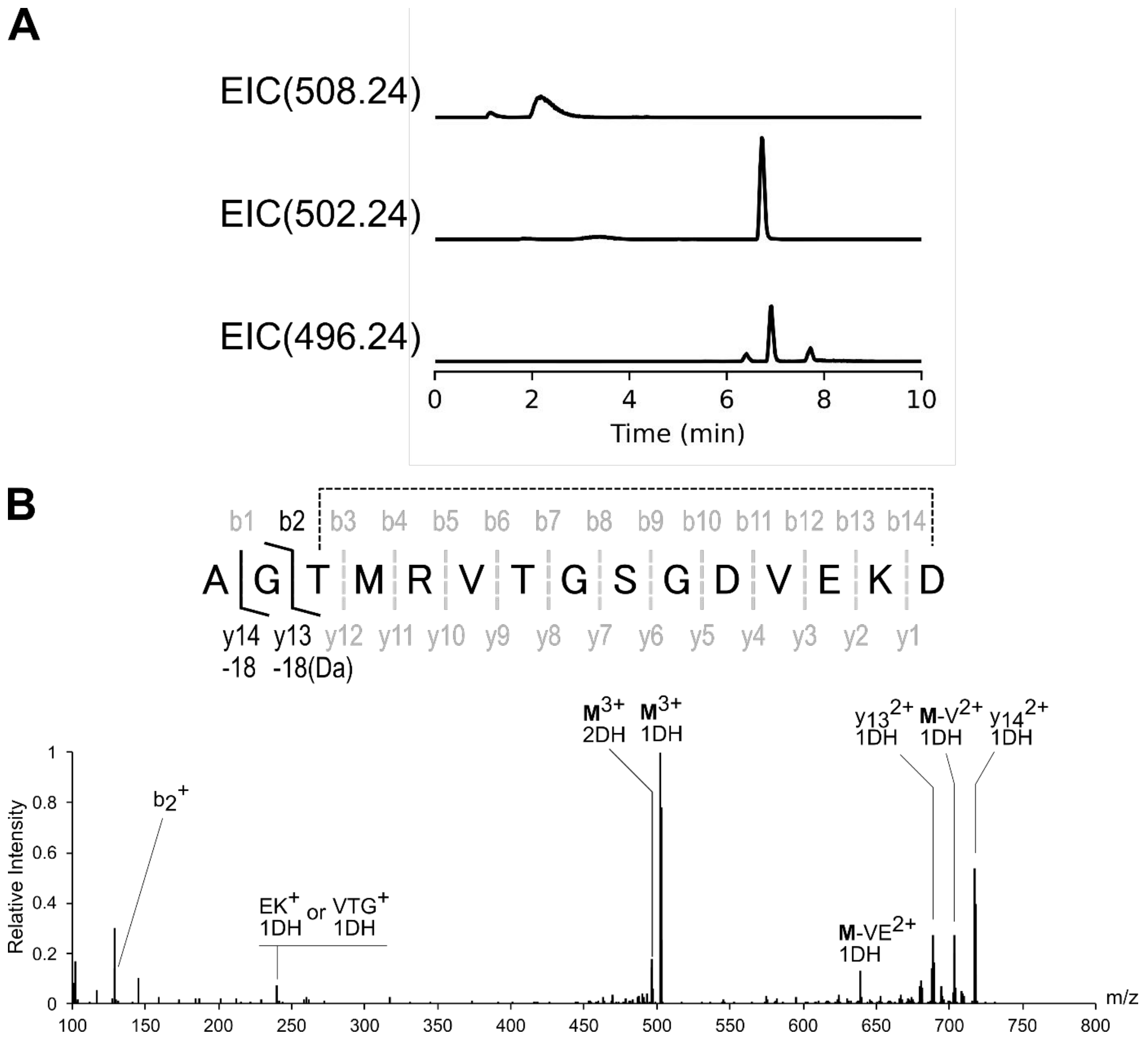

Figure S2. Mass spectrometry analysis of trypsin digested mThfA3^{B}. (A) Extracted ion chromatograms of unmodified, net singly dehydrated, and net doubly dehydrated core peptide fragments (top to bottom). Masses in parentheses correspond to triply charged ions. (B) MS/MS analysis of the net singly dehydrated core peptide fragment. All identified MS/MS ions are listed in Table S4. The **M**-V and **M**-VE labels correspond to ions arising from cleavage of residues internal to the macrocycle.

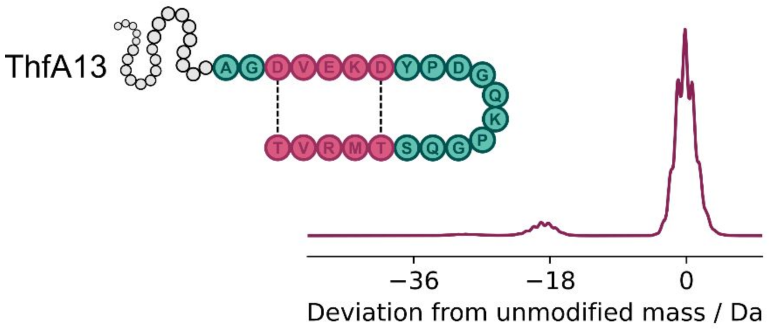

Figure S3. Mass spectrometry analysis of ThfB-modified ThfA13 with the N-terminal sequence (TMRVT) swapped with the C-terminal sequence (DVEKD) in the stem macrocycle. (Left) Cartoon showing the sequence and intended ester connectivity of the variant. (Right) Deconvoluted mass spectra of mThfA13^{B} as a whole protein. ThfA13 exhibited no modification by ThfB, remaining as a linear peptide.

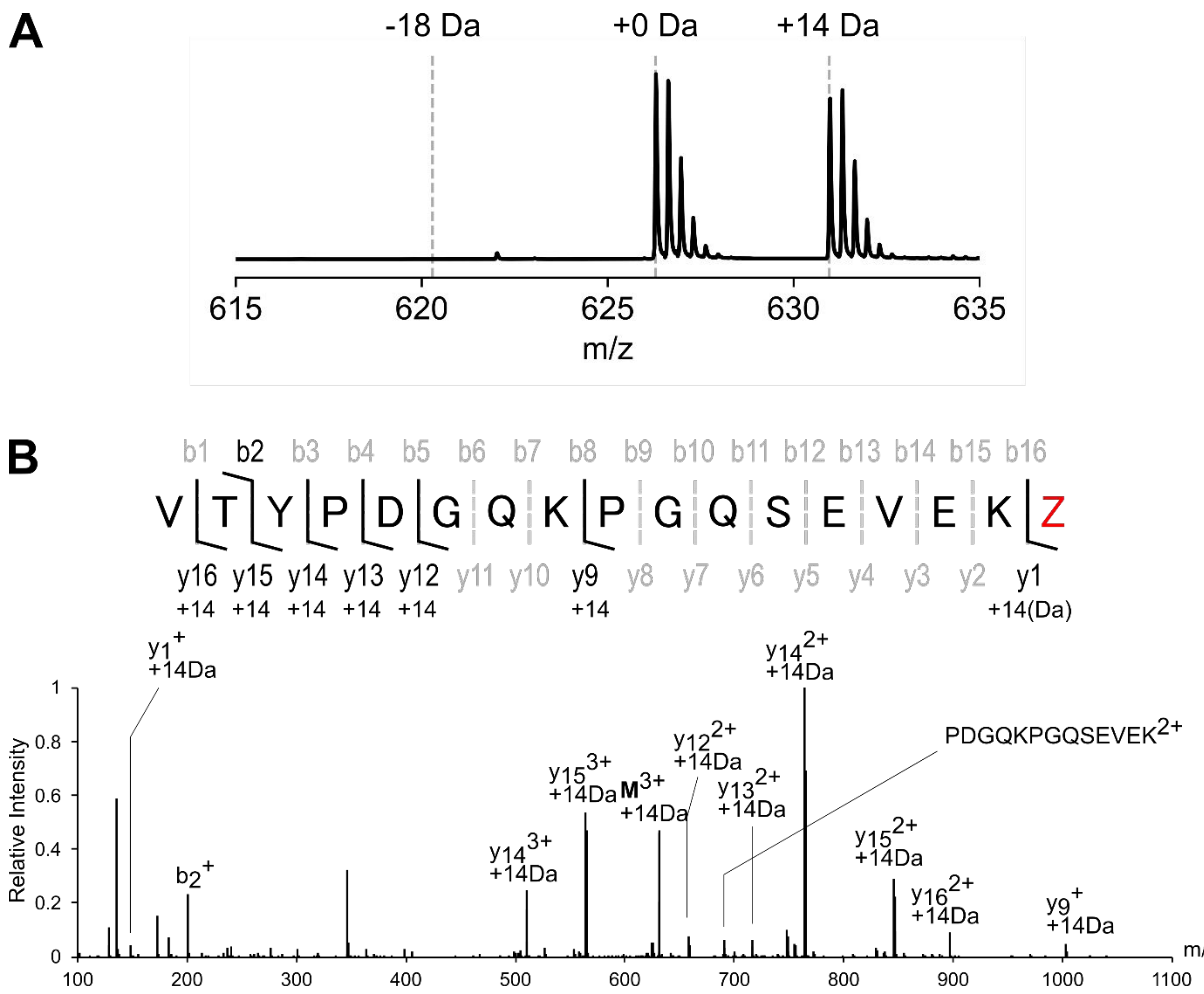

Figure S4. Mass spectrometry analysis after hydrazinolysis of tryptic digest of mThfA16[B]. (A) Mass spectrum showing the z=3 ions for unmodified (+0 Da) and acyl hydrazide (+14 Da) tagged core peptide as well as a marker for the mass of the dehydrated peptide (-18 Da). (B) MS/MS analysis of the acyl hydrazide tagged core peptide. All identified ions are listed in Table S5.

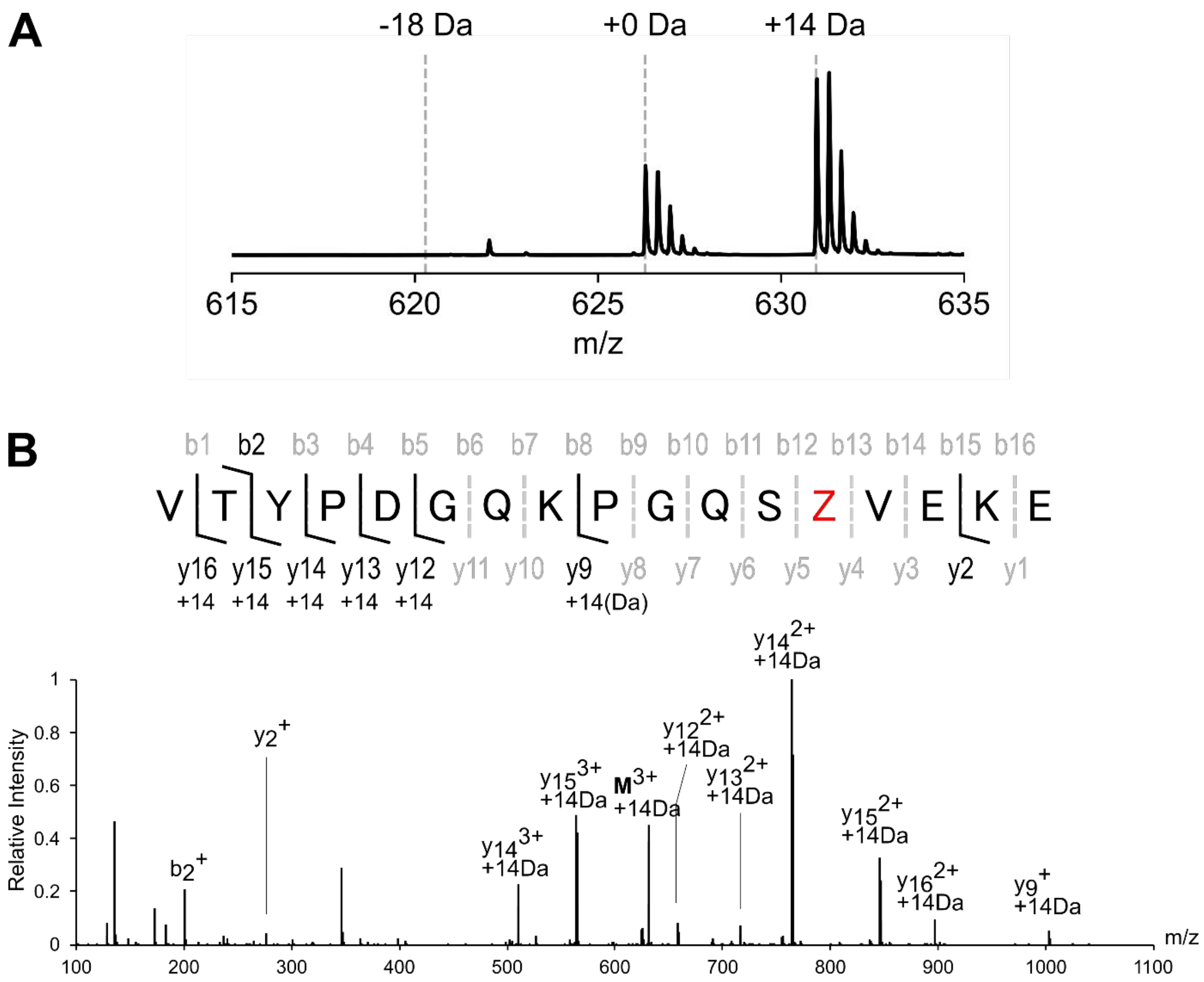

Figure S5. Mass spectrometry analysis after hydrazinolysis of tryptic digest of mThfA17[B]. (A) Mass spectrum showing the z=3 ions for unmodified (+0 Da) and acyl hydrazide (+14 Da) tagged core peptide as well as a marker for the mass of the dehydrated peptide (-18 Da). (B) MS/MS analysis of the acyl hydrazide tagged core peptide. All identified ions are listed in Table S6.

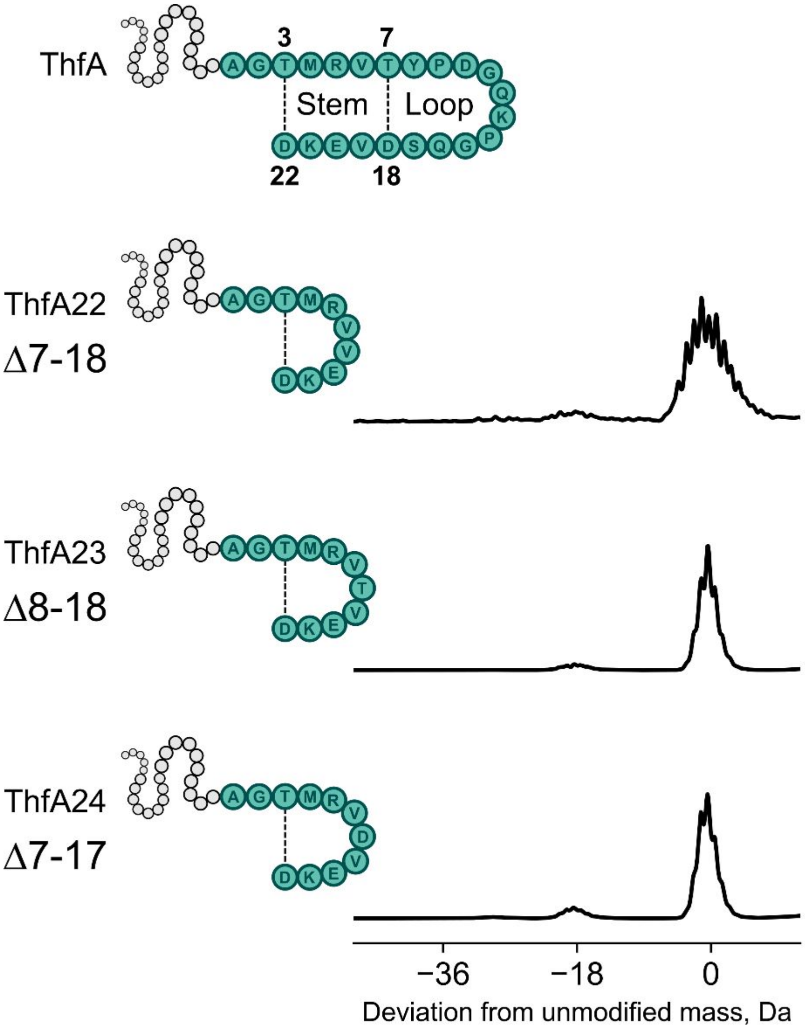

Figure S6. Mass spectrometry analysis of the ThfB-modified variants ThfA22-24 with the loop and Thr7 and/or Asp18 deleted. (Left) Cartoon showing the sequence and intended ester connectivity of the variants. The structure of native ThfA is shown at the top as a reference. (Right) Deconvoluted mass spectra of the ThfB-modified variants ThfA22-24 (top to bottom) as whole proteins. All variants exhibited no modification by ThfB, remaining as linear peptides.

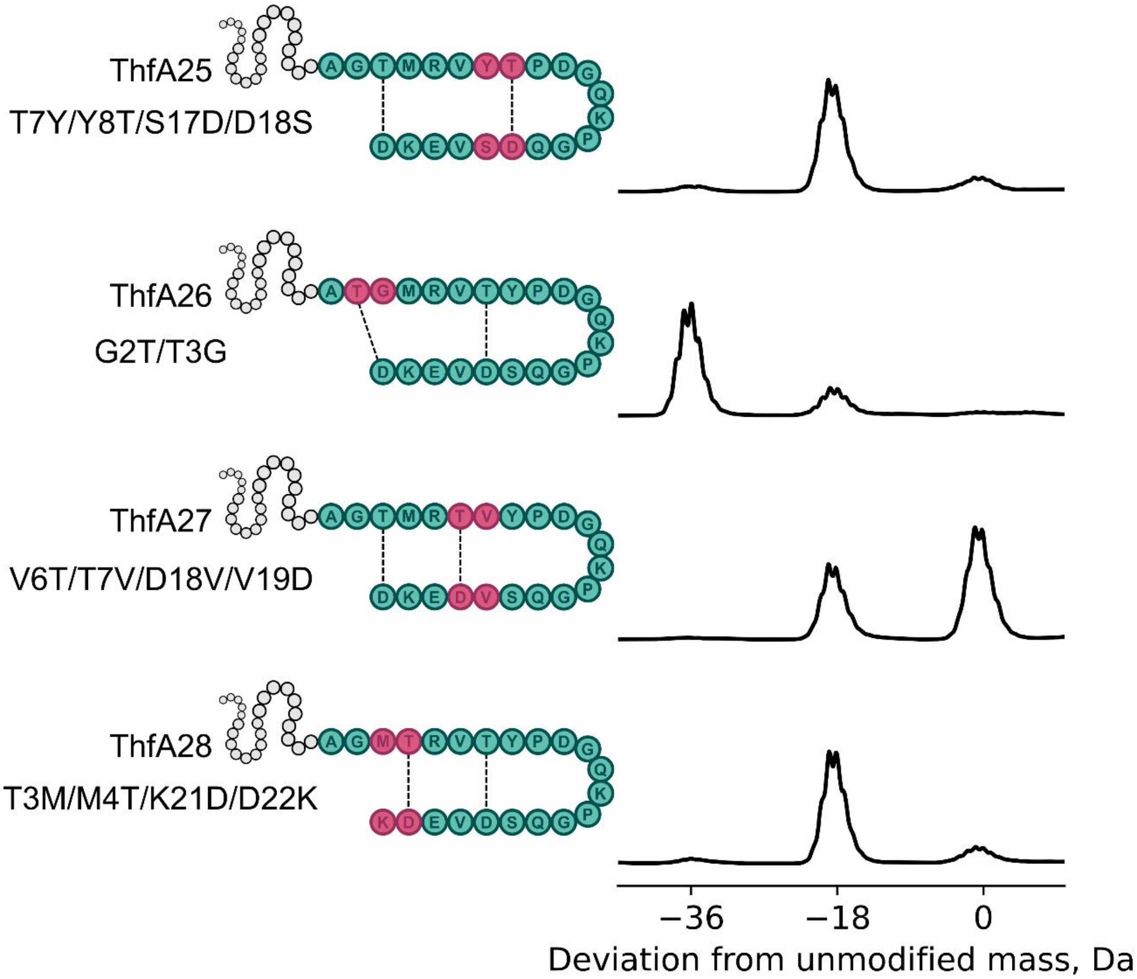

Figure S7. Mass spectrometry analysis of the ThfB-modified variants ThfA25-28 which altered the size of the stem macrocycle by one or two residues. (Left) Cartoon showing the sequence and intended ester connectivity of the variants. (Right) Deconvoluted mass spectra of the ThfB-modified variants ThfA25-28 (top to bottom) as whole proteins. In general the variants were poorly tolerated by ThfB and exhibited reduced modification efficiency, other than ThfA26 which was mainly doubly dehydrated. The successful two-fold dehydration of ThfA26 compared to the other variants tested here suggests that keeping the Asp residues D18 and D22 in their native positions may improve esterification efficiency.

**ThfA**	AKIERAGTMRVTYPDGQKPG-QSDVEKD
***T. alba* putative graspetide precursor**	AKGDPTATFPPTYRDGQTPNTPADFEKDSDTEN
**ThfA29**	AKIERTGTMRVTYPDGQKPG-QSDVEKDSD

Figure S8. Sequence alignment of the C-terminal region of ThfA, *T. alba* putative graspetide precursor, and ThfA29. The green highlighted amino acids are identical matches between ThfA's ester-forming residues and the putative graspetide precursor from *T. alba*. The pink highlighted amino acids are the substitutions made in ThfA29 to mimic the third pair of ester-forming residues in the putative graspetide precursor from *T. alba*.

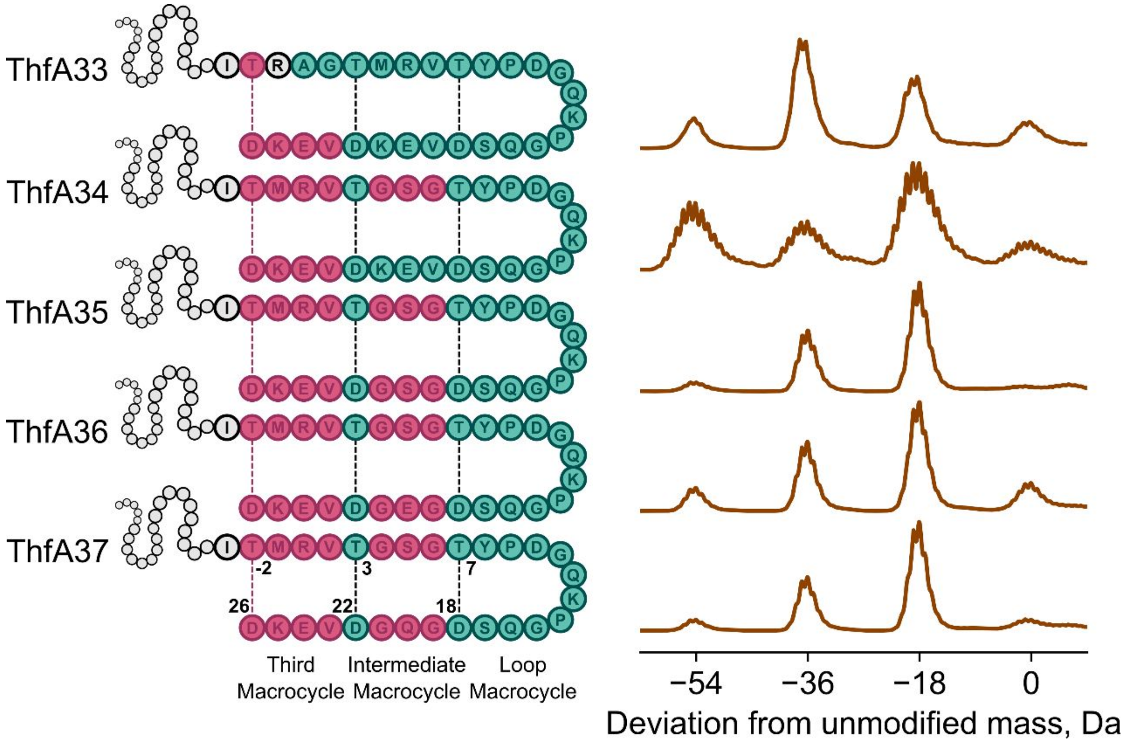

Figure S9. Mass spectrometry analysis of the ThfB-modified ThfA tricyclic variants ThfA33-37 with different interstitial amino acids in the two stem macrocycles. (Left) General structure of the tricyclic constructs with the native N-terminal leader (grey) and core peptide (green) with non-native amino acids shown in pink, with expected crosslinks depicted as dashed lines. (Right) Deconvoluted mass spectra of the ThfB-modified variants ThfA33-37 (top to bottom) as whole proteins.

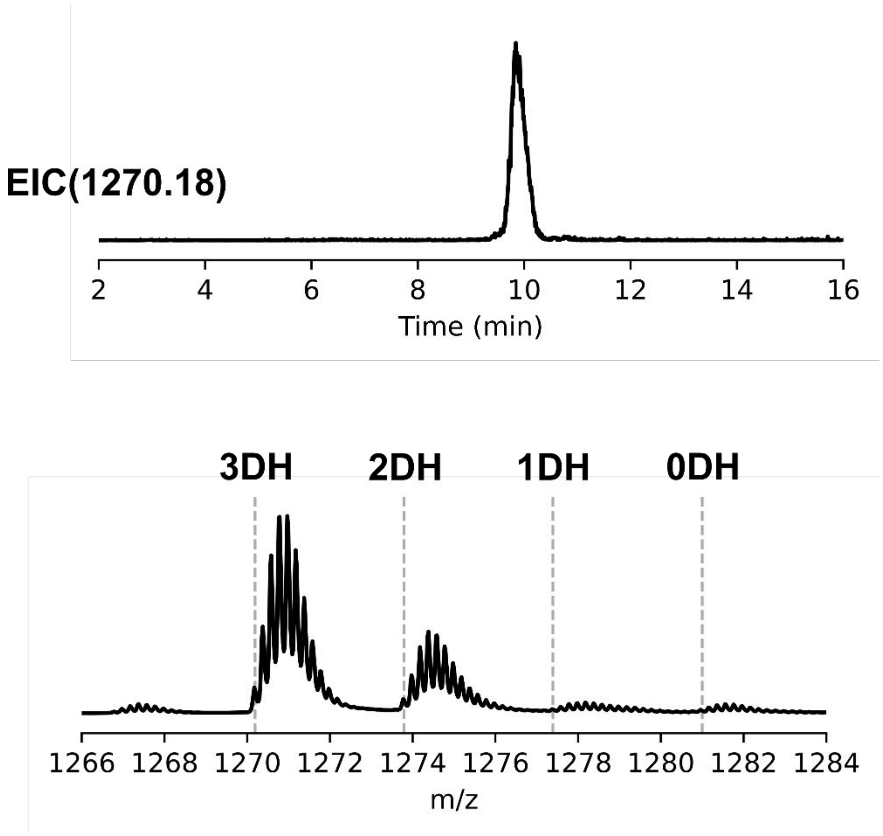

Figure S10. Mass spectrometry analysis of trypsin digested mThfA46^{B}. (Top) Extracted ion chromatogram of the 63 aa long, triply dehydrated core peptide obtained from trypsin digestion of mThfA46^{B}, with monoisotopic mass of 1270.18 and z=5, matching the expected mass of 6345.91 Da. (Bottom) Mass spectrum extracted from the peak shown above. The vertical dotted grey lines mark the expected monoisotopic masses for the triply, doubly, and singly dehydrated and linear 63 aa long tryptic core peptide fragment.

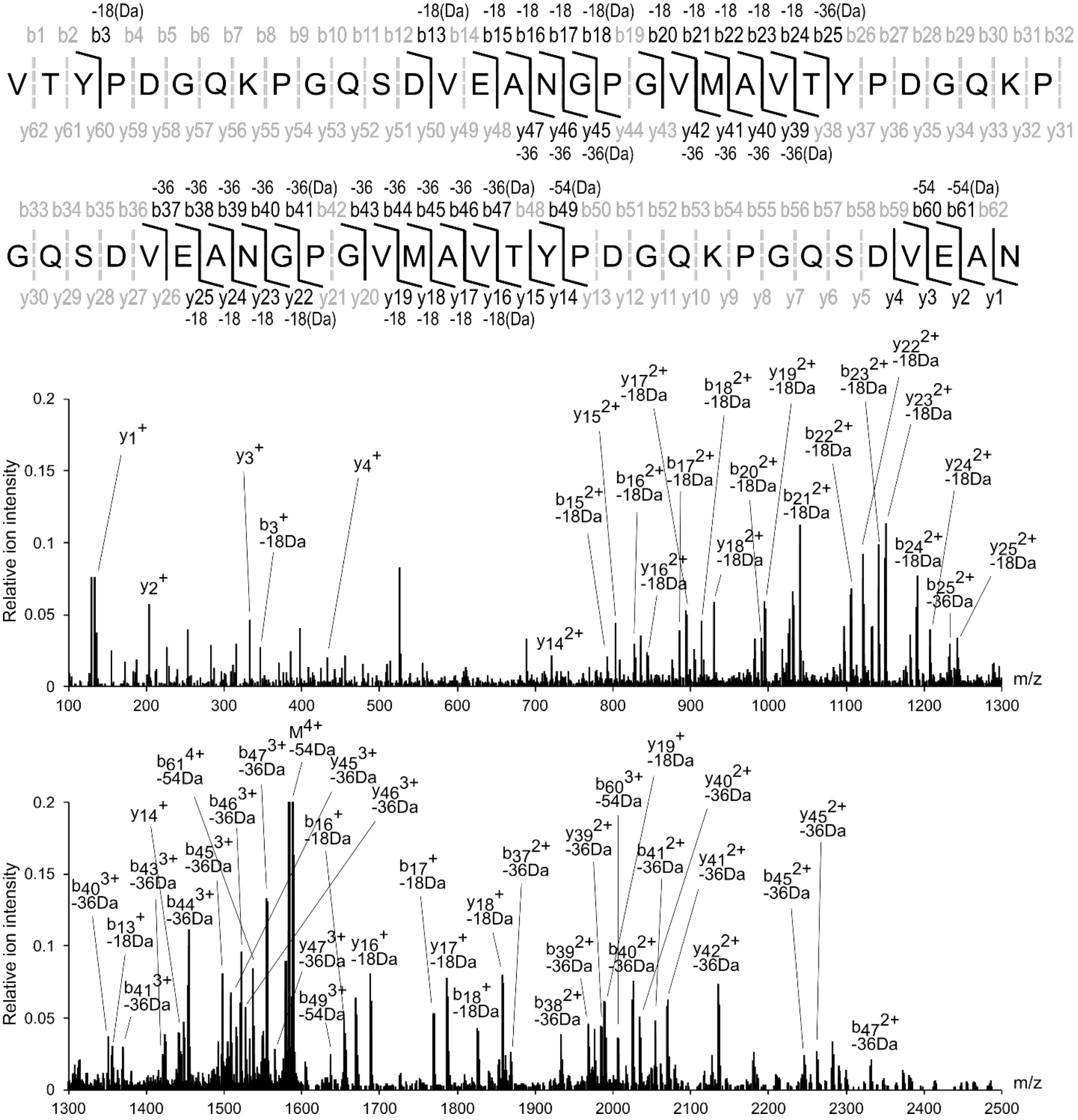

Figure S11. Tandem mass spectrometry analysis of the triply dehydrated core peptide of mThfA46[B], obtained from trypsin digestion. All identified ions are listed in Table S7.

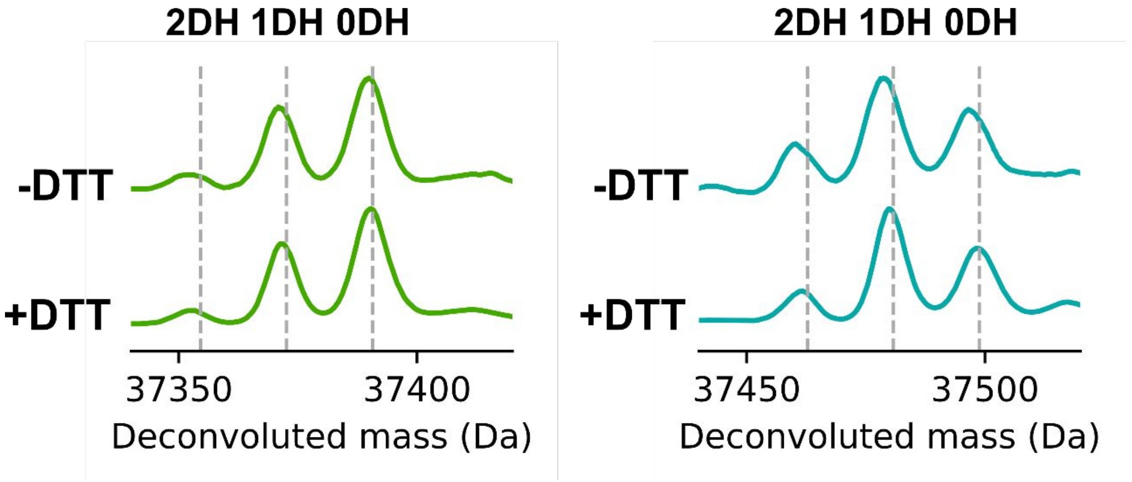

Figure S12. Deconvoluted mass spectra (Left) of mThfA48[B] with sfGFP-inserted and (Right) mThfA49[B] with mTurquoise2-inserted before and after DTT treatment. There is a +2 Da mass shift upon exposure to DTT, indicative for the reduction of a disulfide bond. Vertical lines for the different dehydrated products of the fully formed chromophores are shown for reference.

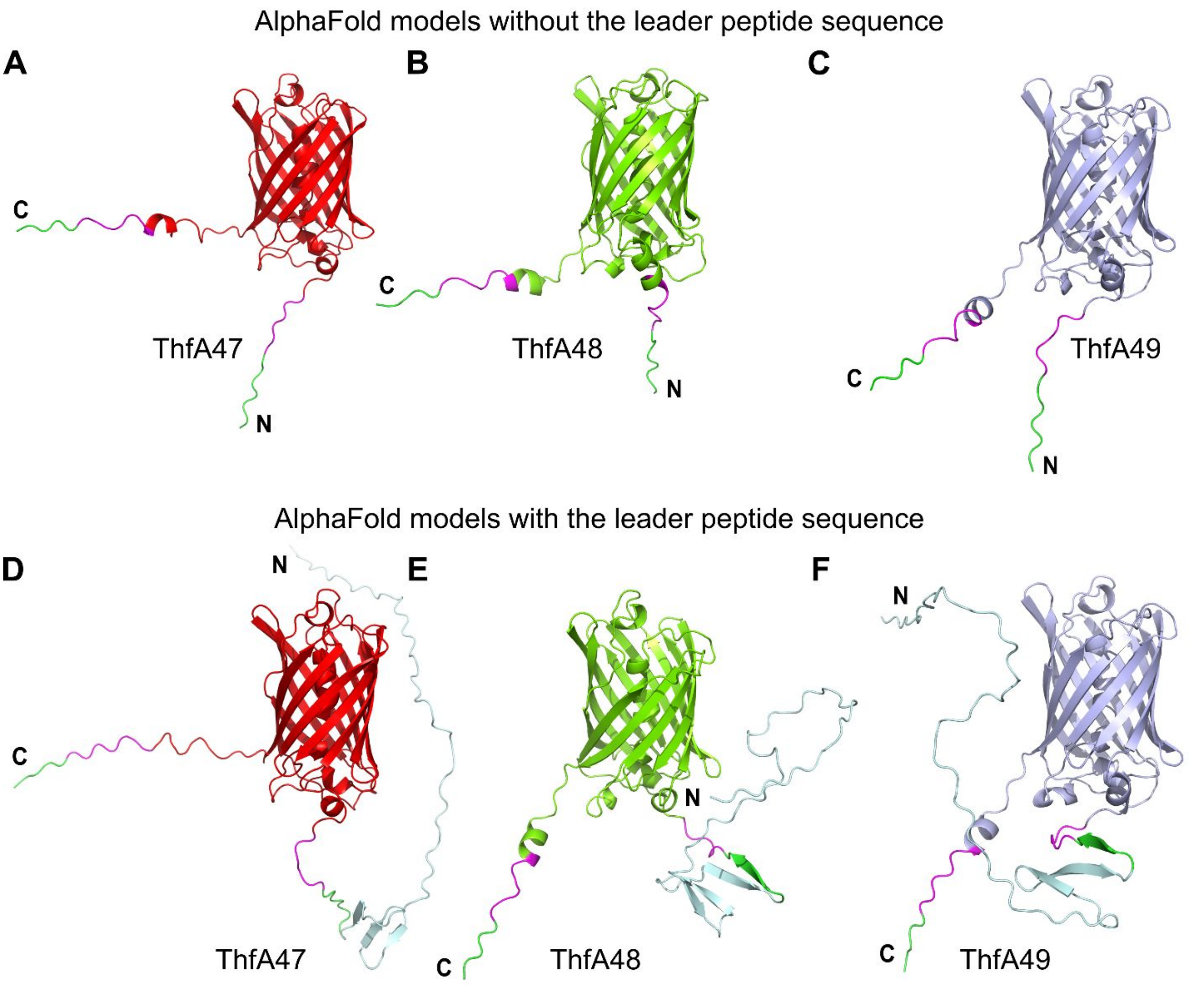

Figure S13. AlphaFold models of variants ThfA47-49 with fluorescent proteins inserted in the loop. The core peptide sequence is colored green, the linkers are magenta and the fluorescent proteins are color coded red (mRuby2), light green (sfGFP) and cyan (mTurquoise2). The N and C termini are labeled for each protein model. (A-C) The structures of the mRuby2 (ThfA47), sfGFP (ThfA48) and mTurquoise2 (ThfA49) loop variants are shown, respectively, without the leader sequence modeled. (D-F) The structures of the mRuby2 (ThfA47), sfGFP (ThfA48) and mTurquoise2 (ThfA49) loop variants are shown, respectively, with the leader sequence modeled. Note that the pLDDT values of the leader segments of all three constructs are low, suggesting low confidence in the predictions. Instead, these figures should be used to give the reader a sense of the size of the cyclization domains and the leader peptide.

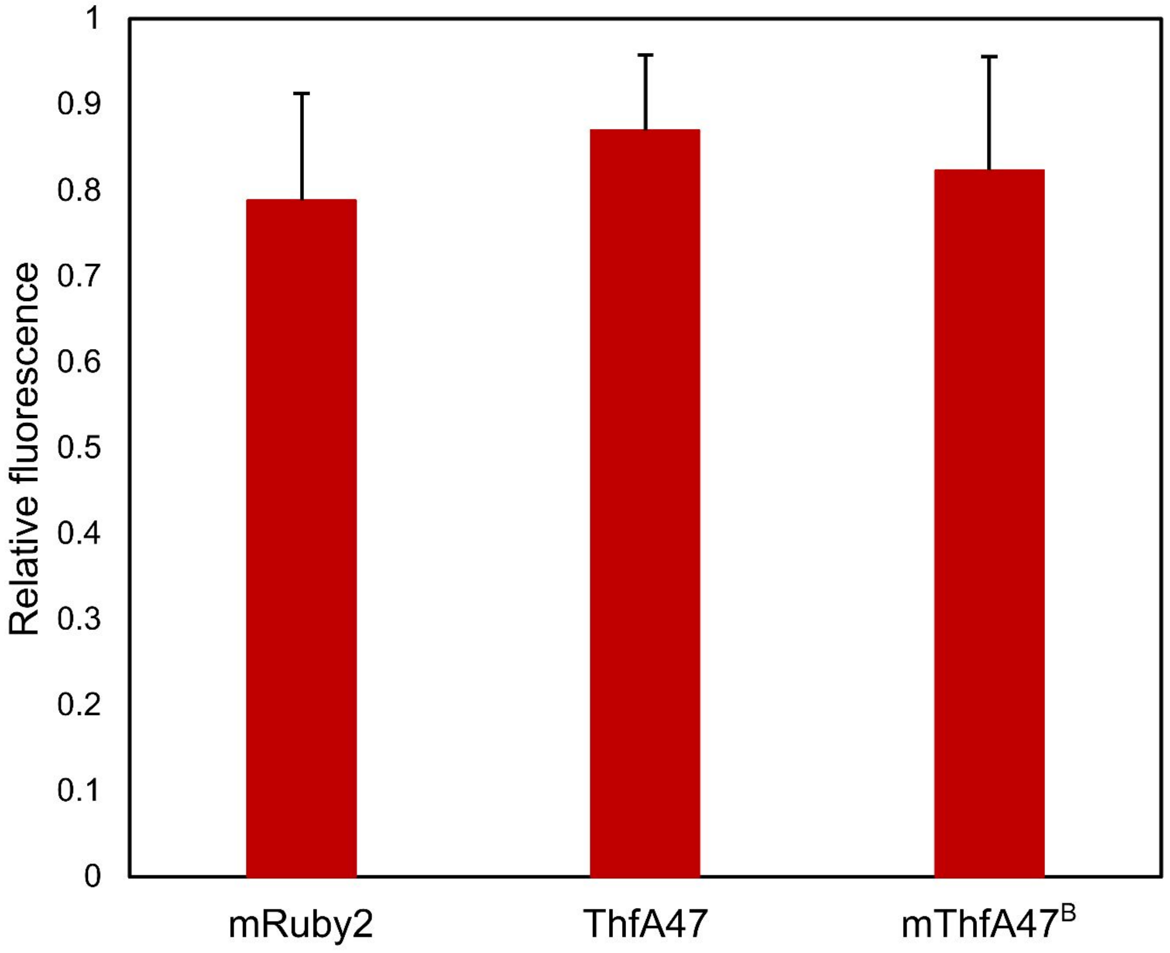

Figure S14. Fluorescence comparison between mRuby2, ThfA47 (with the mRuby2 sequence inserted in the loop of ThfA), and mThfA47^{B} (cyclized protein product). Relative fluorescence was calculated by dividing the measured fluorescence value by molar concentration and then normalizing by the highest calculated value. The height of each bar represents the average of 4 replicates, and the lengths of the error bars represent the standard deviations. The differences in relative fluorescence are not statistically significant.

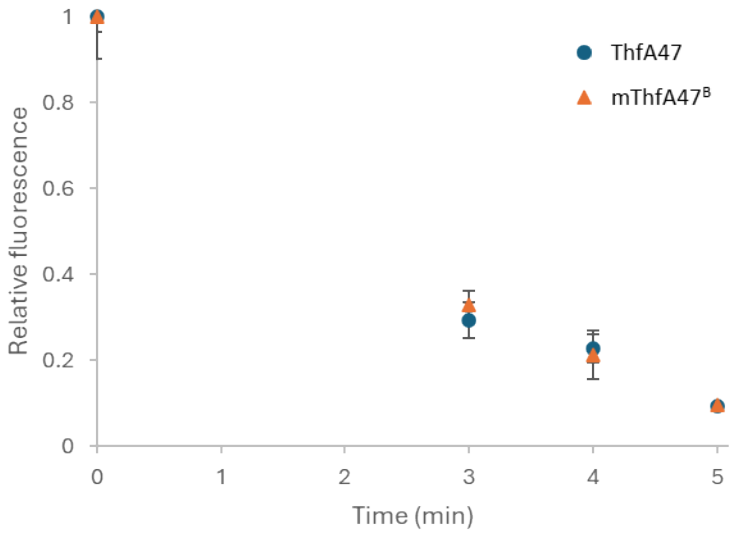

Figure S15. Fluorescence comparison between ThfA47 (with the mRuby2 sequence inserted in the loop of ThfA), and mThfA47^{B} (cyclized protein product) after heating (0-5 min, 90 °C). Relative fluorescence was calculated by dividing the measured fluorescence value by the initial fluorescence before heating. The height of each bar represents the average of 4 replicates, and the lengths of the error bars represent the standard deviations. The differences in relative fluorescence are not statistically significant.

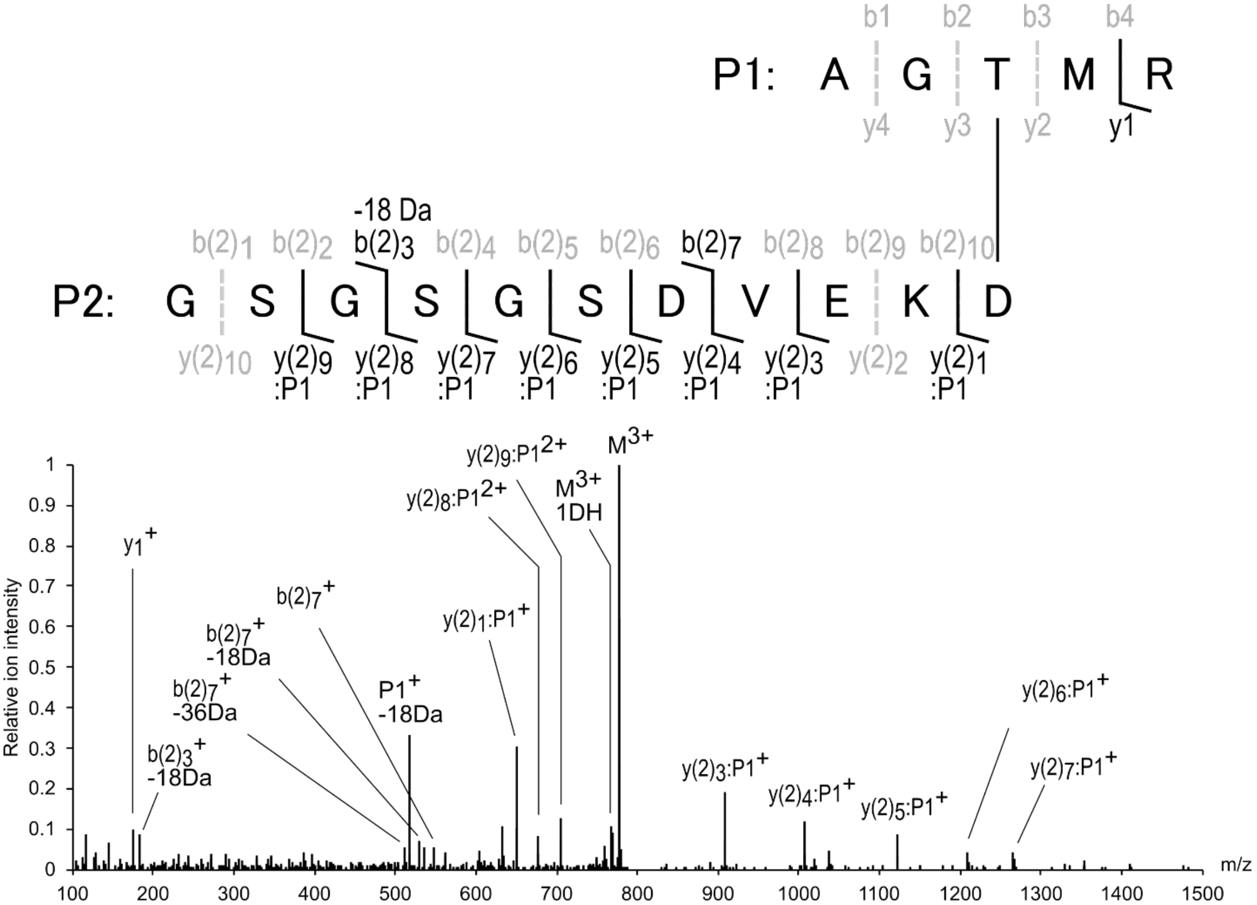

Figure S16. Tandem mass spectrometry analysis of the singly dehydrated tryptic fragment i (1552.67 Da) obtained from trypsin digestion of mThfA47[B]. All identified ions are listed in Table S8.

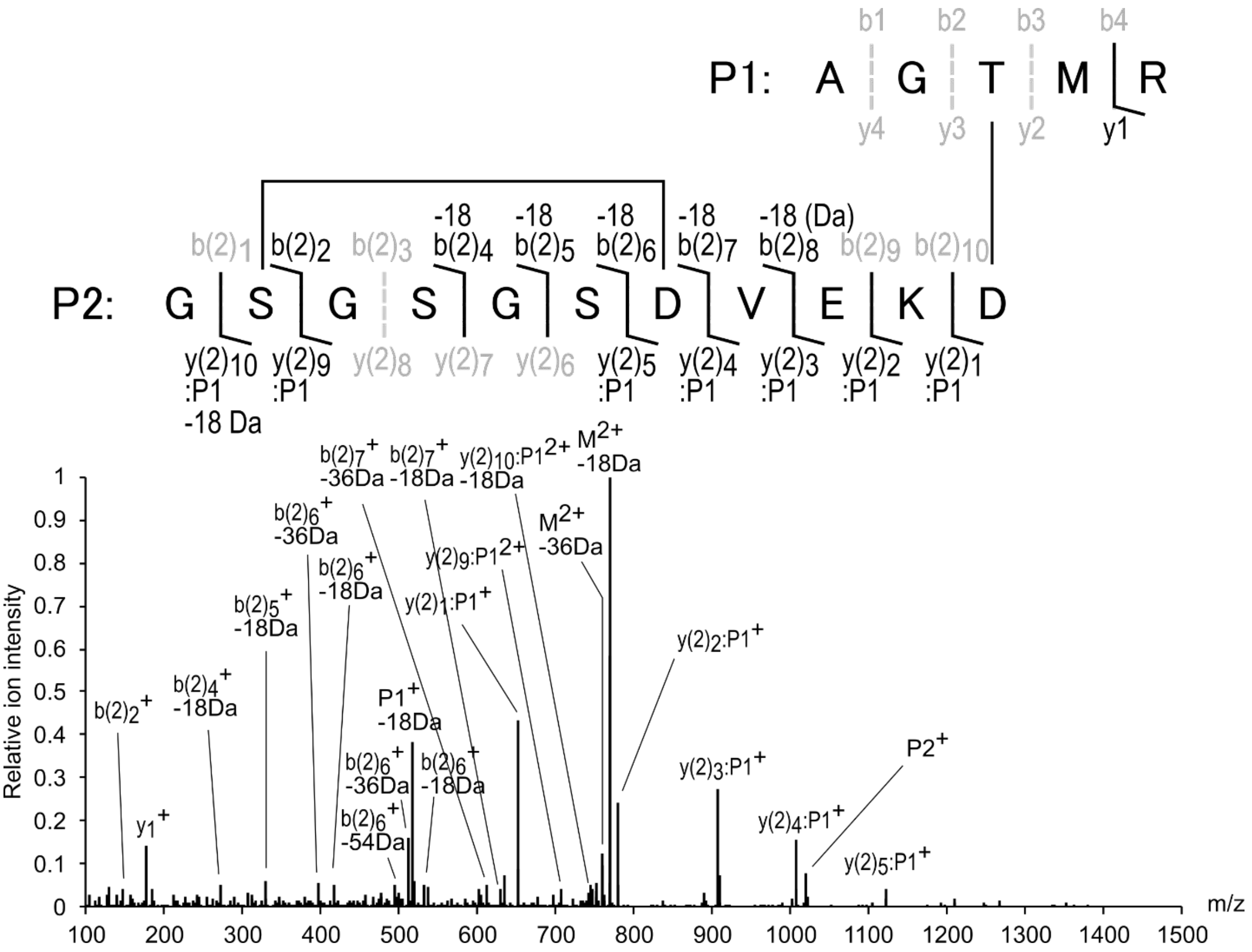

Figure S17. Tandem mass spectrometry analysis of the doubly dehydrated tryptic fragment i (1534.67 Da) obtained from trypsin digestion of mThfA49[B]. All identified ions are listed in Table S9.

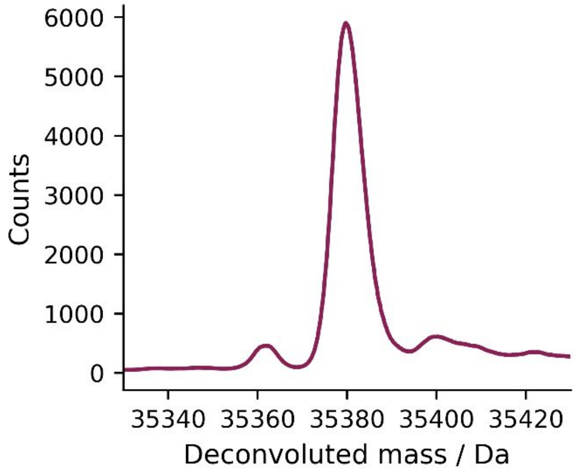

Figure S18. Deconvoluted mass spectra of ThfB isolated from the native purification of mThfA50[B]. An average mass of 35380.6 Da was observed corresponding to ThfB without the N-terminal methionine (calculated average mass = 35380.3 Da).

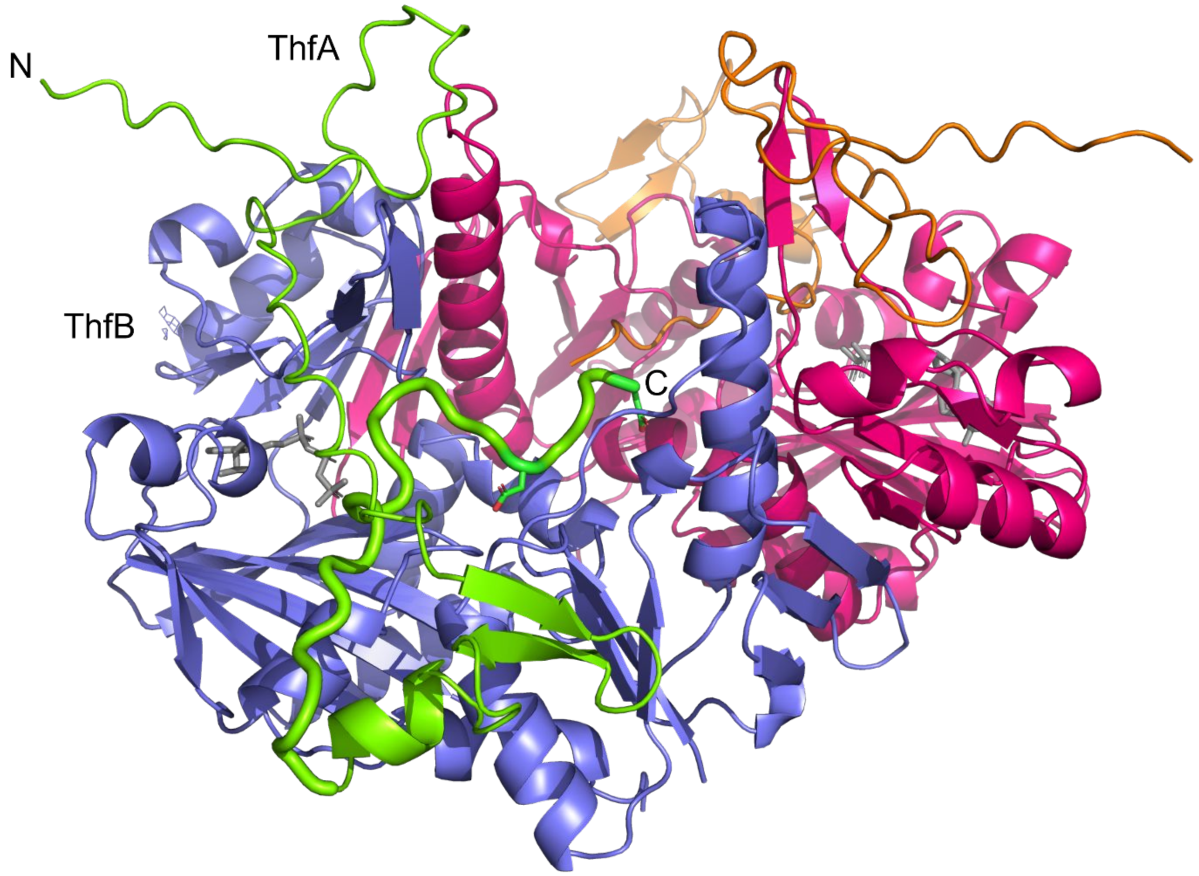

Figure S19. AlphaFold model of two ThfA substrates (green and orange) binding with a dimeric complex of two ThfB enzymes (blue and hot pink) and two ATP ligands (grey). The core peptide sequence of one ThfA molecule (green) is highlighted with a thicker line and the two Asp sidechains that comprise the fuscimiditide stem are shown as sticks. The N- and C-termini of this ThfA are also labeled. The Asp residues corresponding to D18 and D22 of fuscimiditide are relatively far away from the ATP binding site. The leader sequence of ThfA was found to have little secondary structure and minimal binding interactions with ThfB.

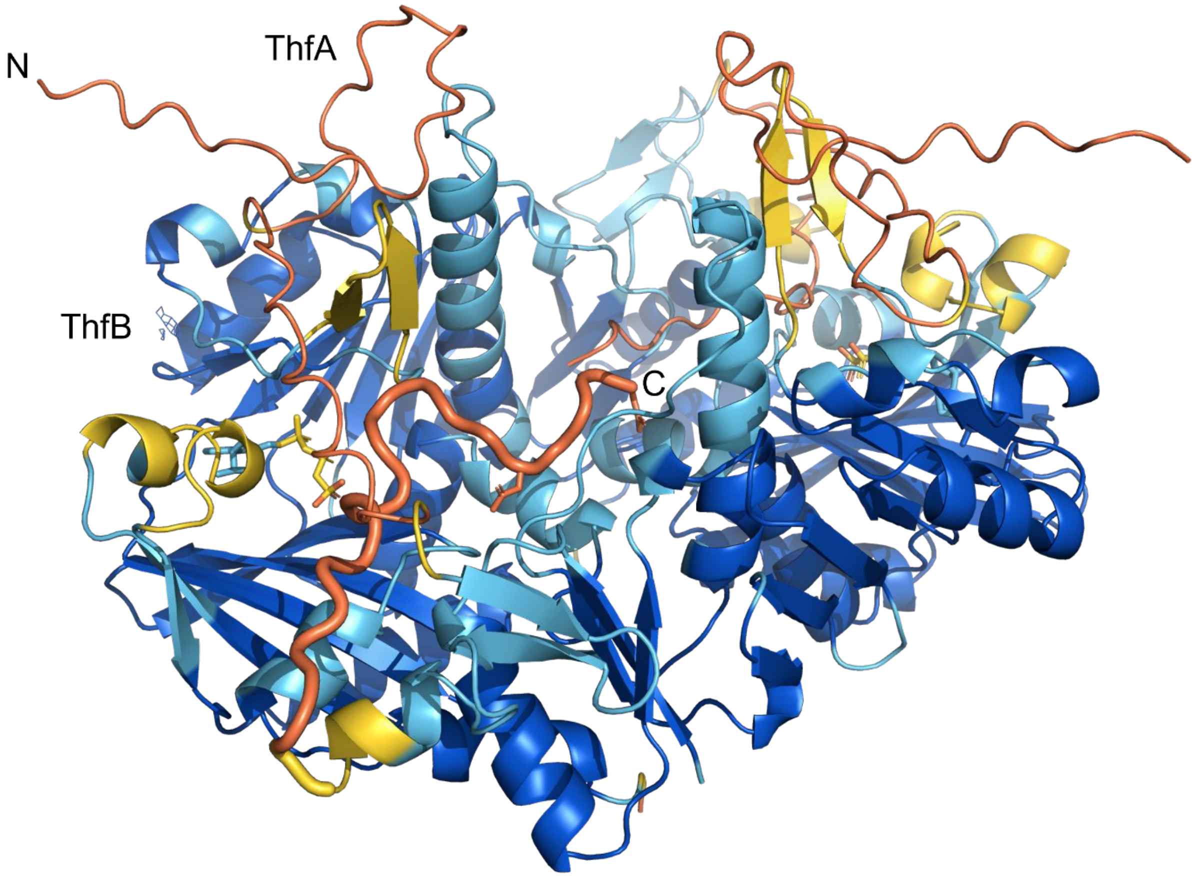

Figure S20. The same AlphaFold model as Figure S17 is shown with two ThfA substrates binding with a dimeric complex of two ThfB enzymes and two ATP ligands, but with the predicted local distance difference test (pLDDT) coloring from AlphaFold. pLDDT is a per-residue measure of local confidence. Regions of very high confidence with pLDDT > 90 are colored dark blue. Regions of confidence with 90 > pLDDT > 70 are colored light blue. Regions of low confidence with 70 > pLDDT > 50 are colored yellow. Regions of very low confidence with pLDDT < 50 are colored orange. While the ThfB enzyme is overall predicted with confidence, the ThfA substrate is predicted with only low confidence.

Supplementary Tables

Table S1: Ions detected from MS/MS analysis of the major peak in the EIC of the net singly dehydrated core peptide fragment from trypsin digestion of mThfA9[B].

Ions	Error (ppm)	Charge
b_2	-3.1	+1
y_{30} (-18 Da) + AGTMR	0.7	+3
y_{29} (-54 Da) + AGTMR	4.4	+3
y_{29} (-36 Da) + AGTMR	2.5	+3
y_{29} (-18 Da) + AGTMR	7.9	+3
y_{19} (-18 Da) + AGTMR	2.7	+2
y_{18} (-18 Da) + AGTMR	1.2	+2
y_{17} (-18 Da) + AGTMR	1.2	+2
y_{15} (-18 Da) + AGTMR	-4.2	+2
y_1 + AGTMR	-0.4	+1
**M** (-72 Da)	3.5	+4
**M** (-54 Da)	1.6	+4
**M** (-36 Da)	-0.5	+4
**M** (-18 Da)	-2.4	+4
AGTMR (-18 Da)	7.1	+1

Table S2: Ions detected from MS/MS analysis of the minor peak in the EIC of the net singly dehydrated core peptide fragment from trypsin digestion of mThfA9[B].

Ions	Error (ppm)	Charge
b_2	-3.0	+1
b_{12}	1.2	+1
b_{14} (-18 Da)	-6.1	+2
b_{14}	-2.7	+1
b_{15} (-18 Da)	-3.0	+2
b_{15}	-3.7	+2
b_{16} (-18 Da)	2.5	+2
b_{16}	-7.1	+2
b_{17} (-18 Da)	-3.1	+2
y_{29} (-36 Da) + AGTMR	-0.8	+3
y_{29} (-18 Da) + AGTMR	0.1	+3
y_{19} (-18 Da) + AGTMR	-8.7	+3

	1.5	+2
y_{18} (-18 Da) + AGTMR	-9.9	+3
	-2.7	+2
y_{17} (-18 Da) + AGTMR	-1.6	+2
y_{16} (-18 Da) + AGTMR	-1.3	+2
y_{15} (-18 Da) + AGTMR	1.3	+3
	-0.6	+2
y_{14} (-18 Da) + AGTMR	-2.1	+2
y_{13} (-18 Da) + AGTMR	-0.8	+2
y_{12} (-18 Da) + AGTMR	0.6	+2
y_{11} (-18 Da) + AGTMR	2.2	+2
y_{10} (-18 Da) + AGTMR	1.3	+2
y_9 (-18 Da) + AGTMR	0.4	+2
y_1 + AGTMR	-6.2	+1
**M** (-72 Da)	2.9	+4
**M** (-54 Da)	-1.5	+4
**M** (-36 Da)	2.5	+4
**M** (-18 Da)	-9.0	+4
AGTMR (-18 Da)	2.4	+1

Table S3: Ions detected from MS/MS analysis of the net doubly dehydrated core peptide fragment from trypsin digestion of mThfA9^{B}.

Ions	Error (ppm)	Charge
b_2	-8.5	+1
y_{35} (-54 Da)	-1.8	+4
**M** (-90 Da)	-1.5	+4
**M** (-72 Da)	3.2	+4
**M** (-54 Da)	1.8	+4
GG	-0.4	+1
GS / SG	-8.5	+1
SS (-18 Da)	-12.3	+1
GGS / SGG	1.0	+1
GGS / SGG (-18 Da)	-14.3	+1
GSS / SSG (-18 Da)	-3.7	+1
GSSG / GGSS / SSGG	-6.5	+1
GSSG / GGSS / SSGG (-18 Da)	3.6	+1

SGGSS / SSGGS (-18 Da)	-8.4	+1
GSSGGSSG / GGSSGGSS / SSGGSSGG / SGGSSGGS	-0.8	+1

Table S4: Ions detected from MS/MS analysis of the singly dehydrated core peptide fragment from trypsin digestion of mThfA3[B].

**Ions**	**Error (ppm)**	**Charge**
b_2	6.9	+1
y_{14} (-18 Da)	3.6	+2
y_{13} (-18 Da)	6.2	+2
**M** (-36 Da)	2.7	+3
**M** (-18 Da)	4.7	+3
**M** (-18 Da) - V	4.4	+2
**M** (-18 Da) - VE	4.1	+2
VTG (-18 Da) or EK (-18 Da)	2.1	+1

Table S5: Ions detected from MS/MS analysis of the acyl hydrazide tagged core peptide fragment from trypsin digestion of mThfA16[B].

**Ions**	**Error (ppm)**	**Charge**
b_2	-3.4	+1
y_1 (+14 Da)	-1.2	+1
y_9 (+14 Da)	-2.3	+1
y_{12} (+14 Da)	-2.2	+2
y_{13} (+14 Da)	-0.2	+2
y_{14} (+14 Da)	1.3	+3
	-0.0	+2
y_{15} (+14 Da)	-0.8	+3
	0.1	+2
y_{16} (+14 Da)	-0.1	+2
**M** (+14 Da)	0.3	+3
PDGQKPGQSEVEK (+14 Da)	-0.9	+2

Table S6: Ions detected from MS/MS analysis of the acyl hydrazide tagged core peptide fragment from trypsin digestion of mThfA17[B].

**Ions**	**Error (ppm)**	**Charge**
b_2	-0.7	+1
y_2	1.1	+1
y_9 (+14 Da)	0.3	+1
y_{12} (+14 Da)	0.2	+2
y_{13} (+14 Da)	1.4	+2
y_{14} (+14 Da)	2.5	+3

	1.1	+2
y_{15} (+14 Da)	2.0	+3
	0.5	+2
y_{16} (+14 Da)	-0.1	+2
**M** (+14 Da)	0.5	+3

Table S7: Ions detected from MS/MS analysis of the triply dehydrated core peptide fragment from trypsin digestion of mThfA46^{B}.

Ions	Error (ppm)	Charge
b_3 (-18 Da)	-1.6	+1
b_{13} (-18 Da)	-0.1	+1
b_{15} (-18 Da)	-2.5	+2
b_{16} (-18 Da)	-3.2	+1
	-3.6	+2
b_{17} (-18 Da)	-2.5	+1
	-2.2	+2
b_{18} (-18 Da)	-3.7	+1
	-1.2	+2
b_{20} (-18 Da)	-2.5	+2
b_{21} (-18 Da)	-3.0	+2
b_{22} (-18 Da)	-0.9	+2
b_{23} (-18 Da)	-0.6	+2
b_{24} (-18 Da)	-0.5	+2
b_{25} (-36 Da)	-2.1	+2
b_{37} (-36 Da)	-5.9	+2
b_{38} (-36 Da)	-2.5	+2
b_{39} (-36 Da)	-4.7	+2
b_{40} (-36 Da)	1.0	+2
	-3.6	+3
b_{41} (-36 Da)	-4.2	+2
	-2.8	+3
b_{43} (-36 Da)	-3.6	+3
b_{44} (-36 Da)	-3.9	+3
b_{45} (-36 Da)	-5.4	+2
	7.5	+3
b_{46} (-36 Da)	-0.6	+3
b_{47} (-36 Da)	-4.0	+2
	-0.2	+3
b_{49} (-54 Da)	-7.6	+3
b_{60} (-54 Da)	-7.7	+3
b_{61} (-54 Da)	-7.8	+4
y_1	-0.6	+1
y_2	-6.7	+1
y_3	-0.9	+1
y_4	-4.3	+1
y_{14}	-1.2	+1
	1.9	+2
y_{15}	0.8	+2
y_{16} (-18 Da)	-2.7	+1
	-0.9	+2

y_{17} (-18 Da)	-5.4	+1
	-1.6	+2
y_{18} (-18 Da)	-3.3	+1
	-2.8	+2
y_{19} (-18 Da)	-1.4	+1
	-2.2	+2
y_{22} (-18 Da)	-2.1	+2
y_{23} (-18 Da)	-2.3	+2
y_{24} (-18 Da)	-1.9	+2
y_{25} (-18 Da)	-1.2	+2
y_{39} (-36 Da)	-4.1	+2
y_{40} (-36 Da)	-1.5	+2
y_{41} (-36 Da)	-1.2	+2
y_{42} (-36 Da)	-2.0	+2
y_{45} (-36 Da)	-4.4	+2
	-3.2	+3
y_{46} (-36 Da)	-2.2	+3
y_{47} (-36 Da)	-1.2	+3
**M** (-54 Da)	-5.0	+4

Table S8: Ions detected from MS/MS analysis of the singly dehydrated tryptic fragment i (1552.67 Da) obtained from trypsin digestion of mThfA47^{B}.

Ions	Error (ppm)	Charge
$b(2)_3$ (-18 Da)	-5.1	+1
$b(2)_7$ (-36 Da)	-3.8	+1
$b(2)_7$ (-18 Da)	-5.9	+1
$b(2)_7$	-5.7	+1
$y(2)_1$:P1	-3.4	+1
$y(2)_3$:P1	-9.5	+1
$y(2)_4$:P1	-9.3	+1
$y(2)_5$:P1	-2.8	+1
$y(2)_6$:P1	-3.8	+1
$y(2)_7$:P1	-2.0	+1
$y(2)_8$:P1	-6.3	+2
$y(2)_9$:P1	-4.1	+2
y_1	-8.0	+1
P1 (-18 Da)	-6.2	+1
**M** (-18 Da)	1.4	+2
**M**	-0.8	+2

Table S9: Ions detected from MS/MS analysis of the doubly dehydrated tryptic fragment i (1534.67 Da) obtained from trypsin digestion of mThfA49^{B}.

Ions	Error (ppm)	Charge
$b(2)_2$	2.4	+1
$b(2)_4$ (-18 Da)	-7.8	+1
$b(2)_5$ (-18 Da)	0.8	+1
$b(2)_6$ (-36 Da)	-1.1	+1
$b(2)_6$ (-18 Da)	0.5	+1

$b(2)_7$ (-54 Da)	-0.6	+1
$b(2)_7$ (-36 Da)	0.1	+1
$b(2)_7$ (-18 Da)	1.2	+1
$b(2)_8$ (-36 Da)	-7.8	+1
$b(2)_8$ (-18 Da)	-0.3	+1
$y(2)_1$:P1	3.0	+1
$y(2)_2$:P1	-1.4	+1
$y(2)_3$:P1	0.9	+1
$y(2)_4$:P1	-5.7	+1
$y(2)_5$:P1	-1.0	+1
$y(2)_9$:P1	-0.8	+2
$y(2)_{10}$:P1 (-18 Da)	-2.2	+2
y_1	2.0	+1
P1 (-18 Da)	-1.5	+1
P2	-0.2	+1
**M** (-36 Da)	5.2	+2
**M** (-18 Da)	1.2	+2

Table S10: Oligonucleotides used in this study.

Name	Sequence (5' to 3')	Reference
oBC035	CGATGGTCTCAAGCTTTTAGTTTTCCTTCCTTGTGAGAGC	Elashal et al. 2022[2]
oBC279	GCATGGTCTCTGATCCTCGACAGCGGTCACC	This study
oBC352	GCATGGTCTCTCATGACCGTTCTCATCCTC	This study
oBC355	CGATGGTCTCAatccTTTTCCACGTCGCTCTG	This study
oBC356	GCATGGTCTCTggatGGTGATCCATCTGCCGGCACCATGAG	This study
oBC357	CGATGGTCTCAAGCTTCTAGTCTTTTTCCACGTCG	This study
oBC359	CGATGGTCTCTGCCGGCTCTTTCGATTTTC	This study
oBC360	GCATGGTCTCTCGGCTGTATGAGAGTCACCTATCCCG	This study
oBC361	GCATGGTCTCTCGGCAAAATGAGAGTCACCTATCCCG	This study
oBC362	CGATGGTCTCTGACTCTCATGGTGCCG	This study
oBC363	GCATGGTCTCTAGTCTGTTATCCCGACGGCCAG	This study
oBC364	GCATGGTCTCTAGTCAAATATCCCGACGGCCAG	This study
oBC367	AGTCACCagcggtagcggtagcggtagcggtAGCGACGTGGAAAAAGACTAGA	This study
oBC368	AGCTTCTAGTCTTTTTCCACGTCGCTaccgctaccgctaccgctaccgctGGT	This study
oBC371	GCATGGTCTCTCACATCTTTCGATTTTCGCGC	This study
oBC372	GCATGGTCTCTTGTGGCTGTATGAGAGTCACC	This study
oBC373	GCATGGTCTCTtaccGGTGACTCTCATGGTG	This study
oBC374	ggtagcggtagcggtagcggtAGCGACGTGGAAAAAGACTAGA	This study
oBC375	AGCTTCTAGTCTTTTTCCACGTCGCTaccgctaccgctaccgc	This study
oBC376	ggtagcggtagcggtAGCGACGTGGAAAAAGACTAGA	This study
oBC377	AGCTTCTAGTCTTTTTCCACGTCGCTaccgctaccgc	This study
oBC378	ggtagcggtagcggtagcggtagcggtagcggtAGCGACGTGGAAAAAGACTAGA	This study
oBC379	AGCTTCTAGTCTTTTTCCACGTCGCTaccgctaccgctaccgctaccgctaccgc	This study
oBC380	GCATGGTCTCTggatagtggtagcggctccggatcgggtagcggcACCATGAGAGTCAC C	This study
oBC381	GCATGGTCTCTggatagtggtagcggctccggaACCATGAGAGTCACC	This study
oBC401	GCATGGTCTCTAGCTTCTAGTCTTTTTCCAC	This study
oBC402	GCATGGTCTCTCGGCGTTATGAGAGTCACCTATCCCGACGGCCAGAAGC CG	This study
oBC403	GCATGGTCTCTAGCTTCTAGTTTTTTTCCACGTCGCTCTGCCCCGGCTTC TGGCCGTC	This study
oBC404	GCATGGTCTCTCGGCACCATGAGAGTCGTTTATCCCGACGGCCAGAAGC CG	This study
oBC405	GCATGGTCTCTAGCTTCTAGTCTTTTTCCACGTTGCTCTGCCCCGGCTTC TGGCCGTC	This study
oBC408	GCATGGTCTCTctgtTCTTTCGATTTTCGCGC	This study
oBC409	GCATGGTCTCTacaGGCACCATGAGAGTC	This study
oBC410	GCATGGTCTCTagcttCTAATCAGAGTCTTTTTCCACGTCG	This study
oBC411	ggtagcggtAGCGACGTGGAAAAAGACTAGA	This study
oBC412	AGCTTCTAGTCTTTTTCCACGTCGCTaccgc	This study
oBC413	ggtAGCGACGTGGAAAAAGACTAGA	This study
oBC414	AGCTTCTAGTCTTTTTCCACGTCGC	This study
oBC415	GCATGGTCTCTggtagcggtagcggtagccgtaaaggcgaagagc	This study
oBC417	GCATGGTCTCTcggcACCggtagcggtACCTATCCCGACGG	This study
oBC418	GCATGGTCTCT agcttCTAGTCaccgctaccGTCGCTCTGCCCC	This study
oBC425	ggtAGCGGTGACGTGGAAAAAGACTAGA	This study
oBC426	AGCTTCTAGTCTTTTTCCACGTCACCGC	This study
oBC427	GCATGGTCTCTggatAGTGGTAGTGGCACCATGAGAGTC	This study

oBC428	GCATGGTCTCTggatAGTGGCACCATGAGAGTC	This study
oBC429	GCATGGTCTCTggatGGCACCATGAGAGTC	This study
oBC430	GCATGGTCTCTggatACCATGAGAGTCACCTATC	This study
oBC433	GCATGGTCTCTttcctttgtacagttcatccatacc	This study
oBC434	ggaagcggtagcggtAGCGACGTGGAAAAAGACTAGA	This study
oBC444	GCATGGTCTCTgtctTTTTCCACGTCG	This study
oBC445	GCATGGTCTCTagacAGTGGCACCATGAGAGTC	This study
oBC446	GCATGGTCTCAagcttTCAGTCCTTCTCTACGTCGCTCTGCCC	This study
oBC450	GCATGGTCTCTgattTTCGCGCACGG	This study
oBC451	GCATGGTCTCTaatcACCAGAGCCGGCACCATG	This study
oBC452	GCATGGTCTCTaatcGAAAGAGCCaccACCATGAGAGTCACC	This study
oBC453	GCATGGTCTCTcggcACCATGACCGTCACCTATCCCGACG	This study
oBC454	GCATGGTCTCAagcttCTAATCTTTTTCAACGTCTTTTTCCACGTCGC	This study
oBC455	GCATGGTCTCAagcttCTAGTCGTCTTTTTCCACGTCGC	This study
oBC456	GCATGGTCTCAagcttCTAGTCTTTGTCCACGTCGCTCTG	This study
oBC457	cggcACCATGAGAGTCACCGACGTGGAAAAAGACTAGa	This study
oBC458	agcttCTAGTCTTTTTCCACGTCGGTGACTCTCATGGT	This study
oBC459	cggcACCATGAGAGTCGTGGAAAAAGACTAGa	This study
oBC460	agcttCTAGTCTTTTTCCACGACTCTCATGGT	This study
oBC464	cggcACCATGAGAGTCACCGTGGAAAAAGACTAGa	This study
oBC465	agcttCTAGTCTTTTTCCACGGTGACTCTCATGGT	This study
oBC466	cggcACCATGAGAGTCGACGTGGAAAAAGACTAGa	This study
oBC467	agcttCTAGTCTTTTTCCACGTCGACTCTCATGGT	This study
oBC468	GCATGGTCTCTaatcACCatgcgtgttACCATGAGAGTCACCTATC	This study
oBC512	GCATGGTCTCTggtagcggtagcggtagcGTGTCCAAAGGAGAGG	This study
oBC513	GCATGGTCTCTttcccttatacaattcatccataccacc	This study
oBC514	GCATGGTCTCTggtagcggtagcggtagcgtttctaaaggtgaagaattattcac	This study
oBC515	GCATGGTCTCTttcctttgtacaattcatccataccc	This study
oBC524	TcaccAGAGACCgaaagtgaaacgtg	This study
oBC525	AcgtcAGAGACCtataaacgcagaaagg	This study
oBC526	GCATGGTCTCTggtgACTCTCATGGTGC	This study
oBC527	gacgTGGAAAAAGACTAGA	This study
oBC528	agctTCTAGTCTTTTTCCA	This study
oBC529	GCATGGTCTCTcaccGGTAGTTCTGGTGGGTC	This study
oBC530	GCATGGTCTCTcgtcTCCGCTACTCCCACC	This study
oBC531	GCATGGTCTCTcgtcCCCGCTAGAGCCC	This study
oBC549	GCATGGTCTCTaatcACCATGAGAGTCACCGGTTCCGGTACCTATCCCGAC GGCCAG	This study
oBC550	GCATGGTCTCTagcttCTAATCTTTTTCAACGTCACCAGAACCGTCGCTCTG CCCCG	This study
oBC554	GCATGGTCTCTGCCGGGACCGTTTGCTTCCACGTCGCTCTG	This study
oBC560	GCATGGTCTCTAGCTTCTAATCTTTTTCAACGTCGCCTTCACCGTCGCTCT G	This study
oBC561	GCATGGTCTCTAGCTTCTAATCTTTTTCAACGTCGCCTTGACCGTCGCTCT G	This study
oBC566	GCATGGTCTCTgatccGTGTCCAAAGGAGAGG	This study
oBC567	GCATGGTCTCTAGCTTCTActtatacaattcatccataccacc	This study
oBC576	CACCTCCTCCTCCACCGGTGACTCTCATGGTGCC	This study
oBC577	GCATGGATCCAGTCGACAGCGGTCACC	This study
oBC578	GGTGGAGGAGGAGGT	This study
oBC579	GCATAAGCTTTTATTTCTTTTTCAGTTTATCCAGCTGC	This study
oBC618	GCATCATATGGGTCCGGGTAGCGATAGCGAA	This study

oBC619	CAGAGACTCGAGTTAATCCTTCTCAACATCACCTCCTCCACCACCTCCTTT CTTTTTCAGTTTATCCAGCTGC	This study
oAA044	CGATggtctcAtcttTCGATTTTCGCGC	This study
oAA045	CGATggtctcAaagaGCAGGGACGATGAGGGTCTATACCCCCGACGGCCAG	This study
oAA046	CGATggtctcAaagaGCAGGAACGATGAGGACCGTCTATCCCGACGGCC	This study
oAA047	CGATggtctcAaagaGCCGGAATGACCAGAGTCACCTATCCCGAC	This study
oAA048	CGATggtctcAaagaGCCACCGGCATGAGAGTCACCTATCCC	This study
oAA049	CGATGGTCTCAagctCTAATCTTTCTCCACGCTGTCCTGCCCCGGCTTC	This study
oAA050	CGATGGTCTCAagctCTAGTCCTTTTCGTCCACGCTCTGCCCCG	This study
oAA051	CGATGGTCTCAagctCTATTTGTCTTCCACGTCGCTCTG	This study
oAA052	CGATGGTCTCAagctCTAGTCTTTTTCCACGTCG	This study
oAA053	CGATggtctcAcggcACGATGgccGTCACCTATCCCGACG	This study
oAA054	CGATGGTCTCAagctTCTAGTCTTTGGCCACGTCGCTCTGC	This study
oHE1	TATGGATCCGATGTCGACAGCGGTCACC	Elashal et al. 2022[2]
oHE2	ATAAAGCTTCTAGTCTTTTTCCACGTCGC	Elashal et al. 2022[2]
oHE3	GAAAATCGAAAGAGCCGGCTCTATGAGAGT	This study
oHE4	ATTATGCGGCCGTGTACAATACG	Elashal et al. 2022[2]
oHE5	GCACCATGAGAGTCTCTTATCCCGAC	This study
oHE6	CCCCGCTCCAAGCTTCTAGTCTTTTTCCACTGCGCTCTG	This study
oHE7	CCCCGCTCCAAGCTTCTATTCTTTTTCCACGTCGCTCTG	This study
oTGJ023	ATTCGGTCTCTCTAGCTTGGATTCTCACCAATAAAAAACG	This study
oTGJ026	GCATTAGGTCTCTGATCCTCGACAGCGGTCAC	This study
oTGJ061	ATTCGTGGTCTCTgataATCTTTTTCCACATCGCCGGCTCTTTCGATTTTC	This study
oTGJ062	TGCATTGGTCTCTtatcCCGACGGCCAGAAG	This study
oTGJ063	TGTCTTGGTCTCAAGCTTTTACGTGACTCTCATGGTGCTCTGCCCCGG	This study

Table S11: Plasmids used for protein expression in this study.

Plasmid Name	Vector	Oligonucleotides Used	Name of the Expressed Protein	Sequence of the Expressed Protein (Underlined: core peptide; Bolded: mutation)	Reference
pThfA	pRSF-duet	See Reference	His6-ThfA	MGSSHHHHHHSQDPMSTAVTDAFPLGRDENRNDQVTEWRPFGMRYGVQPTPIPVPLSDTKYDPDQQVLVVADGQPCAKIERAGTMRVTYPDGQKPGQSDVEKD	Elashal et al. 2022[2]
pJDK130	pQE-80	See reference	ThfB	MTVLILTNPFDITADDVILRLTERGVPVVRLDPADFPQQVVLHSEIGGNGWTGTLTTPHRILDLSTVTGIWYRRPRKFRLPAQMSQAEYEFAATEARRGFGGIINSLTGWINHPSAIGRAEYKPYQLHHAVQAGLNVPRTLITNDPKQAKGWCARVGDVVYKPLSAPSWLENGDTYVVFTTPITPDQWGDPAIGRTAHMFQQRLDKEFEVRLTMVDGKAFPAAIHAHSDAARIDWRSDYDALTYSIPTVPQRVLTGARDLLRRLHLRYAALDFIVSPDGRWHFLEVNPNGQYGWIEEHTGQPISDAIADALTRKEN	Elashal et al. 2022[2]
pBC262	pRSF-duet	oBC035, oBC352	ThfB	MTVLILTNPFDITADDVILRLTERGVPVVRLDPADFPQQVVLHSEIGGNGWTGTLTTPHRILDLSTVTGIWYRRPRKFRLPAQMSQAEYEFAATEARRGFGGIINSLTGWINHPSAIGRAEYKPYQLHHAVQAGLNVPRTLITNDPKQAKGWCARVGDVVYKPLSAPSWLENGDTYVVFTTPITPDQWGDPAIGRTAHMFQQRLDKEFEVRLTMVDGKAFPAAIHAHSDAARIDWRSDYDALTYSIPTVPQRVLTGARDLLRRLHLRYAALDFIVSPDGRWHFLEVNPNGQYGWIEEHTGQPISDAIADALTRKEN	This study
pBC108	pQE-80	See Reference	sfGFP	See Reference	Choi et al. 2022[1]
pBC253	pBC108	oBC279, oBC355, oBC356, oBC357	**ThfA38** His6-ThfA divalent bicyclic core peptide with GDPSAG linker	MSGSHHHHHHGSSTAVTDAFPLGRDENRNDQVTEWRPFGMRYGVQPTPIPVPLSDTKYDPDQQVLVVADGQPCAKIERAGTMRVTYPDGQKPGQSDVEKD**GDPSAGTMRVTYPDGQKPGQSDVEKD**	This study
pBC259	pBC108	oBC279, oBC362,	**ThfA7** His6-ThfA	MSGSHHHHHHGSSTAVTDAFPLGRDENRNDQVTEWRPFG	This study

		oBC367,<br>oBC368	S(GS)$_4$ loop	MRYGVQPTPIPVPLSDTKYDP<br>DQQVLVVADGQPCAKIERAGT<br>MRVT**SGSGSGSGS**DVEKD	
pBC268	pBC108	oBC279,<br>oBC373,<br>oBC374,<br>oBC375	**ThfA6**<br>His6-ThfA<br>(GS)$_4$ loop	MSGSHHHHHHGSSTAVTDAF<br>PLGRDENRNDQVTEWRPFG<br>MRYGVQPTPIPVPLSDTKYDP<br>DQQVLVVADGQPCAKIERAGT<br>MRVT**GSGSGSGS**DVEKD	This study
pBC269	pBC108	oBC279,<br>oBC373,<br>oBC376,<br>oBC377	**ThfA5**<br>His6-ThfA<br>(GS)$_3$ loop	MSGSHHHHHHGSSTAVTDAF<br>PLGRDENRNDQVTEWRPFG<br>MRYGVQPTPIPVPLSDTKYDP<br>DQQVLVVADGQPCAKIERAGT<br>MRVT**GSGSGS**DVEKD	This study
pBC270	pBC108	oBC279,<br>oBC373,<br>oBC378,<br>oBC379	**ThfA8**<br>His6-ThfA<br>(GS)$_6$ loop	MSGSHHHHHHGSSTAVTDAF<br>PLGRDENRNDQVTEWRPFG<br>MRYGVQPTPIPVPLSDTKYDP<br>DQQVLVVADGQPCAKIERAGT<br>MRVT**GSGSGSGSGSGS**DVEK<br>D	This study
pBC271	pBC108	oBC279,<br>oBC355,<br>oBC380,<br>oBC401	**ThfA39**<br>His6-ThfA divalent bicyclic core peptide with (SG)$_5$ linker	MSGSHHHHHHGSSTAVTDAF<br>PLGRDENRNDQVTEWRPFG<br>MRYGVQPTPIPVPLSDTKYDP<br>DQQVLVVADGQPCAKIERAGT<br>MRVTYPDGQKPGQSDVEKD**S**<br>**GSGSGSGSGTMRVTYPDGQ**<br>**KPGQSDVEKD**	This study
pBC272	pBC108	oBC279,<br>oBC355,<br>oBC381,<br>oBC401	**ThfA40**<br>His6-ThfA divalent bicyclic core peptide with (SG)$_3$ linker	MSGSHHHHHHGSSTAVTDAF<br>PLGRDENRNDQVTEWRPFG<br>MRYGVQPTPIPVPLSDTKYDP<br>DQQVLVVADGQPCAKIERAGT<br>MRVTYPDGQKPGQSDVEKD**S**<br>**GSGSGTMRVTYPDGQKPGQ**<br>**SDVEKD**	This study
pBC273	pBC108	oBC279,<br>oBC359,<br>oBC402,<br>oBC403	**ThfA11**<br>His6-ThfA<br>T3V/D22N	MSGSHHHHHHGSSTAVTDAF<br>PLGRDENRNDQVTEWRPFG<br>MRYGVQPTPIPVPLSDTKYDP<br>DQQVLVVADGQPCAKIERAG<br>**V**MRVTYPDGQKPGQSDVEK**N**	This study
pBC274	pBC108	oBC279,<br>oBC359,<br>oBC404,<br>oBC405	**ThfA12**<br>His6-ThfA<br>T7V/D18N	MSGSHHHHHHGSSTAVTDAF<br>PLGRDENRNDQVTEWRPFG<br>MRYGVQPTPIPVPLSDTKYDP<br>DQQVLVVADGQPCAKIERAGT<br>MRV**V**YPDGQKPGQS**N**VEKD	This study
pBC276	pBC108	oBC279,<br>oBC408,<br>oBC409,<br>oBC410	**ThfA29**<br>His6-ThfA<br>tricyclic variant	MSGSHHHHHHGSSTAVTDAF<br>PLGRDENRNDQVTEWRPFG<br>MRYGVQPTPIPVPLSDTKYDP<br>DQQVLVVADGQPCAKIER**T**GT<br>MRVTYPDGQKPGQSDVEKD**S**<br>**D**	This study

pBC277	pBC108	oBC279, oBC373, oBC411, oBC412	**ThfA4**<br>His6-ThfA<br>$(GS)_2$ loop	MSGSHHHHHHGSSTAVTDAFPLGRDENRNDQVTEWRPFGMRYGVQPTPIPVPLSDTKYDPDQQVLVVADGQPCAKIERAGTMRVT**GSGS**DVEKD	This study
pBC278	pBC108	oBC279, oBC373, oBC413, oBC414	**ThfA2**<br>His6-ThfA<br>GS loop	MSGSHHHHHHGSSTAVTDAFPLGRDENRNDQVTEWRPFGMRYGVQPTPIPVPLSDTKYDPDQQVLVVADGQPCAKIERAGTMRVT**GS**DVEKD	This study
pBC279	pBC108	oBC279, oBC373, oBC415, oBC433, oBC434, oBC377	**ThfA48**<br>His6-ThfA<br>sfGFP-inserted	MSGSHHHHHHGSSTAVTDAFPLGRDENRNDQVTEWRPFGMRYGVQPTPIPVPLSDTKYDPDQQVLVVADGQPCAKIERAGTMRVT**GSGSGSRKGEELFTGVVPILVELDGDVNGHKFSVRGEGEGDATNGKLTLKFICTTGKLPVPWPTLVTTLTYGVQCFARYPDHMKQHDFFKSAMPEGYVQERTISFKDDGTYKTRAEVKFEGDTLVNRIELKGIDFKEDGNILGHKLEYNFNSHNVYITADKQKNGIKANFKIRHNVEDGSVQLADHYQQNTPIGDGPVLLPDNHYLSTQSVLSKDPNEKRDHMVLLEFVTAAGITHGMDELYKGSGSGS**DVEKD	This study
pBC280	pBC108	oBC279, oBC359, oBC417, oBC357	**ThfA18**<br>His6-ThfA<br>GSG-substitution at N-terminal stem	MSGSHHHHHHGSSTAVTDAFPLGRDENRNDQVTEWRPFGMRYGVQPTPIPVPLSDTKYDPDQQVLVVADGQPCAKIERAGT**GSG**TYPDGQKPGQSDVEKD	This study
pBC281	pBC108	oBC279, oBC418	**ThfA19**<br>His6-ThfA<br>GSG-substitution at C-terminal stem	MSGSHHHHHHGSSTAVTDAFPLGRDENRNDQVTEWRPFGMRYGVQPTPIPVPLSDTKYDPDQQVLVVADGQPCAKIERAGTMRVTYPDGQKPGQSD**GSG**D	This study
pBC305	pBC108	oBC279, oBC373, oBC425, oBC426	**ThfA3**<br>His6-ThfA<br>GSG loop	MSGSHHHHHHGSSTAVTDAFPLGRDENRNDQVTEWRPFGMRYGVQPTPIPVPLSDTKYDPDQQVLVVADGQPCAKIERAGTMRVT**GSG**DVEKD	This study
pBC306	pBC108	oBC279, oBC355, oBC401, oBC427	**ThfA41**<br>His6-ThfA divalent bicyclic core peptide with $(SG)_2$ linker	MSGSHHHHHHGSSTAVTDAFPLGRDENRNDQVTEWRPFGMRYGVQPTPIPVPLSDTKYDPDQQVLVVADGQPCAKIERAGTMRVTYPDGQKPGQSDVEKD**SGSGTMRVTYPDGQKPGQSDVEKD**	This study

pBC307	pBC108	oBC279, oBC355, oBC401, oBC428	**ThfA42** His6-ThfA divalent bicyclic core peptide with SG linker	MSGSHHHHHHGSSTAVTDAFPLGRDENRNDQVTEWRPFGMRYGVQPTPIPVPLSDTKYDPDQQVLVVADGQPCAKIERAGTMRVTYPDGQKPGQSDVEKD**SGTMRVTYPDGQKPGQSDVEKD**	This study
pBC308	pBC108	oBC279, oBC355, oBC401, oBC429	**ThfA43** His6-ThfA divalent bicyclic core peptide with G linker	MSGSHHHHHHGSSTAVTDAFPLGRDENRNDQVTEWRPFGMRYGVQPTPIPVPLSDTKYDPDQQVLVVADGQPCAKIERAGTMRVTYPDGQKPGQSDVEKD**GTMRVTYPDGQKPGQSDVEKD**	This study
pBC309	pBC108	oBC279, oBC355, oBC430, oBC401	**ThfA44** His6-ThfA divalent bicyclic core peptide with no linker	MSGSHHHHHHGSSTAVTDAFPLGRDENRNDQVTEWRPFGMRYGVQPTPIPVPLSDTKYDPDQQVLVVADGQPCAKIERAGTMRVTYPDGQKPGQSDVEKD**TMRVTYPDGQKPGQSDVEKD**	This study
pBC315	pBC108	oBC279, oBC444, oBC445, oBC446	**ThfA45** His6-ThfA trivalent bicyclic core peptide with SG linker	MSGSHHHHHHGSSTAVTDAFPLGRDENRNDQVTEWRPFGMRYGVQPTPIPVPLSDTKYDPDQQVLVVADGQPCAKIERAGTMRVTYPDGQKPGQSDVEKD**SGTMRVTYPDGQKPGQSDVEKDSGTMRVTYPDGQKPGQSDVEKD**	This study
pBC317	pBC108	oBC279, oBC450, oBC451, oBC454	**ThfA33** His6-ThfA tricyclic variant	MSGSHHHHHHGSSTAVTDAFPLGRDENRNDQVTEWRPFGMRYGVQPTPIPVPLSDTKYDPDQQVLVVADGQPCAK**IT**RAGTMRVTYPDGQKPGQSDVEKD**VEKD**	This study
pBC318	pBC108	oBC279, oBC450, oBC452, oBC455	**ThfA31** His6-ThfA tricyclic variant	MSGSHHHHHHGSSTAVTDAFPLGRDENRNDQVTEWRPFGMRYGVQPTPIPVPLSDTKYDPDQQVLVVADGQPCAKIERA**T**TMRVTYPDGQKPGQSDVEKD**D**	This study
pBC319	pBC108	oBC279, oBC359, oBC453, oBC456	**ThfA30** His6-ThfA tricyclic variant	MSGSHHHHHHGSSTAVTDAFPLGRDENRNDQVTEWRPFGMRYGVQPTPIPVPLSDTKYDPDQQVLVVADGQPCAKIERAGTM**T**VTYPDGQKPGQSDV**D**KD	This study
pBC320	pBC108	oBC279, oBC359, oBC457, oBC458	**ThfA1** His6-ThfA no loop	MSGSHHHHHHGSSTAVTDAFPLGRDENRNDQVTEWRPFGMRYGVQPTPIPVPLSDTKYDPDQQVLVVADGQPCAKIERAGTMRVTDVEKD	This study

pBC321	pBC108	oBC279, oBC359, oBC459, oBC460	**ThfA22**<br>His6-ThfA<br>Δ7-18	MSGSHHHHHHGSSTAVTDAFPLGRDENRNDQVTEWRPFGMRYGVQPTPIPVPLSDTKYDPDQQVLVVADGQPCAKIERAGTMRVVEKD	This study
pBC324	pBC108	oBC279, oBC359, oBC464, oBC465	**ThfA23**<br>His6-ThfA<br>Δ8-18	MSGSHHHHHHGSSTAVTDAFPLGRDENRNDQVTEWRPFGMRYGVQPTPIPVPLSDTKYDPDQQVLVVADGQPCAKIERAGTMRVTVEKD	This study
pBC325	pBC108	oBC279, oBC359, oBC466, oBC467	**ThfA24**<br>His6-ThfA<br>Δ7-17	MSGSHHHHHHGSSTAVTDAFPLGRDENRNDQVTEWRPFGMRYGVQPTPIPVPLSDTKYDPDQQVLVVADGQPCAKIERAGTMRVDVEKD	This study
pBC326	pBC108	oBC279, oBC450, oBC468, oBC454	**ThfA32**<br>His6-ThfA<br>tricyclic variant	MSGSHHHHHHGSSTAVTDAFPLGRDENRNDQVTEWRPFGMRYGVQPTPIPVPLSDTKYDPDQQVLVVADGQPCAK**ITMRV**TMRVTYPDGQKPGQSDVEKD**VEKD**	This study
pBC343	pBC108	oBC279, oBC373, oBC512, oBC513, oBC434, oBC377	**ThfA47**<br>His6-ThfA<br>mRuby2-inserted	MSGSHHHHHHGSSTAVTDAFPLGRDENRNDQVTEWRPFGMRYGVQPTPIPVPLSDTKYDPDQQVLVVADGQPCAKIERAGTMRVT**GSGSGSVSKGEELIKENMRMKVVMEGSVNGHQFKCTGEGEGNPYMGTQTMRIKVIEGGPLPFAFDILATSFMYGSRTFIKYPKGIPDFFKQSFPEGFTWERVTRYEDGGVVTVMQDTSLEDGCLVYHVQVRGVNFPSNGPVMQKKTKGWEPNTEMMYPADGGLRGYTHMALKVDGGGHLSCSFVTTYRSKKTVGNIKMPGIHAVDHRLERLEESDNEMFVVQREHAVAKFAGLGGGMDELYKGSGSGS**DVEKD	This study
pBC344	pBC108	oBC279, oBC373, oBC514, oBC515, oBC434, oBC377	**ThfA49**<br>His6-ThfA<br>mTurquoise2-inserted	MSGSHHHHHHGSSTAVTDAFPLGRDENRNDQVTEWRPFGMRYGVQPTPIPVPLSDTKYDPDQQVLVVADGQPCAKIERAGTMRVT**GSGSGSVSKGEELFTGVVPILVELDGDVNGHKFSVSGEGEGDATYGKLTLKFICTTGKLPVPWPTLVTTLSWGVQCFARYPDHMKQHDFFKSAMPEGYVQERTIFFKDDGNYKTRAEVKFEGDTLVNRIELKGIDFKEDGNILGHKLEYNYFSDNVYITAD**	This study

				**KQKNGIKANFKIRHNIEDGGVQLADHYQQNTPIGDGPVLLPDNHYLSTQSKLSKDPNEKRDHMVLLEFVTAAGITLGMDELYKGSGSGS**DVEKD	
pBC348	pBC108	oBC279, oBC526, oBC524, oBC525, oBC527, oBC528	pBC108-like entry vector for fuscimiditide loop substitution	Same as pBC108	This study
pBC349	pBC348	oBC529, oBC530	**ThfA9**<br>His6-ThfA<br>(GSSG)$_6$ loop	MSGSHHHHHHGSSTAVTDAFPLGRDENRNDQVTEWRPFGMRYGVQPTPIPVPLSDTKYDPDQQVLVVADGQPCAKIERAGTMRVT**GSSGGSSGGSSGGSSGGSSGGSSG**DVEKD	This study
pBC350	pBC348	oBC529, oBC531	**ThfA10**<br>His6-ThfA<br>(GSSG)$_{18}$ loop	MSGSHHHHHHGSSTAVTDAFPLGRDENRNDQVTEWRPFGMRYGVQPTPIPVPLSDTKYDPDQQVLVVADGQPCAKIERAGTMRVT**GSSGGSSGGSSGGSSGGSSGGSSGGSSGGSSGGSSGGSSGGSSGGSSGGSSGGSSGGSSGGSSGGSSGGSSG**DVEKD	This study
pBC363	pBC108	oBC279, oBC450, oBC549, oBC454	**ThfA34**<br>His6-ThfA<br>tricyclic variant	MSGSHHHHHHGSSTAVTDAFPLGRDENRNDQVTEWRPFGMRYGVQPTPIPVPLSDTKYDPDQQVLVVADGQPCAK**ITMRV**T**GSG**TYPDGQKPGQSDVEKD**VEKD**	This study
pBC364	pBC108	oBC279, oBC550	**ThfA35**<br>His6-ThfA<br>tricyclic variant	MSGSHHHHHHGSSTAVTDAFPLGRDENRNDQVTEWRPFGMRYGVQPTPIPVPLSDTKYDPDQQVLVVADGQPCAK**ITMRV**T**GSG**TYPDGQKPGQSD**GSG**D**VEKD**	This study
pBC366	pBC108	oBC279, oBC554, oBC402, oBC403	**ThfA46**<br>His6-ThfA trivalent monocyclic core peptide	MSGSHHHHHHGSSTAVTDAFPLGRDENRNDQVTEWRPFGMRYGVQPTPIPVPLSDTKYDPDQQVLVVADGQPCAKIERAG**V**MRVTYPDGQKPGQSDVE**ANGPGVMRVTYPDGQKPGQSDVEANGPGVMRVTYPDGQKPGQSDVEAN**	This study
pBC368	pBC108	oBC279, oBC560	**ThfA36**<br>His6-ThfA<br>tricyclic variant	MSGSHHHHHHGSSTAVTDAFPLGRDENRNDQVTEWRPFGMRYGVQPTPIPVPLSDTKYDPDQQVLVVADGQPCAK**ITMRV**T	This study

				**GSG**TYPDGQKPGQSD**GEG**D<br>**VEKD**	
pBC369	pBC108	oBC279,<br>oBC561	**ThfA37**<br>His6-ThfA<br>tricyclic variant	MSGSHHHHHHGSSTAVTDAF<br>PLGRDENRNDQVTEWRPFG<br>MRYGVQPTPIPVPLSDTKYDP<br>DQQVLVVADGQPCAK**ITMRV**T<br>**GSG**TYPDGQKPGQSD**GQG**D<br>**VEKD**	This study
pBC372	pBC108	oBC566,<br>oBC567	His6-mRuby2	MSGSHHHHHHGSVSKGEELI<br>KENMRMKVVMEGSVNGHQF<br>KCTGEGEGNPYMGTQTMRIK<br>VIEGGPLPFAFDILATSFMYGS<br>RTFIKYPKGIPDFFKQSFPEGF<br>TWERVTRYEDGGVVTVMQDT<br>SLEDGCLVYHVQVRGVNFPS<br>NGPVMQKKTKGWEPNTEMM<br>YPADGGLRGYTHMALKVDGG<br>GHLSCSFVTTYRSKKTVGNIK<br>MPGIHAVDHRLERLEESDNEM<br>FVVQREHAVAKFAGLGGGMD<br>ELYK	This study
pAA14	pBC108	oBC279,<br>oAA044,<br>oAA048,<br>oAA052	**ThfA26**<br>His6-ThfA<br>G2T/T3G	MSGSHHHHHHGSSTAVTDAF<br>PLGRDENRNDQVTEWRPFG<br>MRYGVQPTPIPVPLSDTKYDP<br>DQQVLVVADGQPCAKIERA**TG**<br>MRVTYPDGQKPGQSDVEKD	This study
pAA15	pBC108	oBC279,<br>oAA044,<br>oAA047,<br>oAA051	**ThfA28**<br>His6-ThfA<br>T3M/M4T/<br>K21D/D22K	MSGSHHHHHHGSSTAVTDAF<br>PLGRDENRNDQVTEWRPFG<br>MRYGVQPTPIPVPLSDTKYDP<br>DQQVLVVADGQPCAKIERAG<br>**MT**RVTYPDGQKPGQSDVE**DK**	This study
pAA16	pBC108	oBC279,<br>oAA044,<br>oAA046,<br>oAA050	**ThfA27**<br>His6-ThfA<br>V6T/T7V/<br>D18V/V19D	MSGSHHHHHHGSSTAVTDAF<br>PLGRDENRNDQVTEWRPFG<br>MRYGVQPTPIPVPLSDTKYDP<br>DQQVLVVADGQPCAKIERAGT<br>MR**TV**YPDGQKPGQS**VD**EKD	This study
pAA17	pBC108	oBC279,<br>oAA044,<br>oAA045,<br>oAA049	**ThfA25**<br>His6-ThfA<br>T7Y/Y8T/<br>S17D/D18S	MSGSHHHHHHGSSTAVTDAF<br>PLGRDENRNDQVTEWRPFG<br>MRYGVQPTPIPVPLSDTKYDP<br>DQQVLVVADGQPCAKIERAGT<br>MRV**YT**PDGQKPGQ**DS**VEKD	This study
pAA18	pBC108	oBC279,<br>oBC359,<br>oAA053,<br>oBC357	**ThfA20**<br>His6-ThfA<br>R5A	MSGSHHHHHHGSSTAVTDAF<br>PLGRDENRNDQVTEWRPFG<br>MRYGVQPTPIPVPLSDTKYDP<br>DQQVLVVADGQPCAKIERAGT<br>M**A**VTYPDGQKPGQSDVEKD	This study
pAA19	pBC108	oBC279,<br>oAA054	**ThfA21**<br>His6-ThfA<br>E20A	MSGSHHHHHHGSSTAVTDAF<br>PLGRDENRNDQVTEWRPFG<br>MRYGVQPTPIPVPLSDTKYDP	This study

				DQQVLVVADGQPCAKIERAGTMRVTYPDGQKPGQSDV**A**KD	
pHE19	pRSF-duet	oHE1, oHE2, oHE3, oHE4	**ThfA14**<br>His6-ThfA<br>T3S	MSGSHHHHHHGSSTAVTDAFPLGRDENRNDQVTEWRPFGMRYGVQPTPIPVPLSDTKYDPDQQVLVVADGQPCAKIERAG**S**MRVTYPDGQKPGQSDVEKD	This study
pHE20	pRSF-duet	oHE1, oHE2, oHE5, oHE4	**ThfA15**<br>His6-ThfA<br>T7S	MSGSHHHHHHGSSTAVTDAFPLGRDENRNDQVTEWRPFGMRYGVQPTPIPVPLSDTKYDPDQQVLVVADGQPCAKIERAGTMRV**S**YPDGQKPGQSDVEKD	This study
pHE30	pRSF-duet	oHE1, oHE6	**ThfA17**<br>His6-ThfA<br>D22E	MSGSHHHHHHGSSTAVTDAFPLGRDENRNDQVTEWRPFGMRYGVQPTPIPVPLSDTKYDPDQQVLVVADGQPCAKIERAGTMRVTYPDGQKPGQSDVEK**E**	This study
pHE31	pRSF-duet	oHE1, oHE7	**ThfA16**<br>His6-ThfA<br>D18E	MSGSHHHHHHGSSTAVTDAFPLGRDENRNDQVTEWRPFGMRYGVQPTPIPVPLSDTKYDPDQQVLVVADGQPCAKIERAGTMRVTYPDGQKPGQS**E**VEKD	This study
pTGJ023	pBC108	oTGJ026, oBC401	**ThfA**<br>His6-ThfA<br>C73A in leader sequence	MSGSHHHHHHGSSTAVTDAFPLGRDENRNDQVTEWRPFGMRYGVQPTPIPVPLSDTKYDPDQQVLVVADGQP**A**AKIERAGTMRVTYPDGQKPGQSDVEKD	This study
pTGJ028	pBC108	oTGJ026, oTGJ061, oTGJ062, oTGJ023	His6-ThfA<br>T3D/M4V/R5E/V6K/T6D	MSGSHHHHHHGSSTAVTDAFPLGRDENRNDQVTEWRPFGMRYGVQPTPIPVPLSDTKYDPDQQVLVVADGQPCAKIERAG**DVEKD**YPDGQKPGQSDVEKD	This study
pTGJ029	pBC108	oTGJ026, oTGJ063	**ThfA13**<br>His6-ThfA<br>T3D/M4V/R5E/V6K/T6D/D18T/V19M/E20R/K21V/D22T	MSGSHHHHHHGSSTAVTDAFPLGRDENRNDQVTEWRPFGMRYGVQPTPIPVPLSDTKYDPDQQVLVVADGQPCAKIERAG**DVEKD**YPDGQKPGQS**TMRVT**	This study
pBC379	pRSF-duet	oBC576, oBC577, oBC578, oBC579	**ThfA50-coil1**<br>His6-ThfA leader sequence-core peptide (1-7)-6G linker-BECN1 coil<br><br>In MCS-1	MGSSHHHHHHSQDPMSTAVTDAFPLGRDENRNDQVTEWRPFGMRYGVQPTPIPVPLSDTKYDPDQQVLVVADGQPCAKIERAGTMRVT**GGGGGGGPGSDSEQLQRELKELALEEERLIQELEDVEKNRKVVAENLEKVQAEAERLDQEEAQYQREYSEFKRQQLELDDELKSVENQMRYAQMQLDKLKKK**	This study

pBC401	pBC379	oBC618,<br>oBC619	**ThfA50-coil1**<br>(in MCS-1)<br><br>**ThfA50-coil2**<br>(in MCS-2)<br>His6-ThfA leader sequence-core peptide (1-7)-6G linker-BECN1 coil	**ThfA50-coil1**<br>MGSSHHHHHHSQDPMSTAVT<br>DAFPLGRDENRNDQVTEWRP<br>FGMRYGVQPTPIPVPLSDTKY<br>DPDQQVLVVADGQPCAKIERA<br>GTMRVT**GGGGGGGPGSDSE**<br>**QLQRELKELALEEERLIQELE**<br>**DVEKNRKVVAENLEKVQAEA**<br>**ERLDQEEAQYQREYSEFKRQ**<br>**QLELDDELKSVENQMRYAQM**<br>**QLDKLKKK**<br><br>**ThfA50-coil2**<br>**MGPGSDSEQLQRELKELALE**<br>**EERLIQELEDVEKNRKVVAEN**<br>**LEKVQAEAERLDQEEAQYQR**<br>**EYSEFKRQQLELDDELKSVE**<br>**NQMRYAQMQLDKLKKKGGG**<br>**GGG**DVEKD	This study

References for Supplemental Information

1. Choi, B.; Elashal, H. E.; Cao, L.; Link, A. J., Mechanistic Analysis of the Biosynthesis of the Aspartimidylated Graspetide Amycolimiditide. *J Am Chem Soc* **2022,** *144* (47), 21628-21639.
2. Elashal, H. E.; Koos, J. D.; Cheung-Lee, W. L.; Choi, B.; Cao, L.; Richardson, M. A.; White, H. L.; Link, A. J., Biosynthesis and characterization of fuscimiditide, an aspartimidylated graspetide. *Nat Chem* **2022,** *14* (11), 1325-1334.
3. Choi, B.; Acuña, A.; Koos, J. D.; Link, A. J., Large-scale Bioinformatic Study of Graspimiditides and Structural Characterization of Albusimiditide. *ACS Chem Biol* **2023**.
4. Niedermeyer, T. H.; Strohalm, M., mMass as a software tool for the annotation of cyclic peptide tandem mass spectra. *PLoS One* **2012,** *7* (9), e44913.
5. Pbs(P). *Cold Spring Harbor Protocols* **2009,** *2009* (1).
6. Cao, L.; Elashal, H. E.; Link, A. J., Kinetics of Aspartimide Formation and Hydrolysis in Lasso Peptide Lihuanodin. *Biochemistry* **2023,** *62* (3), 695-699.
7. Ngoka, L. C.; Gross, M. L., A nomenclature system for labeling cyclic peptide fragments. *J Am Soc Mass Spectrom* **1999,** *10* (4), 360-3.
