# Supplementary material for: Peptide and Protein Cyclization by a Promiscuous Graspetide Synthetase
Source: ACS Cent Sci. 2025 Jun 9;11(7):1111–21. doi: 10.1021/acscentsci.5c00408 (PMC12291112; doi:10.1021/acscentsci.5c00408)
Supplement: Supplementary file 2 [file oc5c00408_si_002.pdf]

oc-2025-00408j.R1

Name: Peer Review Information for "Peptide and Protein Cyclization by a Promiscuous Graspetide Synthetase"

First Round of Reviewer Comments

Reviewer: 1

Comments to the Author

The manuscript from Choi et al., describe an engineering approach to exploit an enzyme involved in the biosynthesis of the RiPP from the graspetide class to facilitate ester linkages in different peptide and ultimately protein substrates. The work leverages the promiscuity of the enzyme ThfB to introduce conformational restraints in substrates of interest and therefore would be appealing to the broad readership of ACS Central Science. However, there are some technical items of note that must be addressed before the manuscript may be accepted.

1. A major discovery here is that ThfB can install crosslinks in peptide substrates with intervening loops are large as 72 amino acids. A concern with larger peptide substrates is that the linkage might occur intermolecularly rather than intramolecularly. The authors can rule this out by testing the enzyme with two peptide substrates: one containing residues 1-7 of the recognition site and the other containing residues 18-22 to show whether or not the amide linkages can be installed in trans. Regardless of the outcome of this experiment, the results would add to the current story substantially.

2. The use of ThfB for crosslinking protein substrates may be of broad interest but the current version of the manuscript does not elaborate on this point. Perhaps the authors can enhance this aspect by testing the (thermal?) stability of the crosslinked protein vs. the unlinked variant?

Minor points:

1. The choice of the color scheme for Figure S17 is unfortunate as it is difficult to discern the light blue from blue (and light green from green). The authors are encouraged to change the colors so that casual readers may more easily appreciate aspects of this figure.

2. In the main text, please explain what ffGFP stands for (i.e. superfolder GFP).

3. Page S2: please capitalize Golden Gate assembly.

Reviewer: 2

Comments to the Author

In this paper, the authors analyze the substrate specificity of ThfB, which is involved in the biosynthesis of the cyclic peptide Fuscimiditide, and reveal that its substrate specificity is highly promiscuous. Notably, ThfB exhibits low substrate specificity in the loop of fuscimiditide, allowing even proteins to be inserted. The substrate tolerance of ThfB elucidated in this paper is a significant discovery, as it can be applied not only to peptide modification but also to protein engineering. The text is easy to understand, the analyses are thorough overall, and the resulting data are convincing. However, the studies in this paper are limited to substrate specificity and do not analyze the effects of its modifications. Therefore, the utility or advantage of ThfB-catalyzed modification remains unclear. Furthermore, it is difficult to say that this paper approaches the mechanism of ThfB's tolerant substrate recognition. Thus, the reviewer believes this paper is currently insufficient for publication in ACS Central Science and requires the following improvements.

Is it possible to assert the effectiveness of this enzyme by verifying what properties are imparted by this macrocyclization? Have any new antibacterial agents been obtained? Or how about analyzing whether the physical properties of the fluorescent protein are imparted with thermal stability or peptidase resistance?

The pLDDT of the predicted structure in AF3 should be shown. In particular, the reliability around the binding site is very important information. If the predicted structure around the binding site is not reliable, it may suggest that the substrate specificity is unclear. Also, are there differences in the binding modes of the core peptide among the predicted structures at various ranks? If they are different, this may also be considered data supporting the loose substrate specificity.

In this paper, the structure of the fluorescent protein is discussed without the leader peptide. Shouldn't the discussion be based on the predicted structure with the leader peptide, which is the actual reaction substrate?

minor point

Page 6, Line 12: Figure 1E —> Figure 3D

Page 6, Line 42: Figure 4A —> Figure 4B

Reviewer: 3

Comments to the Author

The RIPPS natural product class is a practical genetic system for applications in natural product engineering. The discovery of novel chemistry and the exploitation of substrate scope are key to advancing future engineering efforts. This manuscript describes the characterization and substrate limitations/tolerance of the ThfB ATP-grasp enzyme. The authors previously identified a unique family of RIPP graspetides and characterized the cyclase enzyme ThfB, which adds two macrolactones to a precursor peptide, and the authors also published mechanistic studies of a similar enzyme of this family.

Overall, the work is comprehensive and provides extensive experiments to probe the specificity range of the chemistry. Several illustrated examples of variant, cyclization motifs are novel to the graspeptide family. The manuscript is well written and results are explained well.

As mentioned a few times, the work is not really engineering or probing sequence tolerance of the natural product fuscimiditide biosynthesis, as the chemistry of downstream biosynthetic enzymes is not examined.

Cyclization of a linker serine was unusual. It would be interesting to scan the series with Ala to probe and confirm specificity and/or include mixed, randomized sequences to probe whether the GS motif is a requirement for the promiscuity of ring size tolerance. Regiospecificity was probed with select examples with MS/MS.

A major issue that I found was the reliance on (largely) one analytical approach to assess the chemistry. Confirming the chemistry in vitro with purfied protein and peptide substrates is certainly something the authors can do. Some kinetic-type data would nicely support the experiments in the manuscript. In addition, these general assay conditions can be better described in the text and supplementary information. For example, to get the details of the heterologous plasmid constructs and the general assay approach, the reader needs to refer to supporting information of previous publications.

I didn’t find the presented alphafold modelsto be at all informative; the inherent limitations of the alphafold program make valid interpretations difficult.

Author's Response to Peer Review Comments:

Response to the reviewers

Our responses are in **bold**

Reviewer: 1

The manuscript from Choi et al., describe an engineering approach to exploit an enzyme involved in the biosynthesis of the RiPP from the graspetide class to facilitate ester linkages in different peptide and ultimately protein substrates. The work leverages the promiscuity of the enzyme ThfB to introduce conformational restraints in substrates of interest and therefore would be appealing to the broad readership of ACS Central Science. However, there are some technical items of note that must be addressed before the manuscript may be accepted.

1. A major discovery here is that ThfB can install crosslinks in peptide substrates with intervening loops are large as 72 amino acids. A concern with larger peptide substrates is that the linkage might occur intermolecularly rather than intramolecularly. The authors can rule this out by testing the enzyme with two peptide substrates: one containing residues 1-7 of the recognition site and the other containing residues 18-22 to show whether or not the amide linkages can be installed in trans. Regardless of the outcome of this experiment, the results would add to the current story substantially.

We considered the possibility of intermolecular crosslinking between 2 or more molecules of ThfA9 and ThfA10, but careful analysis of our mass spectrometry data showed no evidence of these higher order oligomers. We have added a sentence about this to the main text. While we have not carried out exactly the experiment suggested by the reviewer due to difficulties in expressing a short fragment comprising residues 18-22 of pre-fuscimiditide, we have added new data (Figure 8) showing that our system does work in a supramolecular fashion. Namely, a non-covalent coiled-coli interaction can bring together the two stem fragments in different protein chains and ThfB can crosslink those chains. We think that this new data strengthens our point about the promiscuity and versatility of ThfB.

2. The use of ThfB for crosslinking protein substrates may be of broad interest but the current version of the manuscript does not elaborate on this point. Perhaps the authors can enhance this aspect by testing the (thermal?) stability of the crosslinked protein vs. the unlinked variant?

While the main focus of this work was to demonstrate the broad applicability of ThfB as a protein and peptide cyclization biocatalyst, we agree with the reviewer that exploring any benefits of cyclization is interesting too. With regard to the cyclized peptide constructs (for example ThfA1-Thf10 with loop substitutions) we point out that these constructs retain the resistance to trypsin that is a hallmark of the wild-type pre-fuscimiditide. A sentence has been added to this effect in the section describing the MS2 analysis and hydrazinolysis of mThfA3^{B}. With regard to the cyclized proteins, in retrospect perhaps we should have chosen a demonstration system that is more thermally labile because fluorescent proteins generally remain fluorescent (and folded) up to 80 °C. Figure S14 of the original submission showed that there is minimal effect on the fluorescence of mRuby2 upon cyclization by ThfB. We have added a new SI figure, S15, showing that loss of fluorescence of mRuby2 upon heating at 90 °C is essentially the same for the cyclized and uncyclized proteins. So, while with this example we have not been able to show any improvement in properties upon cyclization, we at least show that existing fluorescence and thermostability are not disrupted upon cyclization. We now describe this experiment in the main text.

Minor points:

1. The choice of the color scheme for Figure S17 is unfortunate as it is difficult to discern the light blue from blue (and light green from green). The authors are encouraged to change the colors so that casual readers may more easily appreciate aspects of this figure.

We thank the reviewer for highlighting this to us and have amended the coloring of this figure accordingly (now Figure S19).

2. In the main text, please explain what ffGFP stands for (i.e. superfolder GFP).

It turns out that ffGFP was just an internal name for this protein, which is indeed a variant of sfGFP. We have called it sfGFP throughout the text and SI now and have also added the full protein sequence (and other protein sequences/accession numbers) to the first page of the SI.

3. Page S2: please capitalize Golden Gate assembly.

We thank the reviewer for highlighting this to us and have amended this in the supporting information.

Additional Questions:
Quality of experimental data, technical rigor: Top 5%

Significance to chemistry researchers in this and related fields: Top 1%

Broad interest to other researchers: Top 1%

Novelty: Top 5%

Is this research study suitable for media coverage or a First Reactions (a News & Views piece in the journal)?: No

Reviewer: 2

Recommendation: Publish elsewhere ACS Catalysis

Comments:
In this paper, the authors analyze the substrate specificity of ThfB, which is involved in the biosynthesis of the cyclic peptide Fuscimiditide, and reveal that its substrate specificity is highly promiscuous. Notably, ThfB exhibits low substrate specificity in the loop of fuscimiditide, allowing even proteins to be inserted. The substrate tolerance of ThfB elucidated in this paper is a significant discovery, as it can be applied not only to peptide modification but also to protein engineering. The text is easy to understand, the analyses are thorough overall, and the resulting data are convincing. However, the studies in this paper are limited to substrate specificity and do not analyze the effects of its modifications. Therefore, the utility or advantage of ThfB-catalyzed modification remains unclear. Furthermore, it is difficult to say that this paper approaches the mechanism of ThfB's tolerant substrate recognition. Thus, the reviewer believes this paper is currently insufficient for publication in ACS Central Science and requires the following improvements.

Is it possible to assert the effectiveness of this enzyme by verifying what properties are imparted by this macrocyclization? Have any new antibacterial agents been obtained? Or how about analyzing whether the physical properties of the fluorescent protein are imparted with thermal stability or peptidase resistance?

In our response to reviewer 1 above on a similar point, we noted that for short cyclic peptides, protease resistance is endowed to these constructs by virtue of the cyclization. While such proteolytic stability is not unexpected, it nonetheless

shows a property imparted by cyclization. As also described above in response to reviewer 1, we measured the resistance to a thermal challenge (90 °C) for the cyclized mRuby protein. This protein is already very thermally stable, maintaining fluorescence until ~80 °C, so cyclization did not have a further stabilizing effect (see new Figure S15). Sentences describing both of these effects have been added to the text. Finally, the new data presented in Figure 8 shows that we can transform a non-covalently interacting pair of proteins into a covalently-linked assembly, yet another property change enabled by ThfB.

The pLDDT of the predicted structure in AF3 should be shown. In particular, the reliability around the binding site is very important information. If the predicted structure around the binding site is not reliable, it may suggest that the substrate specificity is unclear. Also, are there differences in the binding modes of the core peptide among the predicted structures at various ranks? If they are different, this may also be considered data supporting the loose substrate specificity.

Thanks for the suggestion to examine more closely the pLDDT of our AF3 model. Indeed, while AF3 is confident in its prediction of the ThfB enzyme structure, it has only poor confidence in the structure of the precursor ThfA. We now mention this in the main text and have added a new Figure S20 showing the pLDDT explicitly for the top model prediction of AF3. We have added some text to the discussion citing this figure and further solidifying our point that, at least for now, the AF3 models are not much use in predicting the binding of ThfA to ThfB. While our figures show only the top AF3 model, other models show the core and leader peptide interacting with ThfB in similar poses.

In this paper, the structure of the fluorescent protein is discussed without the leader peptide. Shouldn't the discussion be based on the predicted structure with the leader peptide, which is the actual reaction substrate?

In Figure 7D we show an AF3 model of the "leaderless" mRuby construct to give a sense of how large the stem regions of fuscimiditide are with respect to the protein. Our Figure S13 in the original paper included models for all 3 proteins, with and without leaders. The caption of Figure 7D now points to Figure S13 and the caption to Figure S13 has been updated with pLDDT information for the leader peptide.

minor point
Page 6, Line 12: Figure 1E —> Figure 3D
Page 6, Line 42: Figure 4A —> Figure 4B

We have corrected these typos

Additional Questions:
Quality of experimental data, technical rigor: Top 5%

Significance to chemistry researchers in this and related fields: High

Broad interest to other researchers: High

Novelty: Moderate

Is this research study suitable for media coverage or a First Reactions (a News & Views piece in the journal)?: No

Reviewer: 3

Recommendation: Publish in ACS Central Science after minor revisions noted.

Comments:
The RIPPS natural product class is a practical genetic system for applications in natural product engineering. The discovery of novel chemistry and the exploitation of substrate scope are key to advancing future engineering efforts. This manuscript describes the characterization and substrate limitations/tolerance of the ThfB ATP-grasp enzyme. The authors previously identified a unique family of RIPP graspetides and characterized the cyclase enzyme ThfB, which adds two macrolactones to a precursor peptide, and the authors also published mechanistic studies of a similar enzyme of this family.
Overall, the work is comprehensive and provides extensive experiments to probe the specificity range of the chemistry. Several illustrated examples of variant, cyclization motifs are novel to the graspeptide family. The manuscript is well written and results are explained well.
As mentioned a few times, the work is not really engineering or probing sequence tolerance of the natural product fuscimiditide biosynthesis, as the chemistry of downstream biosynthetic enzymes is not examined.

We thank the reviewer for bringing up this point. The reviewer is correct in that we are truly engineering pre-fuscimiditide rather than fuscimiditide. Indeed, many of the constructs we analyzed in this paper would no longer be substrates for the methyltransferase enzyme ThfM that converts pre-fuscimiditide into fuscimiditide. Therefore we have carefully gone through the manuscript and have changed many instances of "fuscimiditide" to "pre-fuscimiditide" when the context is about engineering the peptide. This even resulted in small changes to the abstract. While it is a little clunkier to say "pre-fuscimiditde" than "fuscimiditide," we prefer the preciseness that this change provides.

Cyclization of a linker serine was unusual. It would be interesting to scan the series with Ala to probe and confirm specificity and/or include mixed, randomized sequences to probe whether the GS motif is a requirement for the promiscuity of ring size tolerance. Regiospecificity was probed with select examples with MS/MS.

We have expanded this section of the text slightly to point out that we did examine the regioselectivity for the ThfA9 construct and observed some of the desired product. We also emphasize that we observed a specific crosslink between D18 and only one Ser in the linker. While we agree with the reviewer that libraries of sequences would be interesting (and we propose this type of experiment in our conclusion section), we believe that such experiments are beyond the scope of the current paper.

A major issue that I found was the reliance on (largely) one analytical approach to assess the chemistry. Confirming the chemistry in vitro with purfied protein and peptide substrates is certainly something the authors can do. Some kinetic-type data would nicely support the experiments in the manuscript. In addition, these general assay conditions can be better described in the text and supplementary information. For example, to get the details of the heterologous plasmid constructs and the general assay approach, the reader needs to refer to supporting information of previous publications.

We have previously demonstrated that we can reconstitute the full fuscimiditide pathway *in vitro*, as mentioned in the conclusion section of the paper, and thus we expect that these alternative precursors sequences will also be accepted *in vitro*. Therefore we have chosen not to undertake further kinetics experiments in this manuscript.

While we agree with the reviewer that our workhorse characterization method here is mass spectrometry, we do carry out several other analytical techniques (hydrazinolysis, for example) to confirm the connectivity of the peptides. We also

use further techniques such as fluorescence measurements to characterize the products of the ThfB reaction. While we have previously solved NMR structures of pre-fuscimiditide and fuscimiditide, those studies are involved and time-consuming, and thus we chose not to characterize any of the variants in this study via NMR.

To address the concerns about the methods, we had added more details in the SI about plasmid construction, especially about the new supramolecular construct shown in Figure 8. While it is impractical for us to write a paragraph about each of the 50 constructs in the paper, Tables S10 and S11 have all of the primer sequences and plasmid construction details for each construct. We have also added protein sequences and accession numbers to the first page of the methods so that readers will not have to look up these sequences in our prior work.

I didn't find the presented alphafold models to be at all informative; the inherent limitations of the alphafold program make valid interpretations difficult.

We have used alphafold in two places in our manuscript. First, in Figure 7D/S13, we used alphafold just to give a sense of the scale of the fuscimiditide stem "tag" relative to the fluorescent protein. The caption of Figure S13 now has a statement about the low pLDDT values in the leader peptide predictions. The other use of alphafold is in the conclusion section, where we make the point (in agreement with this reviewer) that the alphafold model is not helpful in understanding the substrate specificity of ThfB. In response to reviewer 2, we have included additional information about the pLDDT of this model (Figure S20), which shows low confidence in the prediction of the entire ThfA precursor. So, while we agree with the reviewer that the alphafold models (especially of the ThfA/ThfB complex) are not particularly helpful, we have left them in to contrast with the new insights we gain from the experimental work in this study on the broad substrate specificity of ThfB.

Additional Questions:
Quality of experimental data, technical rigor: Top 5%

Significance to chemistry researchers in this and related fields: High

Broad interest to other researchers: Moderate

Novelty: High

Is this research study suitable for media coverage or a First Reactions (a News & Views piece in the journal)?: No
